# Supplementary material for: A bioinspired sequential energy transfer system constructed via supramolecular copolymerization
Source: Nat Commun. 2022 Jun 21;13:3546. doi: 10.1038/s41467-022-31094-w (PMC9213434; doi:10.1038/s41467-022-31094-w)
Supplement: Supplementary file 1 — Supplementary Information [file 41467_2022_31094_MOESM1_ESM.pdf]

Supplementary Information

A Bioinspired Sequential Energy Transfer System Constructed *via* Supramolecular Copolymerization

Y. Han *et al.*

Supplementary Methods

Reagents and reactants: For the synthesis of monomers **1**–**3**, the starting compounds and reagents such as 9,10-anthracenedione, 5,12-naphthacenequinone, (triisopropylsilyl)acetylene, stannous chloride ($SnCl_2$), *n*-butyllithium (*n*-BuLi, 1.6 mol L^{-1} in *n*-hexane), tetrabutylammonium fluoride (TABF, 1 mol L^{-1} in THF), 2,3-dichloro-1,4-naphthoquinone, potassium phthalimide, hydrazine hydrate, thionyl chloride ($SOCl_2$), lithium aluminium hydride ($LiAlH_4$), sodium hypophosphite ($Na_2H_2PO_2$), selemium dioxide (SeO_2), copper(I) iodide (CuI), and diisopropylamine ((*i*-Pr)$_2$NH) were purchased from commercial sources and used without further purifications. *Trans*-[Pt(PEt_3)$_2$]I_2[1], compound **4**[2], triisopropylsilyl substituted dialkynyl(hetero)acenes **7**–**9**[3–5], the intermediate compounds **10**–**12**[6], and compound **1**[6] were synthesized according to the reported literatures. All solvents involved in preparation and purification procedures were employed as purchased.

DFT and TDDFT theoretical calculations: Gaussian 09 software package was exploited to perform theoretical computations[7]. B3LYP exchange functional was chosen to calculate the optimize geometries of **1**–**3**. Meanwhile, non-metallic elements (C, H, O, N, Se, and P) were described by 6-31G(d) basis set, while Lanl2dz effective core potential developed by Los Alamos National Laboratory was used to describe Pt(II) ions. To study the electronic transitions, TDDFT calculations were performed at the same computational level without adding solvation model. Besides, to gain deeper insights into the assembly driving forces, dispersion-corrected exchange functional *ω*b97xd was exploited to optimize geometries of trimeric species. To reduce the computational costs of optimized dimers and trimers, the basis sets for non-metallic elements were decreased to 6-31G computational level. Frequency calculations confirmed that imagery frequencies were absent for these optimized geometries.

Determination of fluorescent quantum yields: Absorption and emission spectra of **1**–**3** were carried out on a UV–1800 Shimadazu spectrometer and a FluoroMax-4 spectrofluorometer, respectively. Coumarin 153 (Φ_F = 0.544 in ethanol)[8] and rhodamine B (Φ_F = 0.710 in ethanol)[9] were exploited as standard references for the quantum yields of **1** and **2**. Considering that the emission signal of **3** locates in the NIR region, cryptocyanine (Φ_F = 0.012 in ethanol) was used as the standard reference for the determination of quantum yield[8].

Determination of thermodynamic parameters for the supramolecular polymerization process: To gain deeper insights into the thermodynamic parameters of supramolecular polymers,

temperature-dependent CD and absorption experiments have been carried out to obtain the melting curves. The melting curves are fitted *via* the mass balance models developed by Markvoort and ten Eikelder[10,11]. By using this model, it provides critical elongation temperatures (T_e), enthalpy changes (ΔH), entropy changes (ΔS), and nucleation penalties (NP) for supramolecular polymerization processes. The cooperativity parameter (σ) is calculated *via* the following equation:

$$\sigma = \exp[NP/(R \times T)] \qquad \textbf{(Supplementary Eq. 1)}$$

For the equation, T denotes the evaluated temperature for supramolecular polymerization, while R is the universal gas constant (R = 8.314 J mol^{-1} K^{-1}).

Calculation of energy transfer efficiencies, rates and the averaged D/A distances: To confirm that energy transfer from donor to acceptor, the spectral overlap integral $J(\lambda)$ between D/A pairs was calculated based on the supplementary Equation 2[12]:

$$J(\lambda) = [\int_0^\infty F_D(\lambda)\varepsilon_A(\lambda)\lambda^4\, d\lambda]/[\int_0^\infty F_D(\lambda)\, d\lambda]$$

(**Supplementary Eq. 2**)

In this equation, F_D (λ) is the fluorescence emission spectrum of donor, whiles ε_A (λ) is the molar absorption coefficient. λ denotes the wavelength of absorption or emission spectrum. F_D (λ) is a dimensionless term in the formula and the value of denominator is normalized to 1 before calculation. On this basis, the integral overlap can be exploited to judge whether or not energy transfer occurs for D/A pairs. On this basis, the Fröster radius (R_0) of energy transfer system can be calculated as follows[12]:

$$R_0 = 0.0211(n^{-4}\kappa^2\Phi_D J(\lambda))^{1/6} \qquad \textbf{(Supplementary Eq. 3)}$$

In supplementary Equation 3, Φ_D stands for the quantum yield of donor in the absence of acceptor. n is the refraction index of assembly solution. $J(\lambda)$ is the spectral overlap integral of a D/A pair, and κ^2 stands for the orientation factors (κ^2 = 2/3, random orientation).

To quantitatively evaluate the energy transfer property, we calculate the energy transfer efficieny (Φ_{ET}) of donors based on the following equation[12]:

$$\Phi_{ET} = 1 - I_{D/A,\ (\lambda ex\ =\ donor)}/I_{D,\ (\lambda ex\ =\ donor)} \qquad \textbf{(Supplementary Eq. 4)}$$

In supplementary Equation 4, $I_{D/A}$ stands for the donor fluorescence emission intensities of supramolecular copolymers, while I_D stands for the donor fluorescence emission intensities of supramolecular homopolymers under the same excitation conditions.

Φ_{ET} was further employed to determine the energy transfer rate constant (k_{ET}) under different acceptor loading amounts according to supplementary Equation 5[12]:

$$\Phi_{ET} = k_{ET}/(k_r + k_{nr} + k_{ET}) \qquad \textbf{(Supplementary Eq. 5)}$$

In the equation, k_r and k_{nr} denote radiative and non-radiative decay constant of donor, respectively. k_{ET} represents energy transfer rate constant. The sum of k_r and k_{nr} is equal to the reciprocal of donor fluorescence lifetime in the absence of acceptor.

Besides, k_{ET} rates also can be determined via the supplementary Equation 6[12]:

$$k_{ET} = (1/\tau_D)\times(R_0/d)^6 \qquad \textbf{(Supplementary Eq. 6)}$$

In the equation, τ_D refers to the fluorescence lifetime of donors in the supramolecular homopolymeric state. d stands for the average distance of D/A pair. R_0 stands for the Fröster radius of D/A pair. τ_D is obtained by measuring the fluorescence lifetime of donor, while R_0/d are calculated based on the supplementary Equation 7[12]:

$$\Phi_{ET} = R_0^6/(R_0^6 + d^6) \qquad \textbf{(Supplementary Eq. 7)}$$

For the equation, R_0/d can be calculated once the energy transfer efficiency (Φ_{ET}) is already known. In combination with the supplementary Equation 3, the average distance (d) of a D/A pair is obtained. The supplementary equation 7 is only suitable to calculate d value of a binary D/A supramolecular copolymeric system with energy transfer.

For the ternary supramolecular copolymers **1**/**2**/**3** with sequential energy transfer character, d value between D/A pair is calculated via the following equation[12,13]:

$$d_{\mathbf{1/j}} = R_{0(\mathbf{1/j})}[(1 - \Phi'_{\mathbf{1/j}} - \Phi'_{\mathbf{1/k}})/\Phi'_{\mathbf{1/j}}]^{1/6} \qquad \textbf{(Supplementary Eq. 8)}$$

In supplementary Equation 8, $R_{0(\mathbf{1/j})}$ refers to the Förster radius of the competitive D/A pairs (j, k = **2** or **3**), which can be derived from the above binary supramolecular copolymeric systems. $\Phi'_{\mathbf{1/j}}$ and $\Phi'_{\mathbf{1/k}}$ represent the apparent energy transfer efficiencies. The apparent energy transfer efficiency from **1** to the acceptor **j** can be written as follows:

$$\Phi'_{\mathbf{1/j}} = k_{\mathbf{1/j}}/(k_{r\mathbf{1}} + k_{nr\mathbf{1}} + k_{\mathbf{1/j}} + k_{\mathbf{1/k}}) \qquad \textbf{(Supplementary Eq. 9)}$$

In supplementary Equation 9, $k_{r\mathbf{1}}$ and $k_{nr\mathbf{1}}$ stand for radiative and non-radiative decay rate of **1** in the absence of acceptors, respectively. The sum of $k_{r\mathbf{1}}$ and $k_{nr\mathbf{1}}$ is equal to the reciprocal of fluorescence lifetime of supramolecular homopolymers **1**. $k_{\mathbf{1/j}}$ and $k_{\mathbf{1/k}}$ denote the energy transfer rates for the corresponding binary supramolecular copolymeric systems. Once $\Phi'_{\mathbf{1/j}}$ and $\Phi'_{\mathbf{1/k}}$ are determined, the d value for a specific D/A pair in the ternary supramolecular copolymers can be calculated on the basis of supplementary Equation 8.

Calculation of the antenna effect: To evaluate light harvesting capability, we calculated the antenna effect (AE) of the acceptor units via the following equation[12]:

$$AE = (I_{DA,\lambda 1} - I_{D,\lambda 1})/I_{A,\lambda 2} \qquad \textbf{(Supplementary Eq. 10)}$$

In supplementary Equation 10, $I_{DA,\lambda 1}$ and $I_{D,\lambda 1}$ refer to fluorescent emission intensities of supramolecular copolymers D/A and supramolecular homopolymers D, respectively, at the

maximal emission wavelengths of acceptors upon excitation at λ_1 wavelength. $I_{A,\lambda 2}$ stands for the fluorescence intensity of acceptors upon direct excitation at the wavelength of λ_2.

Stern–Volmer analysis for energy transfer systems: Two different *Stern–Volmer* equations were used to analyze exciton migration behaviors. For supramolecular copolymers **1**/**2**, the fluorescence quenching of donor units was analyzed by the following equation[14]:

$$I_0/I = 1 + K_{SV} \times [Q] \qquad \textbf{(Supplementary Eq. 11)}$$

For supplementary Equation 11, I_0 and I denote the fluorescence intensities in the absence and presence of acceptor, respectively. [Q] stands for the acceptor (or called quencher) concentration, while K_{SV} is the *Stern–Volmer* constant.

For supramolecular copolymers **2**/**3**, the *Stern–Volmer* plot of I_0/I versus the acceptor concentration deviates from linear tendency. Therefore, a modified *Stern–Volmer* equation reported by Sorokin and co-workers was used to analyze the exciton migration behaviors[14]:

$$I_0/(I_0 - I) = 1/(f_q \times K_{SV} \times [Q]) + 1/f_q \qquad \textbf{(Supplementary Eq. 12)}$$

In supplementary Equation 12, f_q stands for the percentage of excitons trapped by acceptors during the exciton migration. The values of K_{SV} and f_q can be calculated from the slope and the intercept of the linearly fitted plots.

Determination of second-order exciton migration rates: Considering that exciton migration rate is a important parameter to evaluate exciton migration behaviors, we determined the exciton migration rate of the energy transfer systems[15]. According to the supplementary Equations 13 and 14, radiative relaxation and exciton trapping are considered. The detailed processes can be expressed as follows:

$$D^* + A \rightarrow A^* + D \text{ (Reaction rate: } k) \qquad \textbf{(Supplementary Eq. 13)}$$

$$D^* \rightarrow D \text{ (Reaction rate: } k_0) \qquad \textbf{(Supplementary Eq. 14)}$$

In these equations, D and D^* denote the ground state and the exicted state of donors, respectively. A and A^* represent the ground state and the exicted state of acceptor units, respectively. Accordingly, the quenching rate of exciton can be expressed as follows:

$$d[D^*]/dt = -k[D^*][A] - k_0[D^*] \qquad \textbf{(Supplementary Eq. 15)}$$

For the fluorescence lifetime decay measurements, the excitation power of light source is rather low. Hence, it is assumed that the concentration of excited donor is extremely low with respect to that of the ground state acceptor ([A] >> [D^*]). Accordingly, the concentration of excitons can be written in the following form:

$$[D^*] = e^{-1/\tau \cdot t} \qquad \textbf{(Supplementary Eq. 16)}$$

For the supplementary Equation 16, the quenching rate of excitons complies with a monoexponential decay. By plotting the reciprocal of donor fluorescent lifetimes ($1/\tau$) versus the concentration of acceptor, the second-order rate constant for the exciton migration process is equal to the slope of the linearly fitted line.

Determination of the number of donors quenched by a single acceptor in energy transfer systems. Since both dynamic and static quenching participate in the donor quenching process, a model that combines both mechanisms was used to calculate the *n* value[16]. It is assumed that one light harvesting antenna copolymerize with one acceptor by a 1:1 binding isotherm. The expression for the model can be expressed in the following form:

$$(\text{Donor})_n + \text{Acceptor} = (\text{Donor})_n\text{–Acceptor}$$

On this basis, a donor quenching model was employed, which can be expressed by the supplementary Equation 17:

$$I_F = I_0 + ((I_{\lim} - I_0)/2c_0) \times ((c_0 + c_A + 1/K_a) - ((c_0 + c_A + 1/K_a)^2 - 4c_0c_A)^{1/2})$$

(**Supplementary Eq. 17**)

For the equation, I_F is the fluorescent emission intensity of the supramolecular copolymeric system. I_0 is the emission intensity in the absence of acceptor. $I_{\lim}$ is the emission intensity limitation of co-assembled system. c_0 denotes the concentration of $(\text{Donor})_n$, while c_A is the concentration of acceptor. K_a is the association constant on the basis of the 1:1 binding model.

Photo-irradiation experiments: To investigate the photo-triggered modulation of SET, LED lamps (12 W) with different wavelengths (460 nm and 525 nm) were employed to perform the light irradiation experiments. The distance between LED lamps and irradiated samples were kept at 10 cm. To ensure the endoperoxidation of acenes, all samples were saturated with air prior to the irradiation experiments.

*Synthesis of monomers **1**–**3**:*

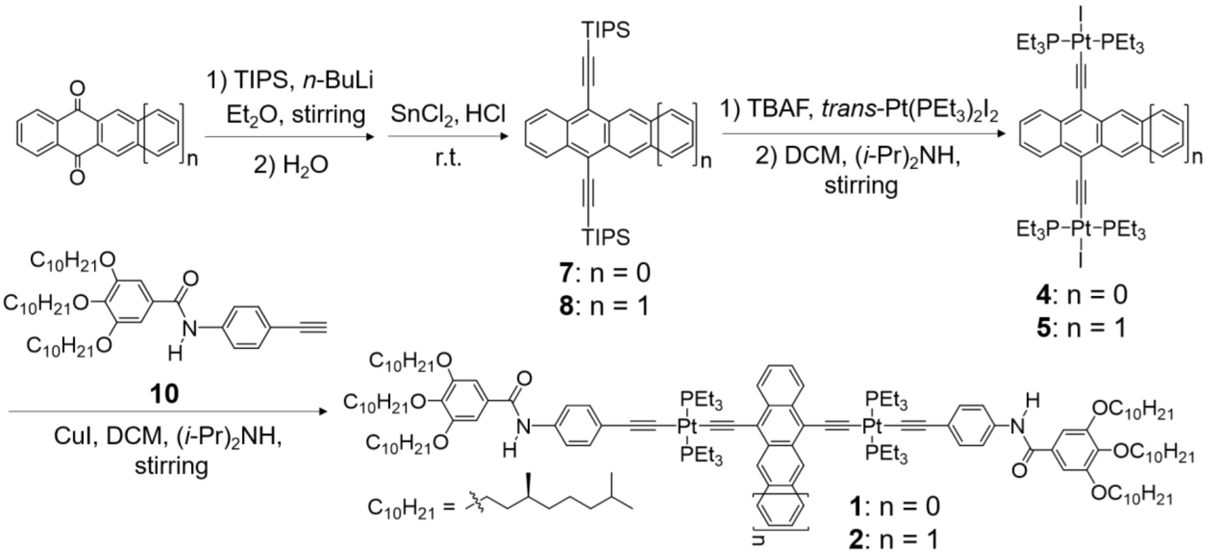

Supplementary Figure 1. Synthetic route to monomers **1** and **2**.

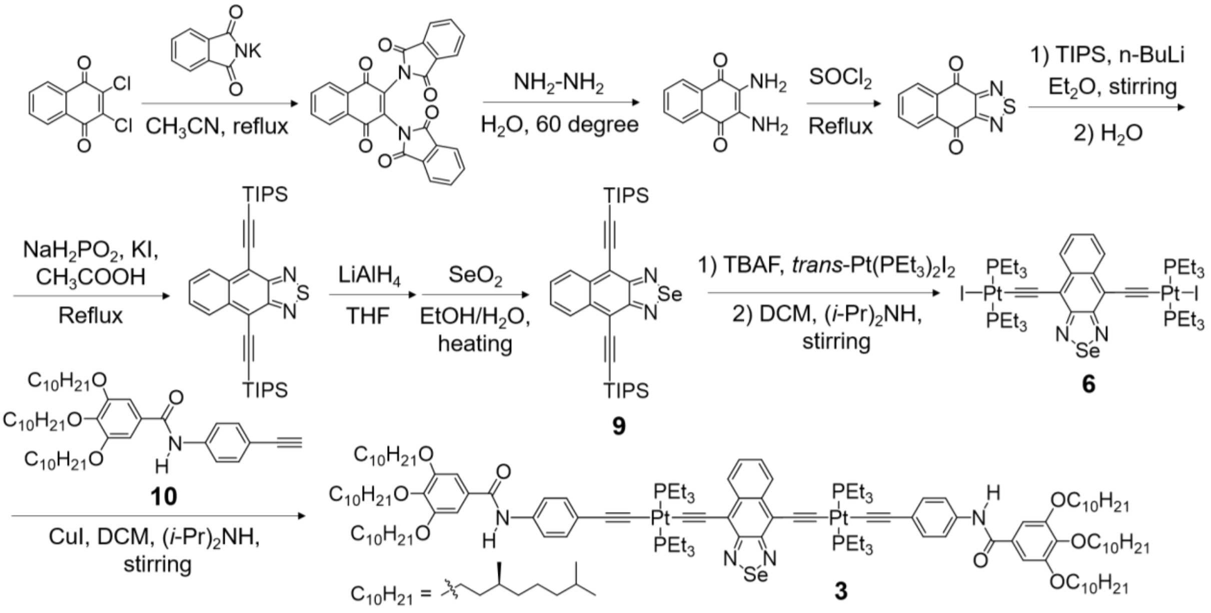

Supplementary Figure 2. Synthetic route to monomer **3**.

*Synthesis of compound **5***

The synthetic procedure was slightly modified according to the procedure reported by Yip[17]: *Trans*-[Pt(PEt_3)$_2$]I_2 (602.5 mg, 0.88 mmol) and CuI (10 mg, 0.052 mmol) were added to a Schlenk flask charged with nitrogen gas. A mixture of CH_2Cl_2 (9.0 mL) and (*i*-Pr)$_2$NH (5.0 mL) were injected into the Schlenk flask via a syringe, followed by the addition of TABF solution (0.8 mL, 1 mol L^{-1} in THF). The flask was subsequently cooled down to –78 °C and the CH_2Cl_2 solution of **8** was added dropwise. Upon completion of the addition, the resulting solution was stirred at room temperature for 48 hours. The reaction mixture was evaporated under vacuum

to afford a dark red solid. The crude product was purified by silica gel column chromatography [eluent: petroleum ether/CH_2Cl_2 = 2 : 1 (*v*/*v*)] to afford pure compound **5** as a dark red solid (143 mg, yield: 53.2%). ^{1}H NMR (400 MHz, $CDCl_3$, 298 K, Supplementary Figure 44) δ 9.34 (s, 2H), 8.70 (dd, *J* = 6.7, 3.4 Hz, 2H), 7.96 (dd, *J* = 6.5, 3.3 Hz, 2H), 7.42–7.38 (m, 4H), 2.28–2.21 (m, 24H), 1.27–1.19 (m, 36H). ^{13}C NMR (101 MHz, $CDCl_3$, 298 K, Supplementary Figure 45) δ 131.52, 131.43, 130.52, 128.61, 128.18, 126.67, 125.21, 125.03, 120.44, 105.21, 98.75, 17.07, 16.90, 16.72, 8.65, 8.54, 8.43. ^{31}P NMR (162 MHz, $CDCl_3$, 298 K, Supplementary Figure 46) δ 9.58 (s, $^1J_{Pt\text{-}P}$ = 2326.32 Hz). MALDI-TOF (Supplementary Figure 47): *m*/*z* 1390.2853, $[M]^+$.

*Synthesis of compound **6***

The synthetic procedures of **6** were similar to that of counterpart **5**, except that compound **9** (460 mg, 0.775 mmol) was utilized instead of **8**. The product was further purified by silica gel column chromatography [eluent: petroleum ether/CH_2Cl_2 = 1.5 : 1 (*v*/*v*)] to afford compound **6** as a blue solid powder (379 mg, yield: 35.0%). ^{1}H NMR (400 MHz, $CDCl_3$, 298 K, Supplementary Figure 48) δ (ppm): 8.45 (dd, *J* = 6.8, 3.3 Hz, 2H), 7.26 (dd, *J* = 6.8, 3.3 Hz, 2H), 2.34–2.27 (m, 24H), 1.22–1.14 (m, 36H). ^{13}C NMR (101 MHz, $CDCl_3$, 298 K, Supplementary Figure 49) δ 159.68, 134.92, 128.50, 125.97, 115.68, 108.59, 99.37, 16.99, 16.81, 16.64, 8.59, 8.48, 8.37. ^{31}P NMR (162 MHz, $CDCl_3$, 298 K, Supplementary Figure 50) δ 9.25 (s, $^1J_{Pt\text{-}P}$ = 2313.36 Hz). MALDI-TOF (Supplementary Figure 51): *m*/*z* 1396.2803, $[M]^+$.

*Synthesis of compound **2***

Compounds **5** (62.2 mg, 0.045 mmol), **10** (71.0 mg, 0.103 mmol) and CuI (4 mg, 0.021 mmol) were added to a Schlenk flask charged with nitrogen. CH_2Cl_2 (10.0 ml) and $(i\text{-Pr})_2NH$ (3.0 ml) were mixed together and injected into the flask via a springe. The solution was stirred under N_2 for 24 hours. Upon completion of the reaction, the solvent was removed under vaccum to obtain the crude product, which was purified via silica gel chromatography [eluent: petroleum ether/ethyl acetate = 6 : 1 (*v*/*v*)] to afford pure **2** as a dark red solid (43.1 mg, yield: 38.1%). ^{1}H NMR (400 MHz, $CDCl_3$, 298 K, Supplementary Figure 52) δ (ppm): 9.38 (s, 2H), 8.74 (dd, *J* = 6.7, 3.3 Hz, 2H), 7.96 (dd, *J* = 6.5, 3.2 Hz, 2H), 7.65 (s, 2H), 7.49 (d, a stronger and a weaker peak group, *J* = 8.7 Hz, *J* = 8.6 Hz, 4H), 7.38 (dd, *J* = 5.8, 3.2 Hz, 2H), 7.34 (d, *J* = 8.6 Hz, 4H), 7.28 (d, 2H, *J* value can not be precisely calculated since the peak overlaps with the residual peak of $CDCl_3$), 7.06 and 7.04 (s, a stronger and a weaker peak group, 4H), 4.13–3.99 (m, 12H), 2.27–2.18 (m, 24H), 1.93–1.80 (m, 6H), 1.73–1.71 (m, 6H), 1.67–1.48 (m, 12H), 1.34–1.21 (m,

72H), 0.94 (t, 18H), 0.87 (d, 36H). ^{13}C NMR (101 MHz, $CDCl_3$, 298 K, Supplementary Figure 53) δ (ppm): 165.57, 153.36, 141.46, 135.26, 131.70, 131.60, 131.53, 131.37, 130.75, 130.21, 128.67, 128.54, 127.00, 125.29, 124.86, 124.63, 119.93, 105.82, 71.90, 67.84, 39.48, 39.39, 37.63, 37.47, 36.48, 32.06, 29.95, 29.83, 29.77, 28.12, 24.88, 22.85, 22.74, 19.71, 16.86, 16.68, 16.51, 14.27, 8.64. ^{31}P NMR (162 MHz, $CDCl_3$, 298 K, Supplementary Figure 54) δ (ppm): 12.24 (s, $^{1}J_{Pt\text{-}P}$ = 2379.78 Hz). MALDI-TOF (Supplementary Figure 55): *m*/*z* 2514.4160, $[M + H]^+$.

*Synthesis of compound **3***

The preparation procedures of **3** were similar to that of **2**, except that compound **6** (290 mg, 0.208 mmol) was utilized instead of **5**. The product was purified by silica gel column chromatography [eluent: petroleum ether/ethyl acetate = 6.5 : 1 (*v*/*v*)] to afford pure compound **3** as a dark green powder (172 mg, yield: 32.9%). ^{1}H NMR (400 MHz, $CDCl_3$, 298 K, Supplementary Figure 56) δ (ppm): 8.48 (dd, J = 6.9, 3.3 Hz, 2H), 7.66 (s, 2H), 7.49 (d, J = 8.6 Hz, 4H), 7.31 (d, J = 8.6 Hz, 4H), 7.23 (dd, J = 6.9, 3.2 Hz, 2H), 7.05 (s, 4H), 4.12–3.99 (m, 12H), 2.34–2.25 (m, 24H), 1.92–1.79 (m, 6H), 1.72–1.68 (m, 6H), 1.60–1.48 (m, 12H), 1.35–1.22 (m, 72H), 0.94 (t, 18H), 0.87 (d, 36H). ^{13}C NMR (101 MHz, $CDCl_3$, 298 K, Supplementary Figure 57) δ (ppm): 165.60, 160.06, 153.32, 141.41, 135.30, 135.12, 131.63, 130.17, 128.81, 125.63, 125.25, 119.96, 115.71, 114.20, 109.84, 108.38, 105.80, 71.88, 67.80, 39.48, 39.38, 37.62, 37.46, 37.44, 36.47, 32.05, 29.93, 29.82, 29.77, 28.11, 24.87, 24.85, 22.85, 22.74, 19.73, 19.69, 16.75, 16.58, 16.40, 14.27, 8.59. ^{31}P NMR (162 MHz, $CDCl_3$, 298 K, Supplementary Figure 58) δ (ppm): 12.11 (s, $^{1}J_{Pt\text{-}P}$ = 2360.34 Hz). MALDI-TOF (Supplementary Figure 59): *m*/*z* 2519.4641, $[M]^+$.

1. *Spectroscopy of **1**–**3** in the monomeric state*

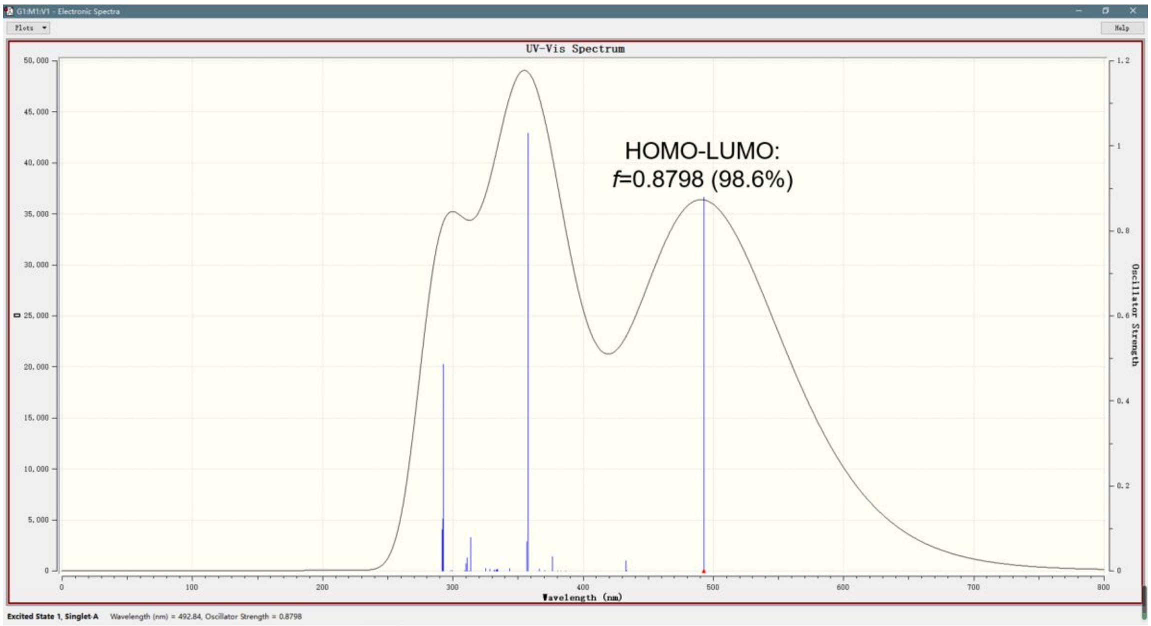

Supplementary Figure 3. Simulated electronic transition spectrum of **1** (without solvation model).

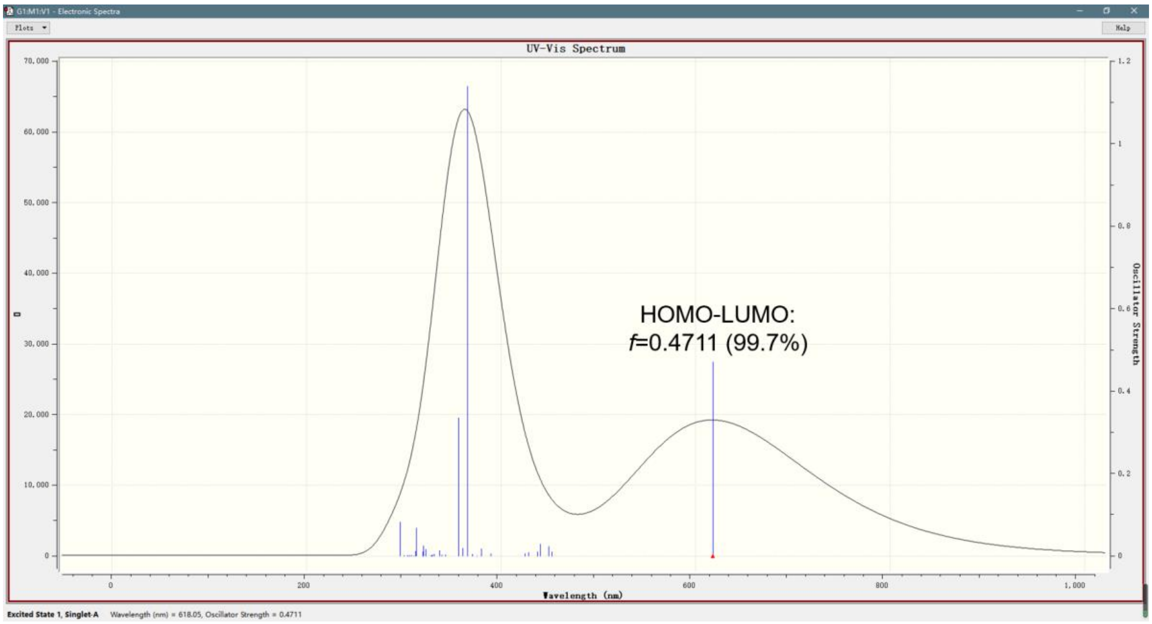

Supplementary Figure 4. Simulated electronic transition spectrum of **2** (without solvation model).

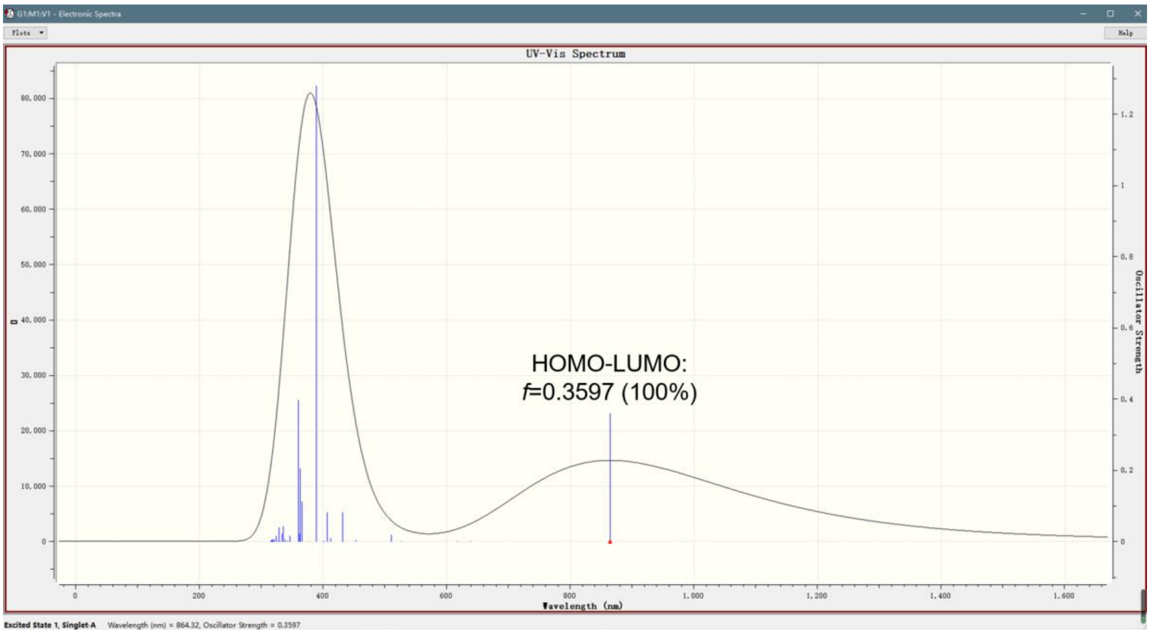

Supplementary Figure 5. Simulated electronic transition spectrum of **3** (without solvation model).

Supplementary Table 1. Electronic transitions of **1**–**3** calculated via TDDFT calculations

Compound	**1**	**2**	**3**
Excited state 1	492.84 nm, *f* = 0.8798<br>HOMO→LUMO (98.4%)	618.05 nm, *f* = 0.4711<br>HOMO→LUMO (99.7%)	864.32 nm, *f* = 0.3597<br>HOMO→LUMO (100%)
Excited state 2	357.73 nm, *f* = 1.0300<br>HOMO–2→LUMO+2 (37.1%)<br>HOMO–1→LUMO+1 (41.4%)<br>HOMO–1→LUMO+2 (18.9%)	365.88 nm, *f* = 1.1399<br>HOMO–5→LUMO (14.2%)<br>HOMO–2→LUMO+1 (6.30%)<br>HOMO–1→LUMO+2 (33.8%)<br>HOMO→LUMO+7 (41.0%)	431.89 nm, *f* = 0.0821<br>HOMO–6→LUMO (88.0%)<br>HOMO–4→LUMO (9.15%)
Excited state 3	356.62 nm, *f* = 0.0694<br>HOMO–2→LUMO+1 (48.2%)<br>HOMO–1→LUMO+1 (8.46%)<br>HOMO–1→LUMO+2 (41.5%)	356.70 nm, *f* = 0.3342<br>HOMO–5→LUMO (3.24%)<br>HOMO–2→LUMO+1 (83.7%)<br>HOMO→LUMO+7 (8.51%)	406.40 nm, *f* = 0.0820<br>HOMO→LUMO+2 (97.6%)
Excited state 4	313.87 nm, *f* = 0.0782<br>HOMO–6→LUMO (13.2%)<br>HOMO→LUMO+7 (66.7%)<br>HOMO→LUMO+8 (13.0%)<br>HOMO→LUMO+15 (2.68%)	313.18 nm, *f* = 0.0672<br>HOMO→LUMO+11 (86.1%)<br>HOMO→LUMO+12 (6.81%)	388.68 nm, *f* = 1.2799<br>HOMO–2→LUMO+1 (2.01%)<br>HOMO–1→LUMO+2 (2.48%)<br>HOMO→LUMO+3 (89.4%)
Excited state 5	292.52 nm, *f* = 0.4860<br>HOMO–4→LUMO+5 (17.8%)<br>HOMO–3→LUMO+6 (14.8%)<br>HOMO–2→LUMO+3 (5.56%)<br>HOMO–2→LUMO+4 (17.3%)<br>HOMO–1→LUMO+3 (4.60%)<br>HOMO–1→LUMO+4 (32.6%)	296.06 nm, *f* = 0.0825<br>HOMO–1→LUMO+4 (5.52%)<br>HOMO–1→LUMO+5 (69.4%)<br>HOMO–1→LUMO+6 (21.1%)	365.57 nm, *f* = 0.1113<br>HOMO–13→LUMO (4.22%)<br>HOMO–12→LUMO (8.60%)<br>HOMO–11→LUMO (20.1%)<br>HOMO–10→LUMO (16.9%)<br>HOMO–9→LUMO (39.0%)<br>HOMO–1→LUMO+2 (5.13%)
Excited state 6	292.18 nm, *f* = 0.1221<br>HOMO–4→LUMO+5 (11.0%)<br>HOMO–3→LUMO+6 (6.93%)<br>HOMO–2→LUMO+3 (19.8%)<br>HOMO–2→LUMO+4 (14.2%)<br>HOMO–1→LUMO+3 (14.9%)<br>HOMO–1→LUMO+4 (28.5%)		362.73 nm, *f* = 0.2046<br>HOMO–10→LUMO (4.97%)<br>HOMO–9→LUMO (12.0%)<br>HOMO–1→LUMO+2 (75.0%)
Excited state 7	291.87 nm, *f* = 0.0972<br>HOMO–4→LUMO+5 (18.9%)<br>HOMO–3→LUMO+6 (15.9%)<br>HOMO–2→LUMO+3 (29.4%)<br>HOMO–1→LUMO+3 (23.3%)<br>HOMO–1→LUMO+4 (3.01%)		359.39 nm, *f* = 0.3979<br>HOMO–2→LUMO+1 (93.5%)<br>HOMO→LUMO+3 (2.67%)

Supplementary Note 1: Electronic transitions with *f* values larger than 0.05 are listed.

*Absorption assignments of monomers **1−3** in UV region*: Compounds **1**−**3** display two absorption bands in the UV region: namely a low-energy broad band at 308~395 nm, together with a high-energy strong and sharp band. The detailed discussion of the spectroscopic properties is listed as follows.

The former bands range from 308 nm to 395 nm [λ_{max}: 332 nm (ε = 7.93 × 10^4 M^{-1} cm^{-1}) for **1**; 334 nm (ε = 6.76 × 10^4 M^{-1} cm^{-1}) for **2**; 328 nm (ε = 9.21 × 10^4 M^{-1} cm^{-1}) for **3**]. With reference to the previous literatures of *trans*-[Pt(PEt_3)$_2$(C≡CR)$_2$] complexes[18,19], they mainly arise from the intra-ligand (IL) [$\pi \rightarrow \pi^*$(C≡CR)] transitions, with minor contributions from the metal-to-ligand charge transfer (MLCT) [*d*(Pt)$\rightarrow \pi^*$(C≡CR)] transitions. TD-DFT calculations (Supplementary Figure 6) confirm that the IL transitions of amide ligands mainly contribute to the compositions. In addition, **3** exhibits an additional absorption at 386 nm (ε = 6.93 × 10^4 M^{-1} cm^{-1}). Based on the DFT calculation, it may be assigned to the high-energy transition of naphtho[2,3-*c*][1,2,5]selenadiazole unit perturbed by Pt(II) ions (Supplementary Figure 7a).

In terms of the latter bands, the maxima absorption signals locate at 282 nm (ε = 1.11 × 10^5 M^{-1} cm^{-1}) for **1**, 303 nm (ε = 1.18 × 10^5 M^{-1} cm^{-1}) for **2**, and 295 nm (ε = 1.01 × 10^5 M^{-1} cm^{-1}) for **3**. It mainly originates from the high-energy transitions of (hetero)acene units according to the previous literatures[5,6,17].

Electronic transitions based on TD-DFT calculations: We have employed TD-DFT calculations to understand the origin of UV-region transitions. For the simulated spectrum of **1**, the transition energy of excited state 2 (λ = 357.73 nm, f = 1.03) is close to experimental result (λ = 332 nm). TD-DFT calculations support that excited state 2 originates from HOMO–$m \rightarrow$LUMO+n ($1 \le m \le 2$, $1 \le n \le 2$) transitions. For HOMO–m orbitals ($1 \le m \le 2$), electronic densities are primarily delocalized on 4-alkynylaniline units of amide ligands (Supplementary Figure 6a), with minor contributions from Pt(II) ions. Upon excitation, the electron densities partially migrate to benzoyl units of amide ligands in LUMO+n orbitals (Supplementary Figure 6a), accompanied by the decreased electronic transitions of Pt(II) ions. Hence, these transitions primarily arise from IL transitions of amide ligands, with some admixture of MLCT transitions. Likewise, HOMO–$m \rightarrow$LUMO+n ($1 \le m \le 2$, $1 \le n \le 2$) transitions are also important compositions for **2** (excited state 2: λ = 365.88 nm, f = 1.1399; excited state 3: λ = 356.70 nm, f = 0.3342) and **3** (excited state 2: λ = 362.73 nm, f = 0.2046; excited state 3: λ = 359.39 nm, f = 0.3979) (Supplementary Figures 6b,c). Therefore, TD-DFT calculations support that **1**–**3** possess similar transition mechanisms for the absorptions at *ca*. 360 nm.

Notably, the simulated spectrum of **3** shows that another excited state (λ = 388.68 nm, f = 1.2799) could be generated within the range of 350~400 nm. The calculation result is consistent with the experimental spectroscopic data (λ_{max}: 386 nm). According to the TD-DFT calculations, the band primarily arises from HOMO→LUMO+3 transition (contribution: 89.4%). Since the electron densities of HOMO and LUMO+3 orbitals are delocalized on alkynylated naphtho[2,3-*c*][1,2,5]selenadiazole and Pt(II) ions (Supplementary Figure 7a), the band is assigned to the high-energy transition of naphtho[2,3-*c*][1,2,5]selenadiazole unit perturbed by Pt(II) ions according to TD-DFT results.

For the shorter wavelengths, the transition energies of **1**–**2** located at around 313 nm [Note: since **3** shows rather weak transitions ($f < 0.05$), we would not discuss transitions of **3** thereafter]. For **1**, the HOMO→LUMO+m ($7 \leq m \leq 8$) transitions are the primary compositions (contribution: 79.7%) for excited state 4 (λ = 313.87 nm, f = 0.078). Upon photo-excitation, the electron densities migrate from the alkynylated anthracene [with minor contribution from Pt(II) ions] to the amide ligands and $Pt(PEt_3)_2$ moieties (Supplementary Figure 7b). Therefore, ligand-to-ligand charge transfer (LLCT) and ligand-to-metal charge transfer (LMCT) transitions contribute to the excited state 4. In terms of **2**, LLCT/LMCT transitions from alkynylated tetracene to amide ligands and $Pt(PEt_3)_2$ units are also observed for HOMO→LUMO+m ($11 \leq m \leq 12$) transitions (λ = 313.18 nm for the excited state 4, f = 0.0672, Supplementary Figure 7c).

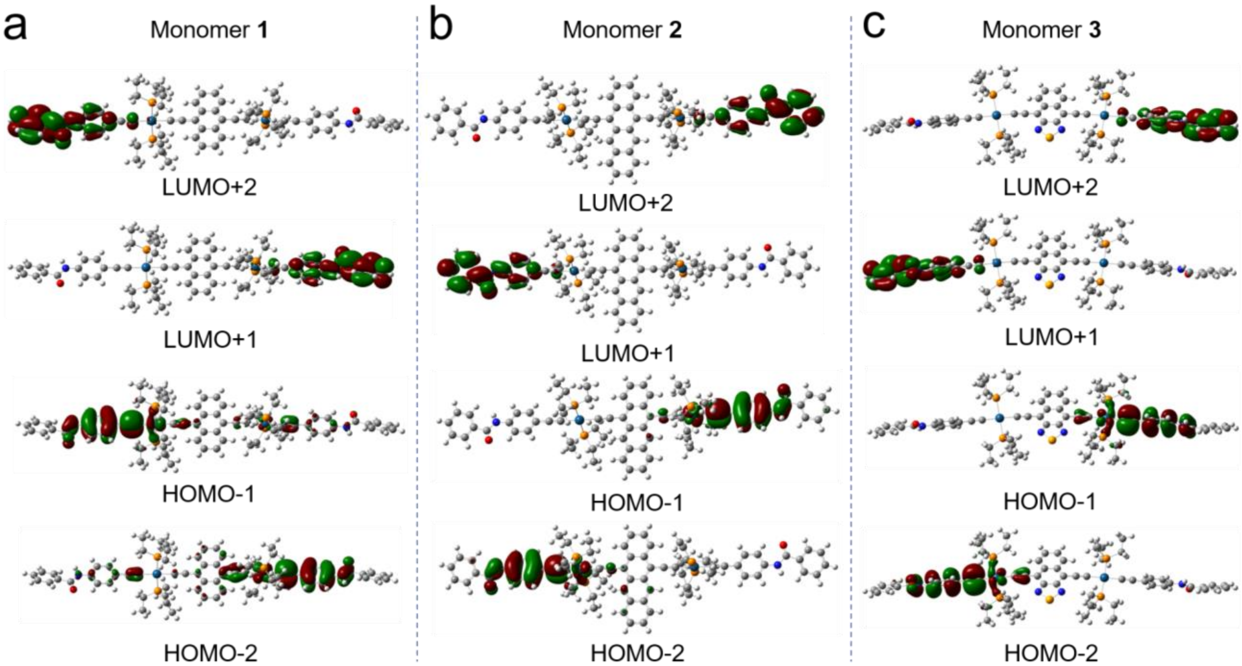

Supplementary Figure 6. Electron density distributions for HOMO–m ($1 \leq m \leq 2$) and LUMO+n ($1 \leq n \leq 2$) of a) **1**, b) **2**, and c) **3**.

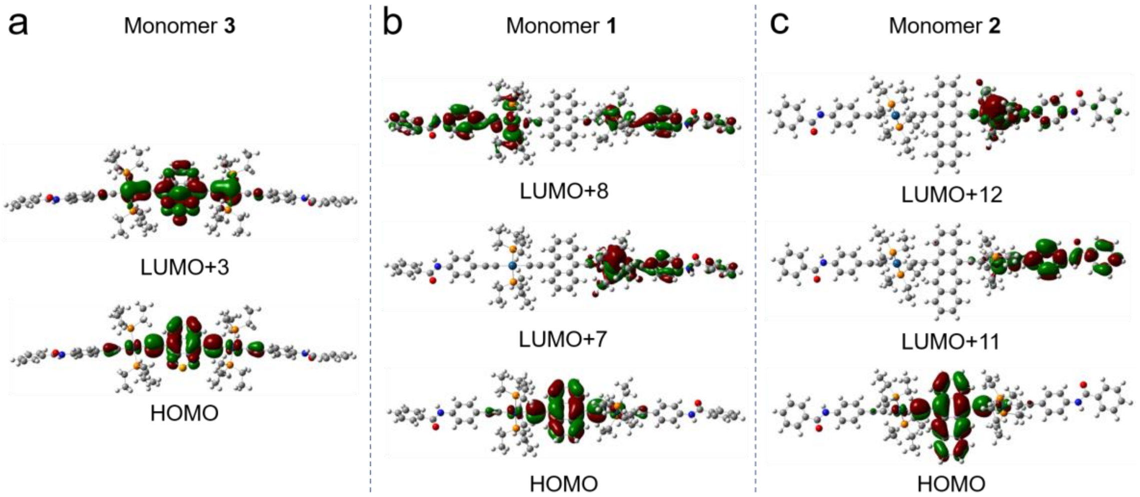

Supplementary Figure 7. Electron densities for a) HOMO and LUMO+3 orbitals of **3**, b) HOMO and LUMO–n ($7 \leq n \leq 8$) orbitals of **1**, and c) HOMO and LUMO–n ($11 \leq n \leq 12$) orbitals of **2**.

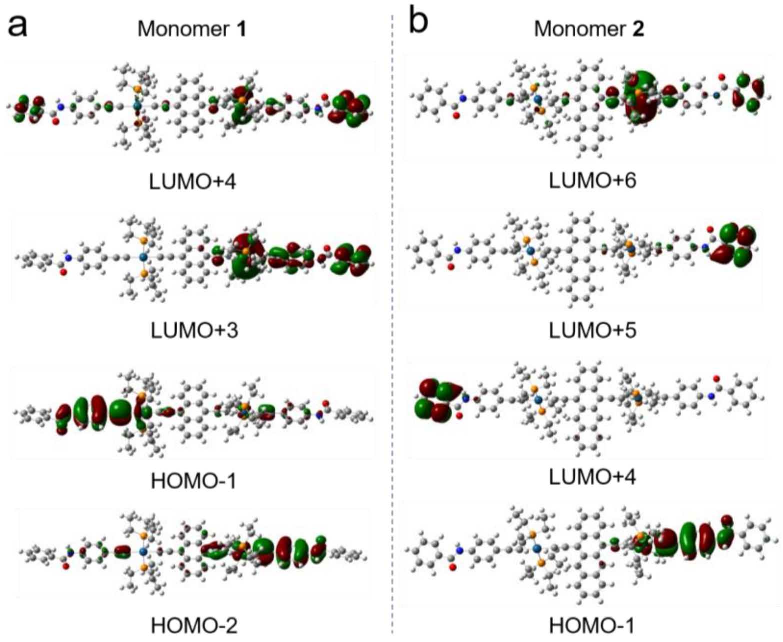

Supplementary Figure 8. Electron density distributions for a) HOMO–m ($1 \leq m \leq 2$) and LUMO+n ($3 \leq n \leq 4$) orbitals of **1**, b) HOMO and LUMO–n ($4 \leq n \leq 6$) orbitals of **2**.

Next, we turned to the highest transition regions in 290~300 nm (λ: 282 nm for **1** and 303 nm for **2**). For **1**, the HOMO–m→LUMO+n ($1 \leq m \leq 2$, $3 \leq n \leq 4$) transitions are mainly responsible for the formation of excited state 5 (λ = 292.52 nm, f = 0.4860). For LUMO+n ($3 \leq n \leq 4$) orbitals, the electronic densities are primarily delocalized on Pt(II) ions and benzoyl units of amide ligands (Supplementary Figure 8a). In particular, electron densities on the benzoyl moieties and Pt(II) ions are significantly higher than those of HOMO–m ($1 \leq m \leq 2$), whereas it is decreased for 4-alkynylaniline units of the amide ligands (Supplementary Figure 8a). Hence, electronic transitions with LMCT and IL characters contribute to the formation of excited state 5. For the structurally similar compound **2**, TD-DFT calculations also support that the electronic

transitions with LMCT and IL characters [HOMO–1→LUMO+m ($4 \leq m \leq 6$) transitions] contribute to the generation of excited state 5 (λ = 296.06 nm, f = 0.0825, Supplementary Figure 8b).

In summary, TD-DFT calculations are reliable methods to predict the absorption transitions of **1**–**3**. Some inconsistency exists for the higher energy UV-region transitions between the theoretical and experimental results. For the lower energy UV-region transitions, TD-DFT calculations unambiguously support the presence of IL/MLCT transitions of amide ligands and the metal-perturbed HOMO→LUMO transtions of (hetero)acene units, which are in accordance with the spectroscopic results.

*A discussion on whether or not the UV absorption bands exert impacts on the spectral red-shifiting of **1**–**3** in the visible/NIR region*: Generally, for the donor-π-acceptor organic molecules, electron communication exists between the donor (with relatively high transition energy) and acceptor (with relatively low transition energy) units via π-conjugated bridges, leading to the red-shifting of absorption bands upon excitation of the donor unit. However, the transition mechanisms of platinum(II) acetylide molecules reported in the current study are different with those of the traditional donor-π-acceptor molecules. When donor and acceptor units are linked via the platinum(II) ions, the 5*d* orbitals of Pt(II) and *p*-orbitals of the alkynylated ligands poorly overlap with each other. As a result, platinum(II) ions fail to act as the π-conjugated bridges. It restricts electronic communication and energy transfer between donor/acceptor units, as widely reported in the previous literatures[20–23]. Hence, in the current systems the peripheral amide ligands do not interfere with HOMO→LUMO transitions of the inner (hetero)acene units. Additionally, the high-energy transition bands of (hetero)acene units locate at UV region. Excitation of these bands results in the formation of high energy singlet excited states (S_n states), which undergo relaxation processes ($S_n \rightarrow S_1$) to the first singlet excited states (S_1 states). Hence, it exerts no impacts on the $S_0 \rightarrow S_1$ (corresponding to HOMO→LUMO) transitions of the (hetero)acene units.

With reference to the previously reported literatures[17,24,25], the spectral red-shifting of the HOMO→LUMO electronic transitions can be interpreted by metal-π interactions. For TIPS (triisopropylsilyl) substituted precursors **7**–**9** (Supplementary Figure 9a), the vibronic absorption bands red-shift from anthracene via tetracene to naphtho[2,3-*c*][1,2,5]selenadiazole derivatives. When TIPS substituents are replaced by $[Pt(PEt_3)_2X]^+$ moieties (X = alkynylated amide ligand, Supplementary Figure 9a), the HOMOs/LUMOs of alkynylated (hetero)acenes are perturbed by the molecular orbitals of $[Pt(PEt_3)_2X]^+$ moieties via metal-π interactions,

giving rise to the destabilization of the HOMOs/LUMOs of TIPS substituted precursors (Supplementary Figure 9b). Since the MOs of $[Pt(PEt_3)_2X]^+$ have better energy matching with the HOMOs of (hetero)acenes, they are destabilized to a large extent than the LUMOs, as revealed by the larger changes of HOMOs upon platination of (hetero)acene units (Supplementary Figure 9b). In particular, the destabilization effect endows HOMOs of **1**–**3** with the similar energy levels (E_{HOMO}: –4.129 eV for **1**, –4.042 eV for **2**, and –4.298 eV for **3**), while large fluctuations exist in the energy levels of LUMOs [E_{LUMO}: –1.342 eV for **1**, –1.772 eV for **2**, and –2.542 eV for **3**, Supplementary Figure 9b]. Therefore, the HOMO–LUMO energy gaps reduce from **1** (2.787 eV, 444.9 nm) via **2** (2.270 eV, 546.3 nm) to **3** (1.756 eV, 706.2 nm) (Supplementary Figure 9b). Overall, it can be concluded that the metal-π interactions play vital roles for the red-shifting of absorption bands in **1**–**3**.

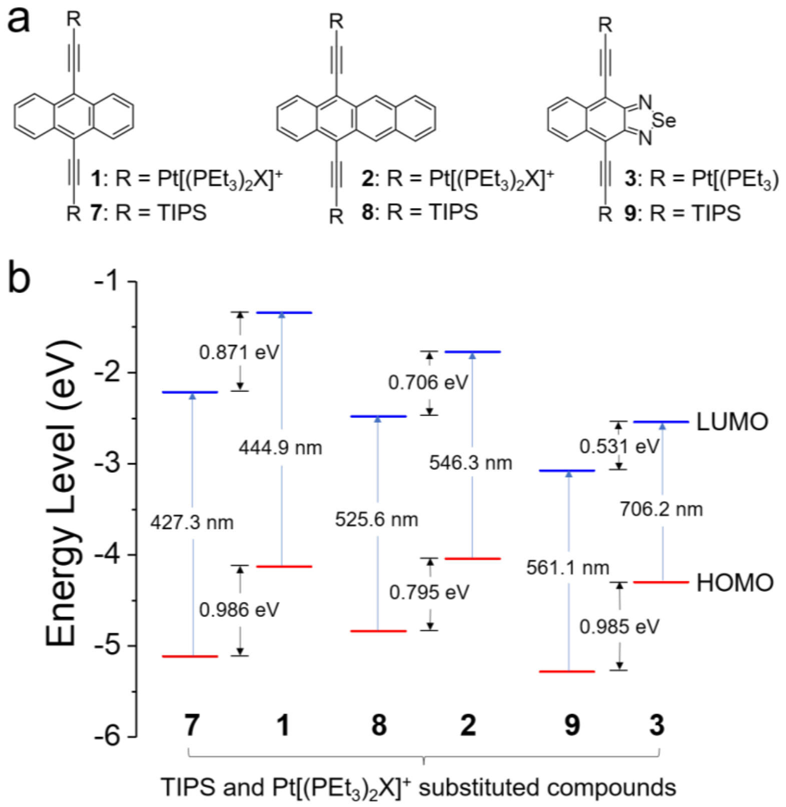

Supplementary Figure 9. a) Chemical structures for TIPS and $[Pt(PEt_3)_2X]$ substituted compounds bearing anthracene, tetracene, and naphtho[2,3-*c*][1,2,5]selenadiazole units. b) Energy level diagram for TIPS substituted precursors **7**–**9** [optimized at B3LYP/6-31G(d) level] and monomers **1**–**3**. The energy levels of HOMOs and LUMOs are marked by red and blue lines.

Supplementary Table 2. Spectroscopic properties for *σ*-platinated (hetero)acenes **1**–**3**

Monomer	$S_0 \rightarrow S_1$ transitions of vibronic band (nm, ε, 10^4 M^{-1} cm^{-1})	Maximal emission (nm)	Fluorescence quantum yield (%)
**1**	425 (1.53), 454 (3.49), 484 (4.66)	510[a]	48.3
**2**	505 (0.64), 539 (1.68), 5.81 (2.49)	609[a]	88.4
**3**	649 (1.78), 698 (2.02)	796[b]	4.11

[a] The data is measured in dilute dichloromethane. [b] The data is measured in 1,2-dichloroethane.

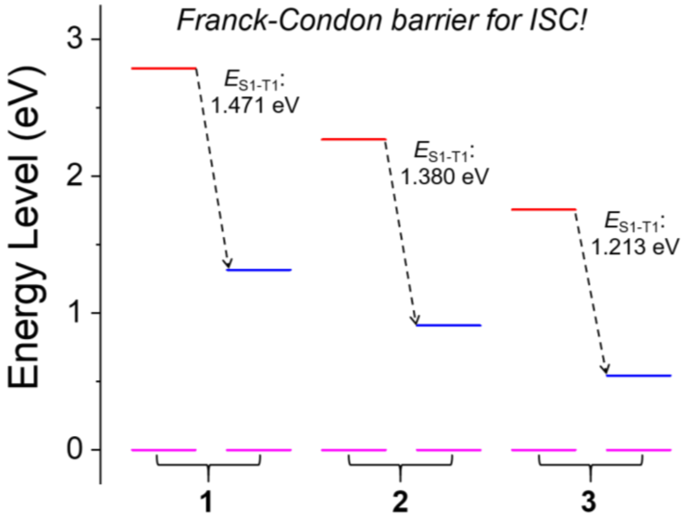

Supplementary Figure 10. Relative energies of calculated singlet and triplet excited states of **1**–**3** under Lanl2dz/6-31g(d) computational level. Notably, the S_1–T_1 energy gaps of **1**–**3** are determined to be 1.471 eV, 1.380 eV, and 1.213 eV, respectively. Hence, the large S_1–T_1 energy gaps lead to the Frank-Condon barriers, which are unfavorable for inter-system crossing.

*2. Supramolecular homopolymerization of **1**–**3***

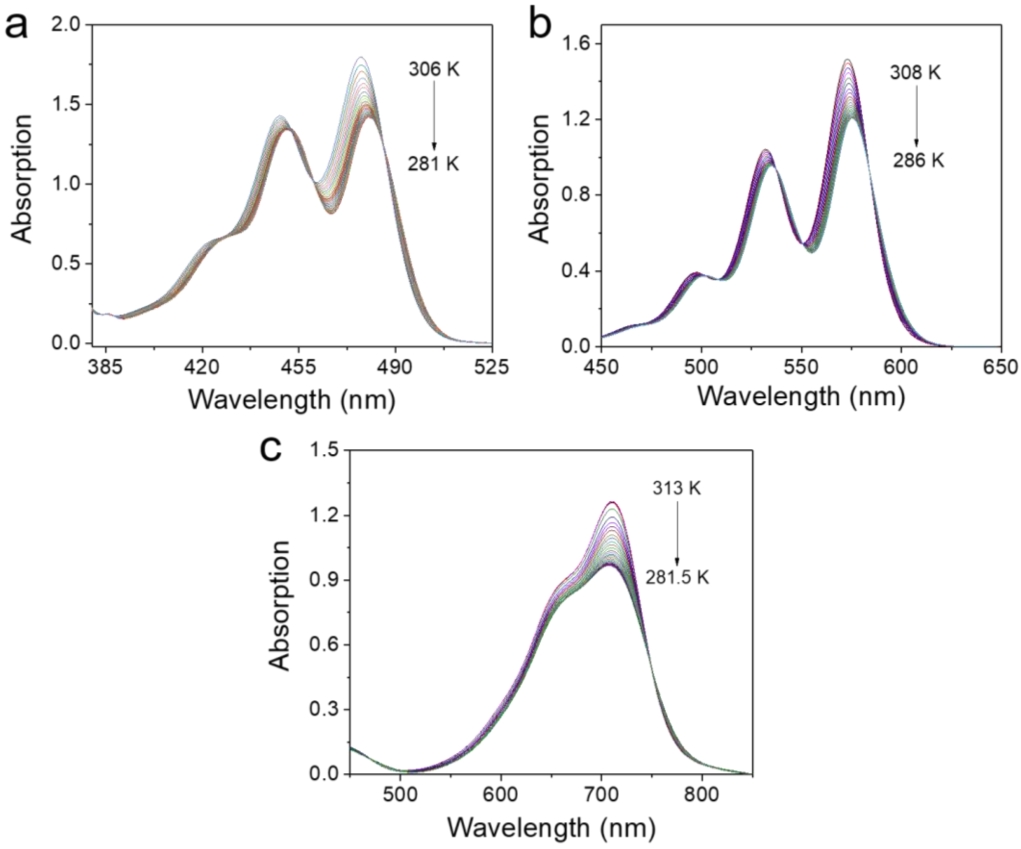

Supplementary Figure 11. Temperature-dependent absorption signal changes of a) **1**, b) **2**, and c) **3** (*c*: 5.0×10^{-5} mol L^{-1} in MCH). The presence of isobestic points reveals reversible conversion between monomeric and supramolecular homopolymeric states.

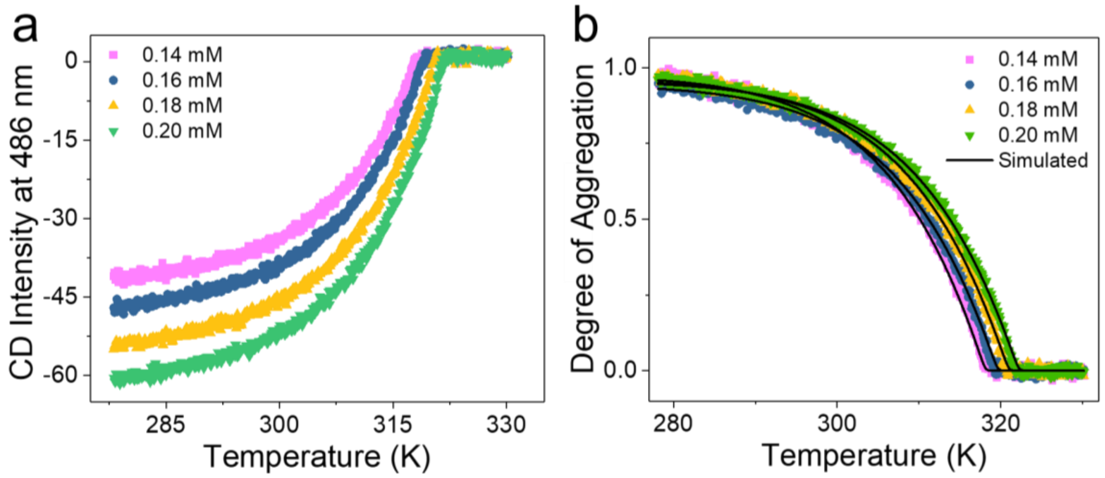

Supplementary Figure 12. a) Non-sigmoidal melting curves of **1** in MCH by tracking CD intensities at 486 nm. b) Non-linear fitting of melting curves via the mass balance models developed by Markvoort and ten Eikelder[10,11]. Based on the models, the enthalpy change (ΔH) and the entropy change (ΔS) for the supramolecular polymerization of **1** are –74.5 kJ mol^{-1} and –160 J mol^{-1} K^{-1}, respectively. The Gibbs free energy change (ΔG_e) at 298 K is determined to be –26.7 kJ mol^{-1}. Besides, the nucleation penalty for the supramolecular polymerization is –33.3 kJ mol^{-1}, corresponding to the cooperativity parameter of 1.5×10^{-6}. Hence, **1** adopts the cooperative nucleation-elongation mechanism during the supramolecular homopolymerization process.

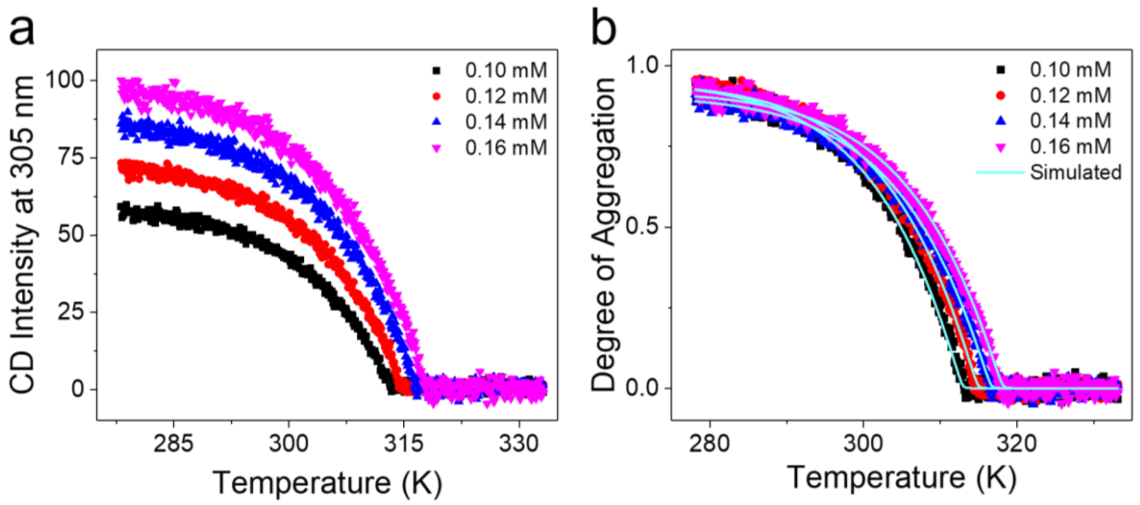

Supplementary Figure 13. a) Non-sigmoidal melting curves of **2** in MCH by tracking CD intensities at 305 nm. b) Non-linear fitting of melting curves the mass balance models developed by Markvoort and ten Eikelder. Based on the models, the enthalpy change (ΔH) and the entropy change (ΔS) for the supramolecular polymerization of **2** are –74.6 kJ mol^{-1} and –162 J mol^{-1} K^{-1}, respectively. The Gibbs free energy change (ΔG_e) at 298 K is determined to be –26.3 kJ mol^{-1}. Besdies, the nucleation penalty for the supramolecular polymerization is –30.5 kJ mol^{-1}, corresponding to the cooperativity parameter of 4.6 × 10^{-6}. Hence, **2** adopts the cooperative nucleation-elongation mechanism during the supramolecular homopolymerization process.

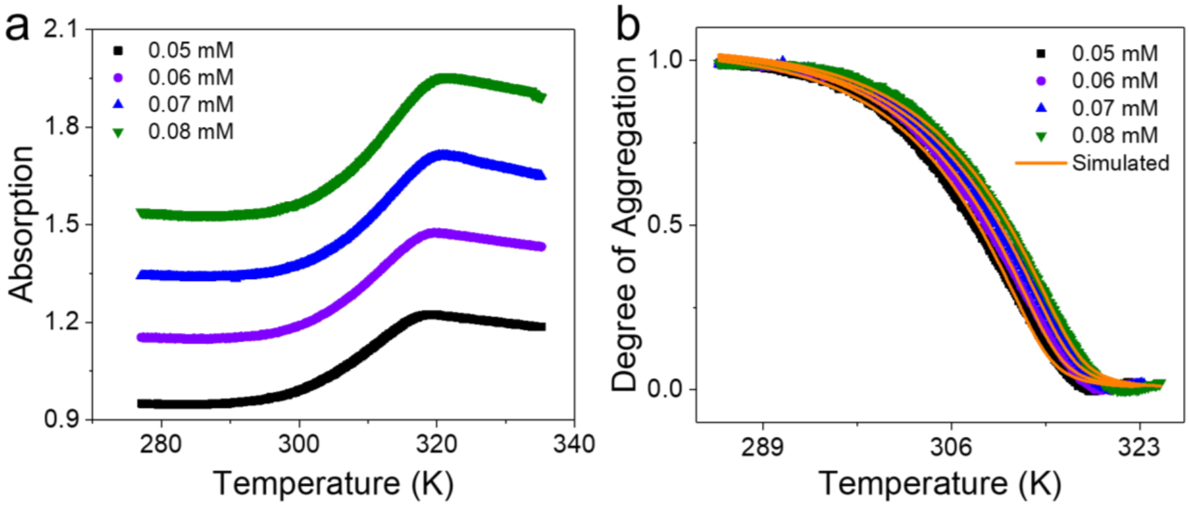

Supplementary Figure 14. a) Non-sigmoidal melting curves of **3** in MCH by monitoring the absorption intensity changes at 709 nm. b) Non-linear fitting of melting curves via the mass balance models developed by Markvoort and ten Eikelder. Based on the models, the enthalpy change (ΔH) and the entropy change (ΔS) for the supramolecular polymerization of **3** are –95.0 kJ mol^{-1} and –222 J mol^{-1} K^{-1}, respectively. The Gibbs free energy change (ΔG_e) at 298 K is determined to be –29.0 kJ mol^{-1}. Besdies, the nucleation penalty for the supramolecular polymerization is –13.5 kJ mol^{-1}, which corresponds to the cooperativity parameter of 4.3×10^{-3}. Hence, **3** adopts the cooperative nucleation-elongation mechanism during the supramolecular homopolymerization process.

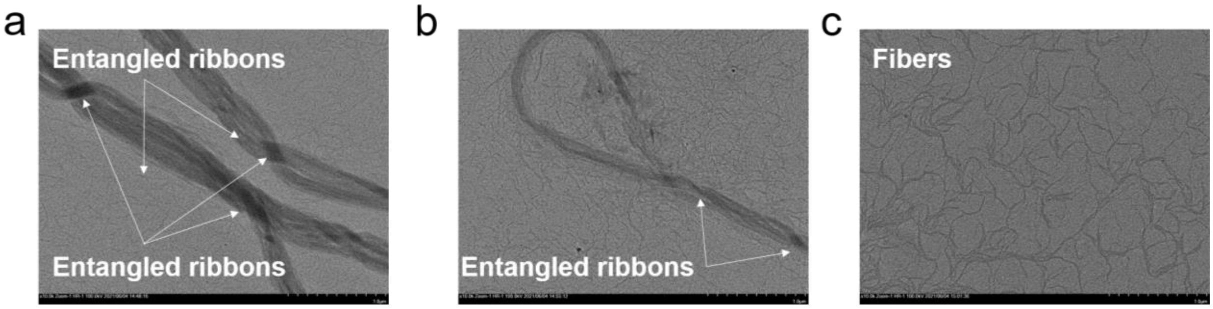

Supplementary Figure 15. TEM images for a) **1**, b) **2**, and c) **3** on copper grid (*c*: 8 × 10^{-5} mol L^{-1} in MCH). Nanostructures of supramolecular homopolymers derived from the individual compounds **1-3** are formed. For supramolecular homopolymers derived from **1** or **2**, entangled nanoribbons are also observed (entangled positions are marked by white arrows).

*A discussion on the different supramolecular homopolymerization behaviors of **3** from those of **1** and **2***: For the supramolecular homopolymerization behaviors of **3**, the cooperativity parameter is determined to be 4.29 × 10^{-3}, much larger than those of **1** and **2** (see Table 1 in the main text). The data reflect lower cooperativity for supramolecular homopolymerization process of **3**. Moreover, the ΔG_e value for the supramolecular polymerization process of **3** at 298 K is determined to be –29.0 kJ mol^{-1}, which is larger than those of **1**–**2** (ΔG_e: –26.7 kJ mol^{-1} for **1** and –26.3 kJ mol^{-1} for **2**, see Table 1 in the main text). As discussed in the main text, hydrogen bonding interactions play prominent role for the supramolecular homopolymerization processes of **1**–**3**. We rationalized that the additional non-covalent interactions may participate in the supramolecular polymerization process of **3**, because of the presence of the naphtho[2,3-*c*][1,2,5]selenadiazole unit in the inner core. DFT calculations show the larger dipole moment for the heteroacene monomer **3** than those of the acene monomers **1** and **2** (μ_D: 1.75 Debye of **3** versus 0.87 Debye for **1** and 1.20 Debye for **2**, Supplementary Figure 16). It potentially forms dipole-dipole interactions[26,27] between the neighboring molecules, thanks to the presence of intramolecular charge transfer effect. When both hydrogen bonding and dipole-dipole interactions serve as the non-covalent driving forces in the supramolecular polymerization process of **3**, their subtle interplay may affect the non-covalent interaction mode and thereby lead to the lower cooperativity for supramolecular polymerization[28,29].

The conclusion is validated by DFT calculations. For the homo-trimers $\mathbf{1}_3$–$\mathbf{2}_3$, the stacking directions of $\mathbf{1}_3$ and $\mathbf{2}_3$ exhibit slight deviation from the vertical direction (*z* axis, Supplementary Figure 16a,b). The phenomena indicate low slipped angles between the stacked monomers (slipped angles: 9.9° for $\mathbf{1}_3$ and 6.5° for $\mathbf{2}_3$, Supplementary Figure 16a,b). Meanwhile, the $Pt(PEt_3)_2$ moieties on the same side of monomers adopt the identical twisting directions, leading to molecular rotation between the neighboring monomers (Supplementary Figure 16a, b). By contrast, $\mathbf{3}_3$ adopt slipped conformations (slipped angles for the three possible conformers of $\mathbf{3}_3$

are 40.7°, 53.5°, and 48.9°, Supplementary Figure 16c), with no obvious rotation along the stacking directions (Supplementary Figure 16c). Hence, the contribution of dipole-dipole interactions may lead to the change of non-covalent stacking modes, which potentially result in the different cooperativity extent between **1**–**2** and **3** for the supramolecular homo-polymerization processes.

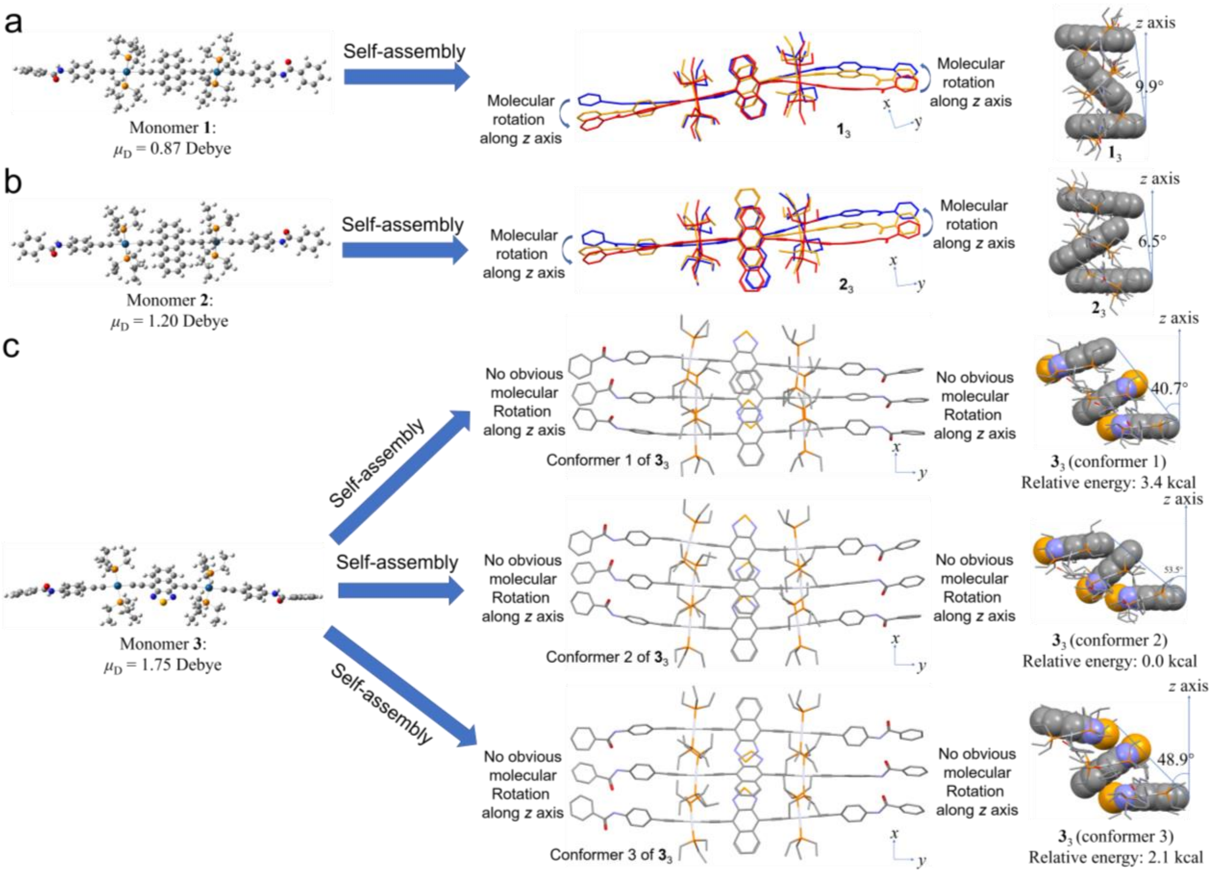

Supplementary Figure 16. Graphic representation for the supramolecular polymerization modes and calculated dipole moments of a) **1**, b) **2**, and c) **3**. All of the monomers and homo-trimers are optimized via DFT calculations. Depending on the spatial orientations of naphtho[2,3-*c*][1,2,5]selenadiazole units, **3** could possibly stack into three different conformers with minor differences in energies.

3. *Supramolecular copolymerization of **1/2** with excitation energy transfer*

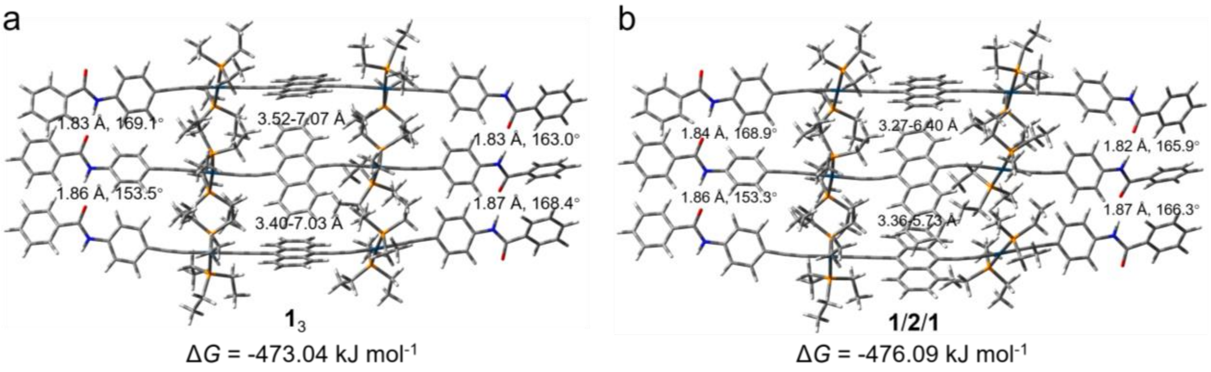

Supplementary Figure 17. a-b) Optimized geometries of $\mathbf{1}_3$ and **1**/**2**/**1**, together with the structural parameters and Gibbs free energy changes obtained via DFT calculations. Hydrogen bonds between the neighbouring amides are the main driving forces to assemble both $\mathbf{1}_3$ and **1**/**2**/**1**. π-stacking interactions between the acenes are rather weak due to the large interplanar distances. No obvious Δ*G* changes are observed for homo- and hetero-trimer.

In principle, two-component supramolecular copolymerization may give rise to the formation of self-sorted, random, or block copolymers[30]. In this regard, CD spectral characterization is a powerful tool to probe supramolecular copolymerization of **1**/**2**. With the gradual addition of **2** into the MCH solution of **1** (from 0 mol% to 20 mol%, by keeping the concentration of **1** at 8.0 × 10^{-5} mol L^{-1}), the CD signals of **1** at 486 nm decrease for their intensities (from –273.3 mdeg to –205.2 mdeg, Supplementary Figure 18a). Depending on CD melting curves, T_e values elevated with the increased amounts of **2** (from 314.1 K to 316.5 K, Supplementary Figure 18a and Supplementary Table 3). Meanwhile, the ΔG_e values are almost identical for **1** regardless of the amount of **2** (Supplementary Table 3), validating the heterogeneous elongation of either monomer to form randomly mixed copolymers **1**/**2**.

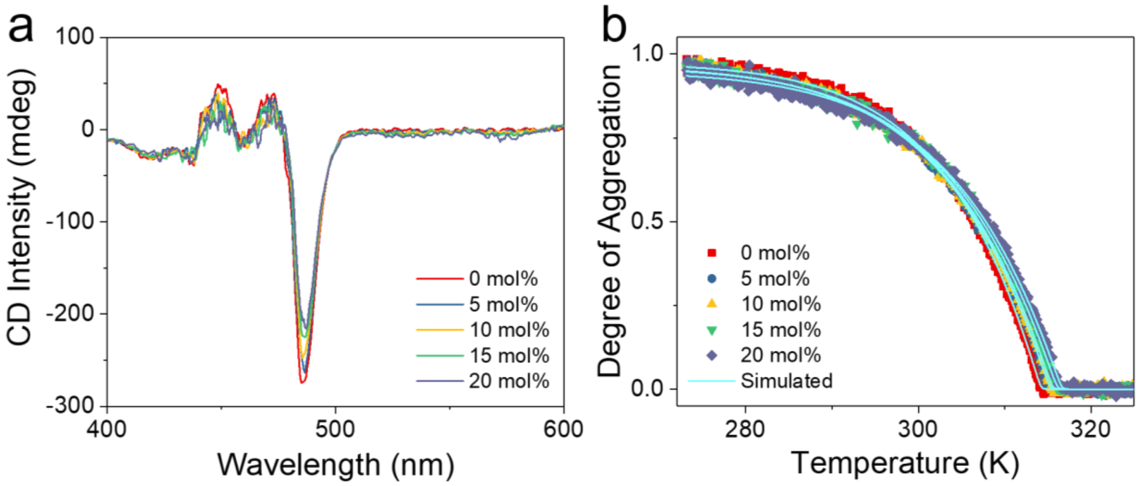

Supplementary Figure 18. a) CD spectra (1 cm cuvette, 298 K) of **1**/**2** (*c*: 8.0 × 10^{-5} mol L^{-1} for **1**) upon varying the ratio of **2** from 0 mol% to 20 mol%. b) Metling curves of **1**/**2** (*c*: 8.0 × 10^{-5} mol L^{-1} for **1**) obtained by monitroing the CD intensities at 486 nm. Non-linear fitting of melting curves via the mass balance models.

Supplementary Table 3. Thermodynamic parameters of supramolecular copolymers **1**/**2** upon varying the molar ratio of **2**.

Acceptor molar ratio	T_e (K)	ΔH_e (kJ mol^{-1})	ΔH_n (kJ mol^{-1})	ΔS (J mol^{-1} K^{-1})	ΔG_e (298 K, kJ mol^{-1})	ΔG_n (298 K, kJ mol^{-1})	σ (298 K)
0 mol%	314.1	–76.3	–43.0	–164	–27.3	5.9	1.5×10^{-6}
5 mol%	315.2	–69.9	–36.4	–143	–27.2	6.2	1.4×10^{-6}
10 mol%	315.4	–69.9	–37.5	–143	–27.2	5.1	2.1×10^{-6}
15 mol%	316.0	–68.9	–36.1	–140	–27.3	5.6	1.8×10^{-6}
20 mol%	316.5	–69.6	–36.4	–143	–27.4	6.2	1.5×10^{-6}

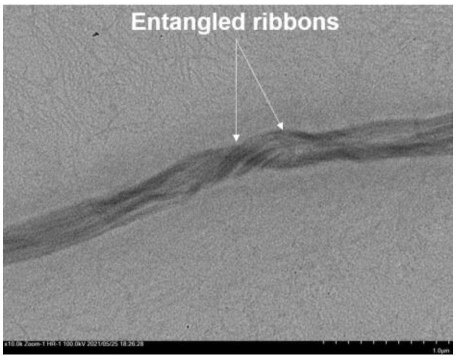

Supplementary Figure 19. TEM images of supramolecular copolymers **1**/**2** (c: 8 × 10^{-5} mol L^{-1} for **1**, 1.6 × 10^{-5} mol L^{-1} for **2** in MCH) on the cooper grid. The resulting supramolecular coplymers **1**/**2** tend to form entangled nanoribbons, similar to that of homopolymer of **1** (Supplementary Figure 15a).

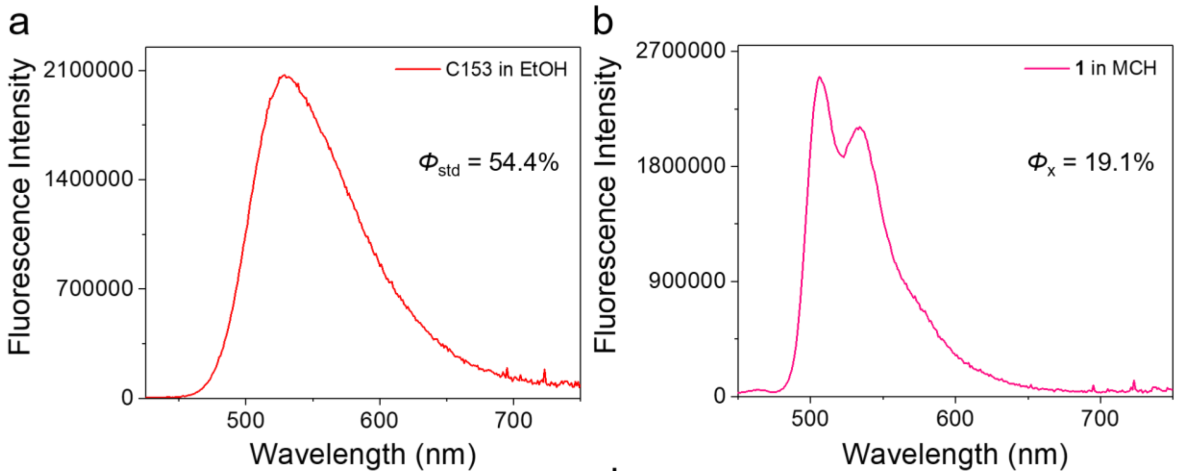

Supplementary Figure 20. a-b) Fluorescence emission spectra of the reference compound C153 (c: 1.0 × 10^{-5} mol L^{-1} in EtOH) and **1** (c: 8.0 × 10^{-5} mol L^{-1} in MCH). Both fluorescent emission spectra are excited at 391 nm.

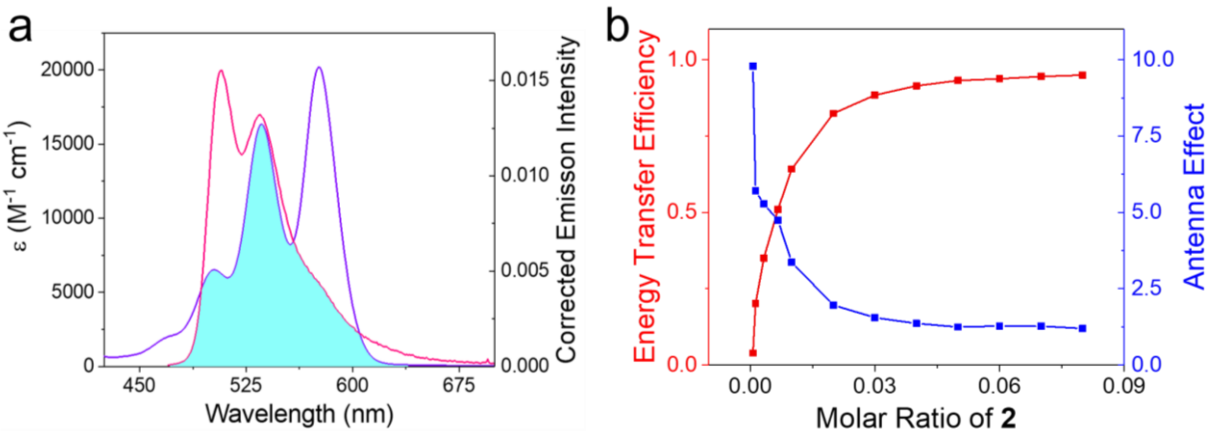

Supplementary Figure 21. a) Spectral overlap between **1** and **2**. b) Φ_{ET} and the antenna effect of supramolecular copolymers **1**/**2** (c: 8 × 10^{-5} mol L^{-1} for **1** in MCH) upon increasing the ratios of **2**.

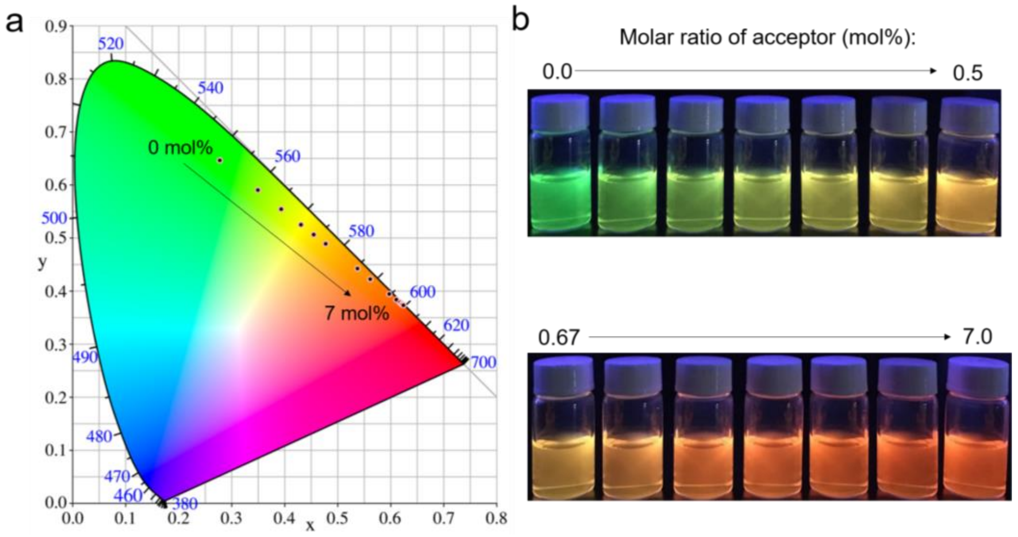

Supplementary Figure 22. a) CIE 1931 chromaticity coordinates of **1**/**2** (c: 8.0 × 10^{-5} mol L^{-1} for **1**) upon varying the molar ratio of **2**. b) Fluorescence images taken under 365 nm irradiation. As can be seen, green fluorescence emission of **1** is gradually quenched, accompanying with the emergence of organe fluorescence emission with the increasing molar ratio of **2**. Such phenomena support energy transfer from donor **1** to acceptor **2** in supramolecular copolymers **1**/**2**.

*A discussion on donor quenching mechanism of supramolecular copolymers **1**/**2***: Deeper insight into energy transfer mechanism is acquired by means of *Stern–Volmer* plot analysis. When comparing the steady-state fluorescence emission and the time-resolved fluorescence quenching behaviors (Supplementary Figure 23), it is evident that *Stern–Volmer* plots of steady-state fluorescence intensity (I_0/I) does not overlap with *Stern–Volmer* plots of donor fluorescence lifetimes (τ_0/τ). Such phenomena are characteristic for the involvement of both dynamic and static quenching mechanisms for fluorescence quenching of **1**[31]. In principle, the total fluorescence quenching efficiency (η_{total}) is the sum of dynamic quenching efficiency (η_{dyn}) and static quenching efficiency ($\eta_{tot} = \eta_{stat} + \eta_{dyn}$). At low acceptor loading (< 1 mol%), the

dynamic quenching plays a major role for fluorescent emission quenching. The conclusion is reflected by the significant contribution of η_{dyn} from 14.5% to 52.2% upon varying the ratio of **2** from 0.067–1 mol% (Supplementary Table 5). In the meantime, the η_{stat} values slightly change from 24.6% to 28.7% (Supplementary Table 5). By contrast, at higher acceptor concentration (> 1 mol%) static quenching plays a crucial role. It can be deduced by the significantly increase of η_{stat} values and the high energy transfer rates between D/A pairs (Supplementary Table 5). When the molar ratio of **2** varies from 1–10 mol%, k_{ET} rates increases substantially from 1.80 × 10^9 s^{-1} to 1.09 × 10^{10} s^{-1}, accompanied by the η_{stat} changes from 24.8% to 49.0%. Hence, higher acceptor concentration facilitates to the enhancement of k_{ET} rates, since the singlet excitons generated from **1** are easier to be trapped by acceptor **2**.

Besides, ground-state charge transfer between D/A pair is also possible to induce emission quenching of donors. It is excluded in supramolecular copolymers **1**/**2** via the experiments and DFT computation. When **1** and **2** are co-mixed in an equimolar ratio, no charge transfer band is appeared in the low-energy region of the absorption spectrum (Inset of Supplementary Figure 24a), indicating the absence of ground-state charge transfer. For DFT calculations, the optimized dimers **1**/**2** is employed to calculate the electronic transitions via TD-DFT method. Strong vibronic bands of **1** and **2** are dominately in the simulated spectrum (λ_{max}: 634.3 nm for **2** and 500.3 nm for **1**, Supplementary Figure 24a), thus attributing to the intramolecular metal-perturbed π-π* electronic transitions (Supplementary Figure 24b). Hence, intermolecular migration of electronic densities from **1** to **2** is not supported by TD-DFT calculations, suggesting the absence of ground-state charge transfer.

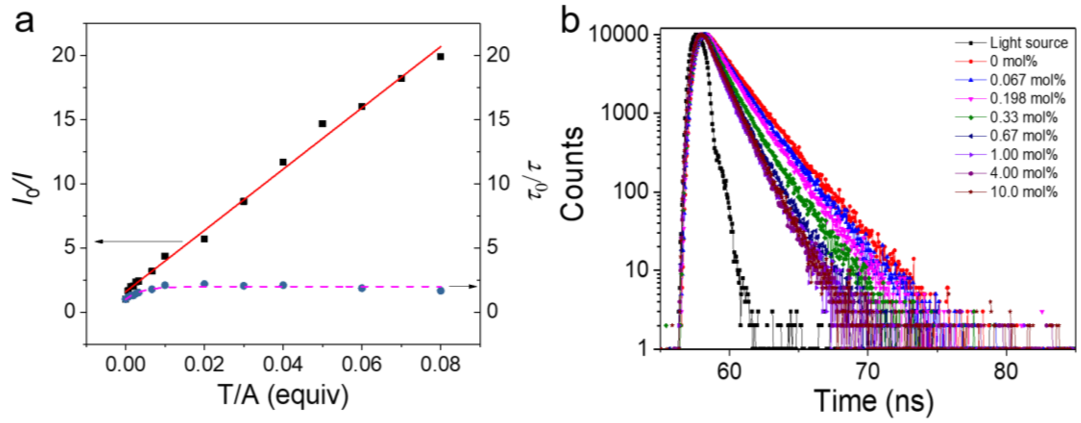

Supplementary Figure 23. a) *Stern–Volmer* plots of the steady-state fluorescence emission (I_0/I) and the time-resolved fluorescence lifetimes (τ_0/τ) of supramolecular copolymers **1**/**2** upon varying the molar ratios of **2**. According to the *Stern–Volmer* plots of I_0/I (red line), K_{sv} value of **1**/**2** is determined to be 2.98 × 10^6 M^{-1}. b) Fluorescent lifetime decay curves of **1** in **1**/**2** (*c*: 8.0 × 10^{-5} mol L^{-1} for **1**) at 507 nm. As can be seen, fluorescent lifetimes of **1** shorten upon increasing the molar ratios of **2**, indicating the excitation energy transfer from **1** to **2**.

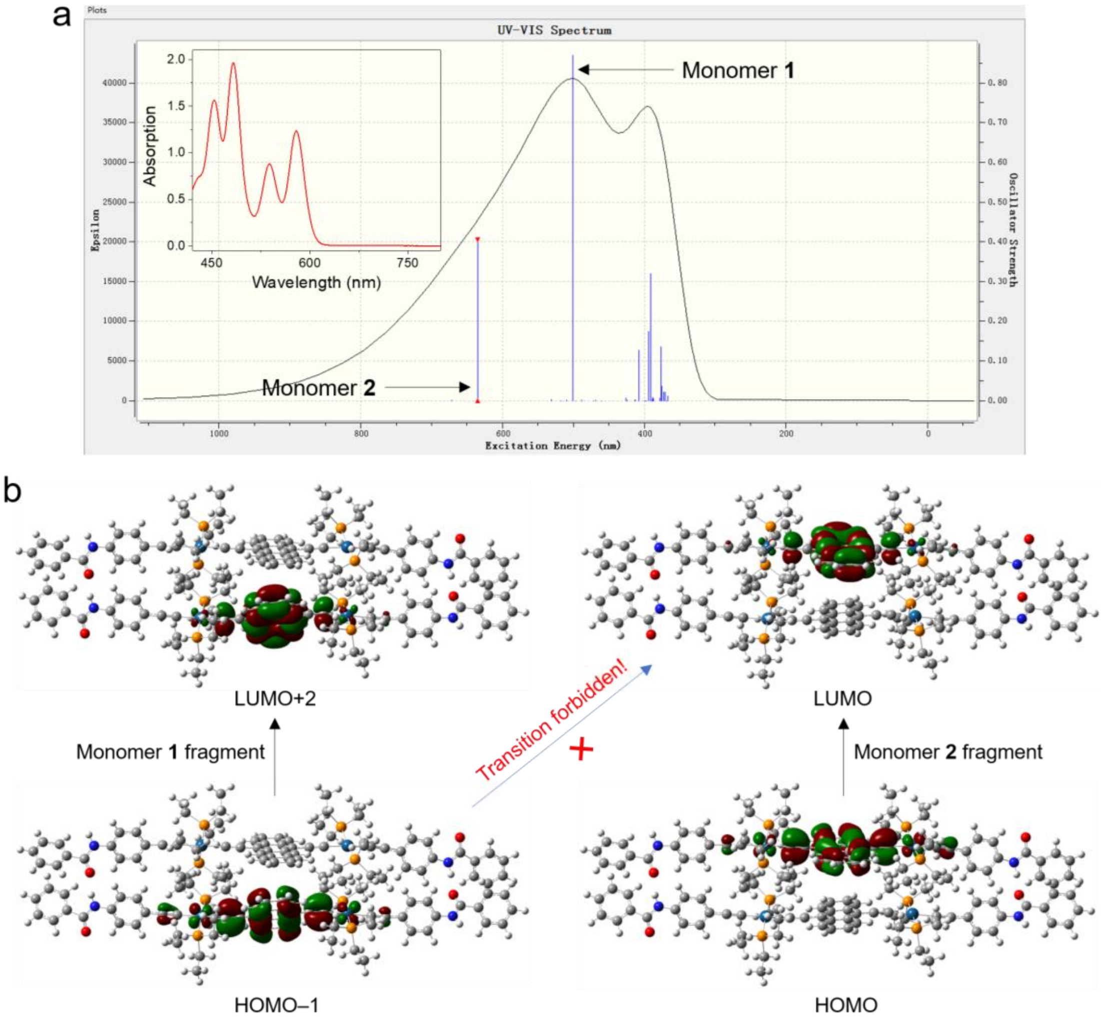

Supplementary Figure 24. a) Simulated absorption spectrum of the **1**/**2** dimer (Inset: absorption spectrum of **1**/**2** with an equimolar ratio in MCH). b) The corresponding electronic densities changes for the electronic transitions of acenes. For the optimized structure, the upper acene denotes tetracene unit, while the bottom is anthracene unit.

Supplementary Table 4. Fluorescence lifetime decays of **1**/**2** upon varying the molar ratios of **2**

Ratio (mol%)	τ_1 (ns)	α_1	τ_2 (ns)	α_2	τ_{avg} (ns)	χ^2
0	1.54	41.96%	2.10	58.31%	1.86	1.01
0.067	0.71	22.13%	1.85	77.87%	1.59	0.80
0.133	0.66	25.27%	1.70	74.73%	1.43	0.88
0.198	0.69	27.57%	1.75	72.43%	1.45	0.86
0.264	0.64	31.43%	1.60	68.57%	1.29	1.08
0.33	0.65	39.80%	1.61	60.20%	1.23	1.19
0.67	0.49	34.14%	1.35	65.86%	1.05	1.19
1.00	0.37	38.39%	1.21	61.61%	0.89	1.12
2.00	0.27	32.94%	1.13	67.06%	0.85	1.16
3.00	0.22	25.91%	1.16	74.09%	0.91	1.15
4.00	0.22	27.93%	1.15	72.07%	0.89	1.04
6.00	0.27	18.74%	1.17	81.26%	1.00	1.19
10.0	0.26	18.12%	1.16	81.88%	0.99	1.19

Supplementary Table 5. Dynamic and static quenching of **1**/**2** upon varying the molar ratios of **2**

Ratio (mol%)	k_{ET} (s^{-1})	η_{tot}	η_{dyn}	η_{stat}
0	0	0%	0%	0%
0.067	3.52×10^8	39.6%	14.5%	25.1%
0.133	5.23×10^8	49.3%	23.1%	26.2%
0.198	5.53×10^8	50.7%	22.0%	28.7%
0.264	7.30×10^8	57.6%	30.6%	27.0%
0.33	7.58×10^8	58.5%	33.9%	24.6%
0.67	1.17×10^9	68.5%	43.5%	25.0%
1.00	1.80×10^9	77.0%	52.2%	24.8%
2.00	2.52×10^9	82.4%	54.3%	28.1%
3.00	4.10×10^9	88.4%	51.1%	37.3%
4.00	5.71×10^9	91.4%	52.2%	39.2%
6.00	8.13×10^9	93.8%	46.2%	46.6%
10.0	1.09×10^{10}	95.3%	46.3%	49.0%

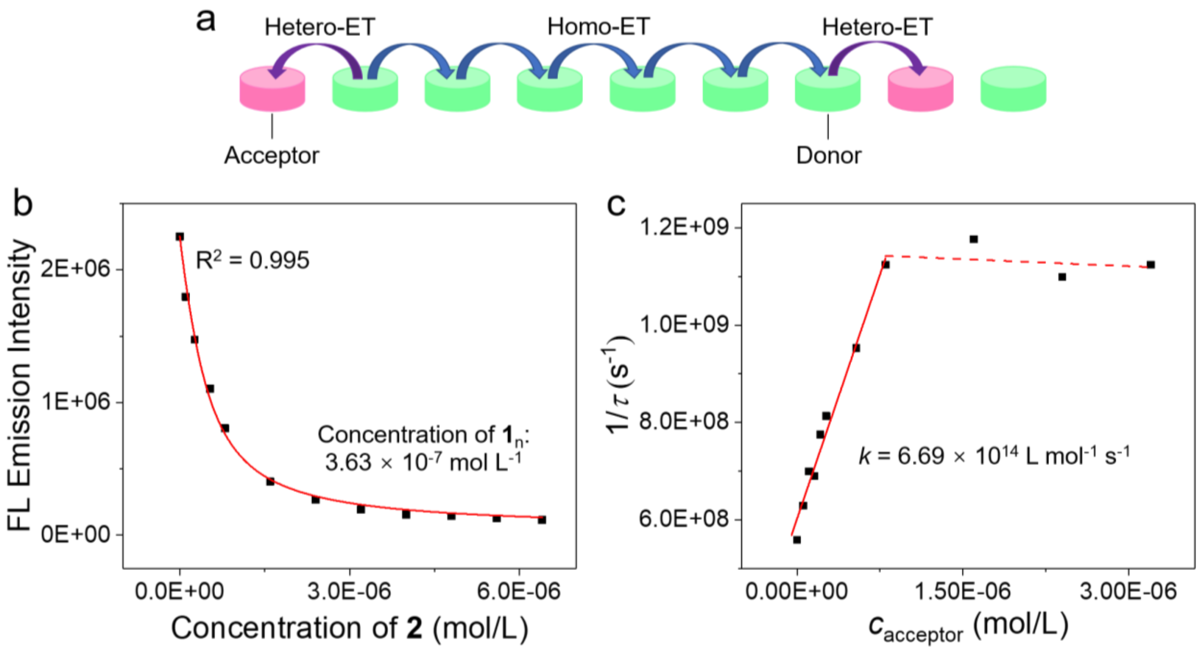

Supplementary Figure 25. a) Graphic represenation for homo- and hetero- energy transfer in supramolecular copolymers **1**/**2**. b) Non-linear fitting of the donor **1** emission intensities versus the acceptor **2** concentration. *n* is the ratio between the concentration of **1** and the concentration of donor matrix $\mathbf{1}_n$. c) Plots of the reciprocal of emission lifetimes of **1** versus the concentration of **2**. It varies linearly upon increasing the concentration of **2** to 8.00×10^{-7} mol L^{-1}.

4. Supramolecular copolymerization of ***2/3*** *with excitation energy transfer*

CD spectroscopy is employed to probe supramolecular copolymerization between **2** and **3**. With the gradual addition of **3** into **2** (from 0 mol% to 10 mol%, by keeping the concentration of **2** at 8.0 × 10^{-5} mol L^{-1}), the CD signals of **2** slightly decrease for their intensities (Supplementary Figure 26a). Depending on the CD melting curves, T_e values slightly elevated with the increased ratios of **3** (from 315.2 K to 315.6 K, Supplementary Figure 26b). Meanwhile, the ΔH values increase from –79.7 kJ mol^{-1} to –93.0 kJ mol^{-1}. It is consistent with the supramolecular homopolymerization processes, in which the ΔH value of **3** is higher than that of **2**. Nevertheless, the ΔG_e values are almost identical regardless of the amount of **3**, because of the increase of ΔS values with the higher loading of **3**. Overall, all of these phenomena exclude the self-sorting arrangement between **2** and **3**, and thereby support their supramolecular copolymerization tendency.

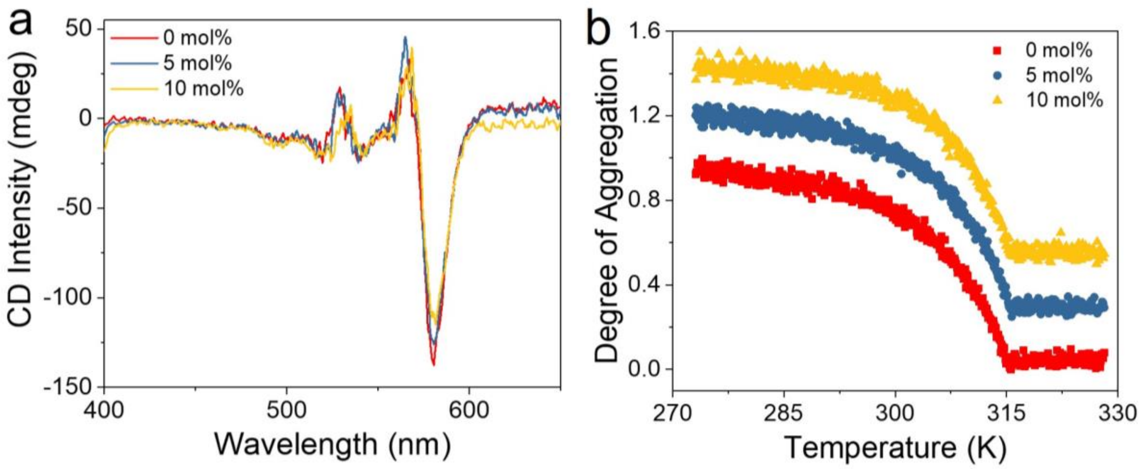

Supplementary Figure 26. a) CD changes of supramolecular copolymers **2**/**3** upon varying the molar ratio of **3**. The concentration of **2** is kept at 8.0 × 10^{-5} mol L^{-1}. b) Nonlinear fitting for melting curves of **2**/**3** upon varying the ratio of **3**. In panels the curves are shown with a 0.2 offset. Non-linear fitting of melting curves is performed via the mass balance models (Supplementary Table 6).

Supplementary Table 6. Thermodynamic parameters of **2**/**3** upon varying the molar ratio of **3**

Molar ratio of **3**	T_e (K)	ΔH_e (kJ mol^{-1})	ΔH_n (kJ mol^{-1})	ΔS (J mol^{-1} K^{-1})	ΔG_e (298 K) (kJ mol^{-1})	ΔG_n (298 K) (kJ mol^{-1})	σ (298 K)
0 mol%	315.2	–79.7	–47.9	–174	–27.7	4.0	2.7×10^{-6}
5 mol%	315.7	–83.5	–52.6	–186	–28.1	2.8	3.9×10^{-6}
10 mol%	315.6	–93.0	–62.3	–216	–28.6	2.1	4.2×10^{-6}

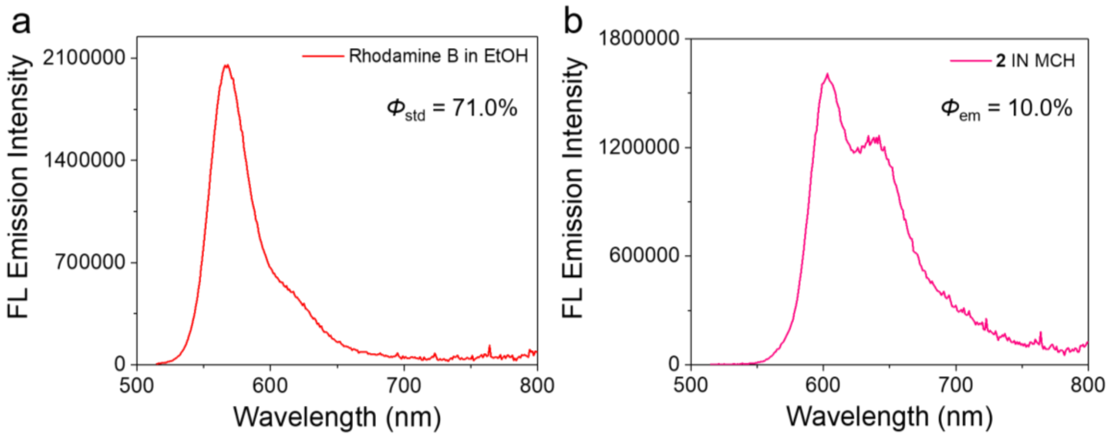

Supplementary Figure 27. a-b) Fluorescent emission spectra of the reference compound, rhodamine B (c: 1.0 × 10^{-5} mol L^{-1} in EtOH) and **2** (c: 8.0 × 10^{-5} mol L^{-1} in MCH). Both fluorescent spectra are excited at 495 nm. The relative quantum yield for **2** is 10.0%.

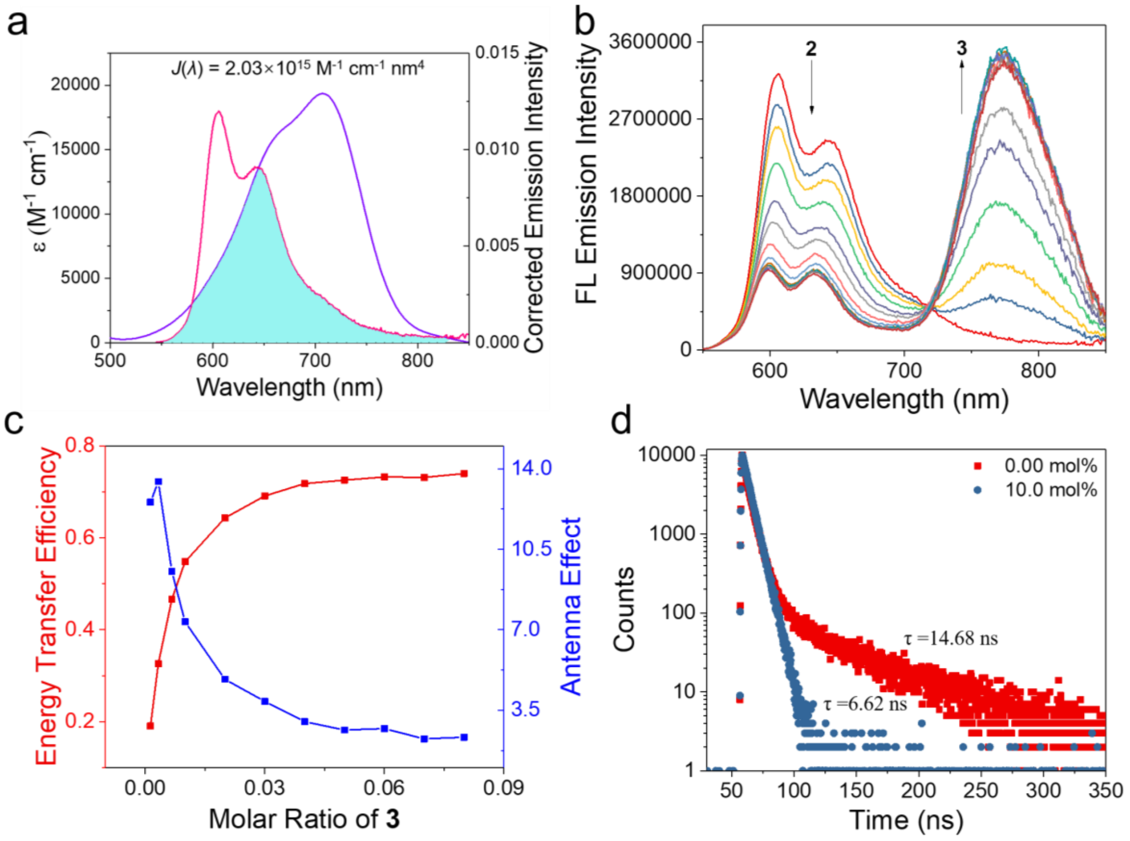

Supplementary Figure 28. a) Spectral overlap between the emission of donor **2** and absorption of acceptor **3**. b) Steady-state fluorescent emission changes upon varying molar ratios of **3** from 0 mol% to 8 mol% (c: 8.0 × 10^{-5} mol L^{-1} for **2** in MCH). c) Φ_{ET} and the antenna effect of **2**/**3** upon increasing the concentrations of **3**. The NIR emission band of **3** is enhanced by indirect donor excitation at 525 nm rather than direct excitation at 700 nm (antenna effect > 2.25). d) Fluorescence lifetime decay curves for **2** and **2**/**3** at 600 nm.

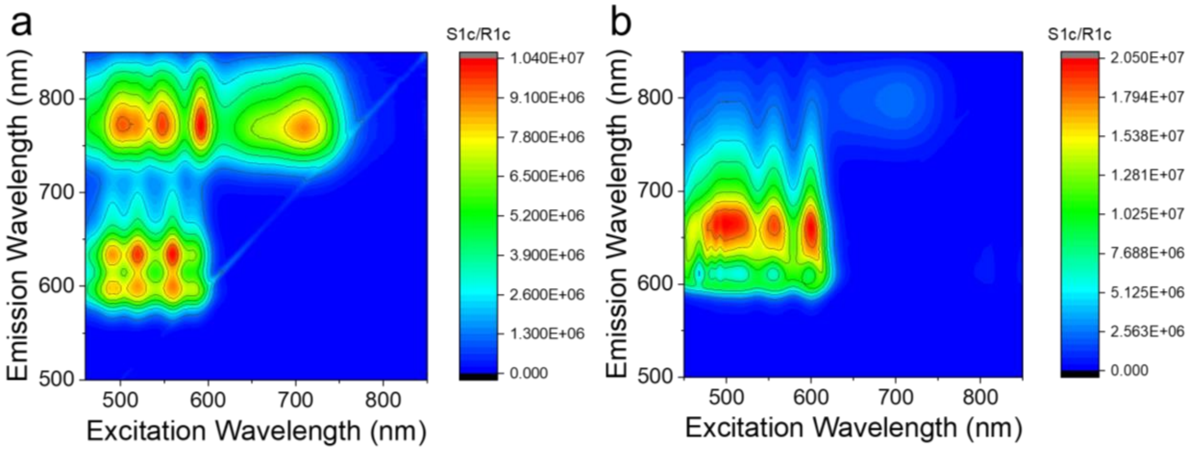

Supplementary Figure 29. Excitation spectra of the binary species **2**/**3** (c: 8.0 × 10^{-5} mol L^{-1} for **2** and 8.0 × 10^{-6} mol L^{-1} for **3**) in a) MCH and b) $CHCl_3$. In MCH, NIR emission band of **3** appears when exciting the vibronic absorption bands of **2**, suggesting the presence of energy transfer. In comparison, no NIR emission is observed for the the binary species **2**/**3** in $CHCl_3$. The results prove that supramolecular copolymerization is necessary for efficient energy transfer.

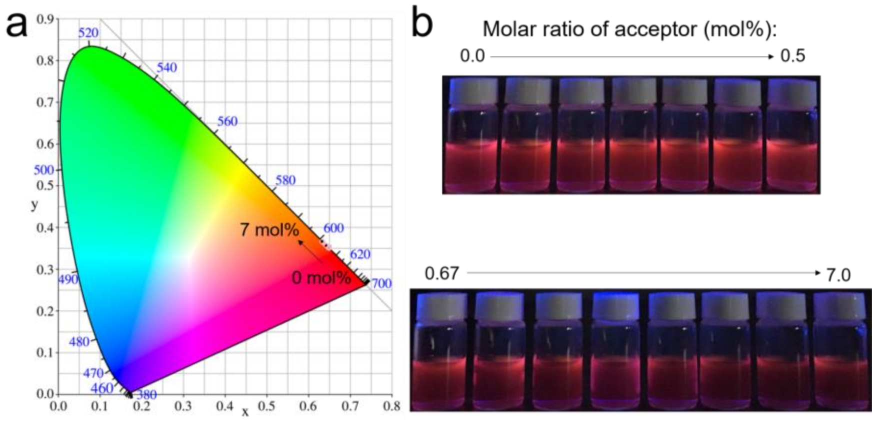

Supplementary Figure 30. a) CIE 1931 chromaticity coordinates of supramolecular copolymers **2**/**3** (c: 8.0 × 10^{-5} mol L^{-1} for **2** in MCH) upon varying the molar ratio of **3**. b) Fluorescence images taken under 365 nm irradiation. As can be seen, red fluorescence emission of **2** is gradually weakened with the increasing amount of **3**.

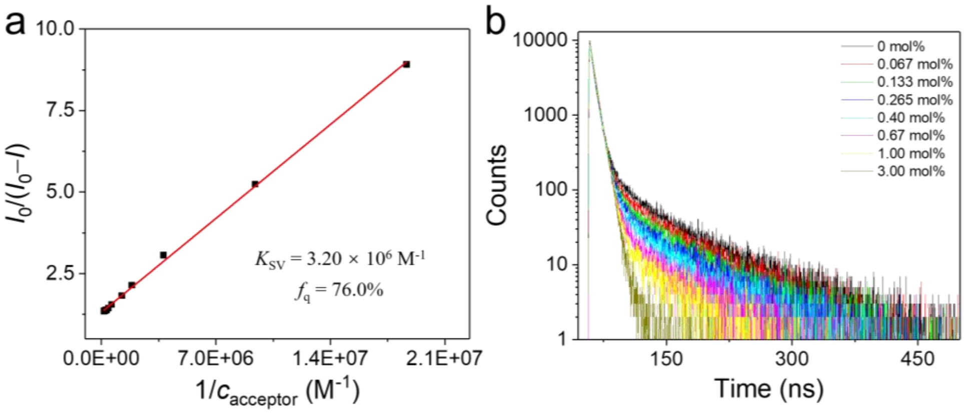

Supplementary Figure 31. a) Modified *Stern–Volmer* plot for fluorescence quenching of **2** in the presence of acceptor **3**. Through linear plotting of $I_0/(I_0 - I)$ versus the reciprocal of the concentration of acceptor, we can obtain K_{SV} = 3.20 × 10^6 M^{-1} and f_q = 76.0%. The f_q value confirms that 76.0% of excitons are trapped by acceptors. With reference to the previous literature[32], we rationalize that

exciton radiative relaxation contributes to the incomplete quenching of donors, since excitons may be not trapped by acceptors. b) Fluorescent lifetime decay curves of **2** in **2**/**3** (c: 8.0 × 10^{-5} mol L^{-1} for **2** in MCH) at 600 nm. The lifetime shortens upon increasing the molar ratio of **3**, indicating the excitation energy transfer from **2** to **3**.

Supplementary Table 7. Fluorescence lifetime decays of supramolecular copolymers **2**/**3** upon varying the molar ratios of **3**.

Ratio (mol%)	τ_1 (ns)	α_1	τ_2 (ns)	α_2	τ_3 (ns)	α_3	τ_{avg} (ns)	χ^2
0	2.13	17.71%	6.46	66.67%	64.0	15.62%	14.68	1.01
0.067	2.58	18.76%	6.60	69.23%	62.5	12.01%	12.56	0.95
0.133	1.97	13.05%	6.32	77.51%	59.0	9.45%	10.73	0.87
0.199	1.91	12.69%	6.30	78.94%	56.3	8.37%	9.93	0.84
0.265			5.60	91.25%	50.6	8.75%	9.54	1.17
0.330			5.63	92.26%	47.4	7.74%	8.86	1.19
0.400	1.37	13.23%	6.06	79.71%	48.9	7.05%	8.48	0.91
0.467			5.66	93.18%	44.3	6.82%	8.30	1.13
0.533	1.39	13.04%	6.09	81.59%	44.3	5.27%	7.48	1.12
0.670			5.73	95.30%	39.3	4.70%	7.31	1.11
0.800	1.12	9.43%	6.11	86.91%	41.4	3.66%	6.93	0.90
1.00			5.71	96.22%	29.6	3.78%	6.61	1.03
2.00			6.04	100%			6.04	1.13
3.00			6.07	100%			6.07	1.12

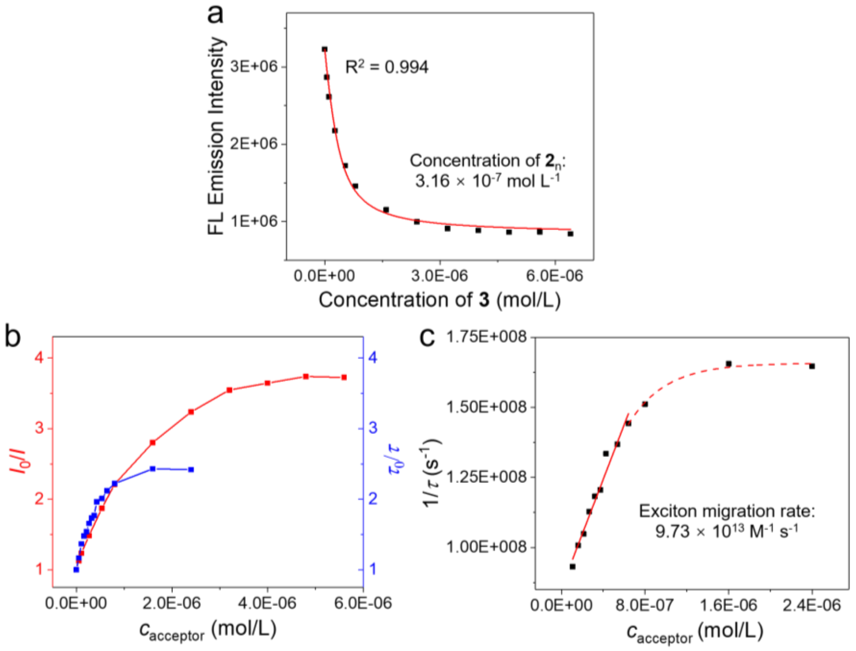

Supplementary Figure 32. a) Non-linear fitting of the emission intensities of **2** versus the concentration of **3**. b) *Stern–Volmer* plots of steady-state fluorescence emission (I_0/I) and time-resolved fluorescence quenching (τ_0/τ) of **2**/**3**. *Stern–Volmer* plots of I_0/I and τ_0/τ are nearly equivalent when the ratio of **3** is lower than 1 mol%, confirming that the quenching of **2** is mainly contributed by dynamic quenching. c) Plots of the reciprocal of fluorescence lifetime ($1/\tau$) versus the concentration of **3** in **2**/**3**. The concentration of **2** is kept at 8.0 × 10^{-5} mol L^{-1} in MCH. The fluorescence lifetimes of **2** are obtained by monitoring the emission at 600 nm.

5. *Sequential energy transfer behaviors of supramolecular copolymers* ***1/2/3***

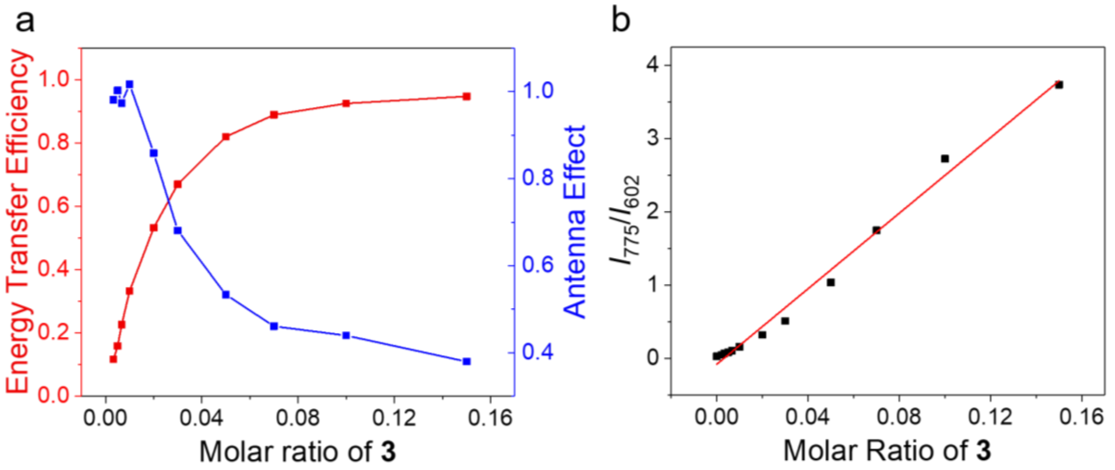

Supplmentary Figure 33. a) Φ_{ET} value of **2** and the antenna effect of **3** upon increasing the molar ratios of **3** into supramolecular copolymers **1**/**2** (The concentrations of **1** and **2** is 8.0 × 10^{-5} mol L^{-1} and 1.6 × 10^{-5} mol L^{-1} in MCH). b) Ratiometric plot of supramolecular copolymers **1**/**2**/**3** upon increasing the ratio of **3**. When the molar ratio of **3** increases from 0 mol% to 15 mol%, it varies linearly with the gradual addition of **3**. The phenomenon suggests the dispersion of **3** into the supramolecular copolymeric assemblies of **1**/**2**..

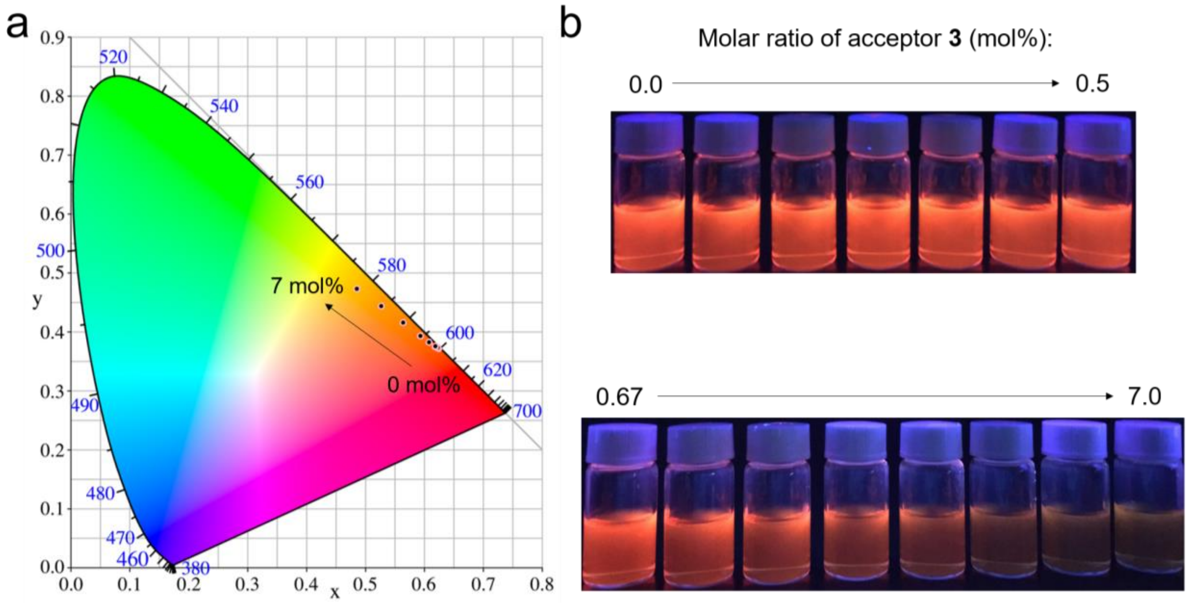

Supplementary Figure 34. a) CIE 1931 chromaticity coordinates of **1**/**2**/**3** (*c*: 8.0 × 10^{-5} mol L^{-1} for **1** and 1.6 × 10^{-5} mol L^{-1} for **2** in MCH) upon varying the molar ratios of **3**. b) The corresponding fluorescent images taken under 365 nm irradiation. As can be seen, the red emission of **2** weakens with the increasing amount of **3**. The phenomena support the energy transfer from **2** to **3** in the ternary supramolecular copolymers.

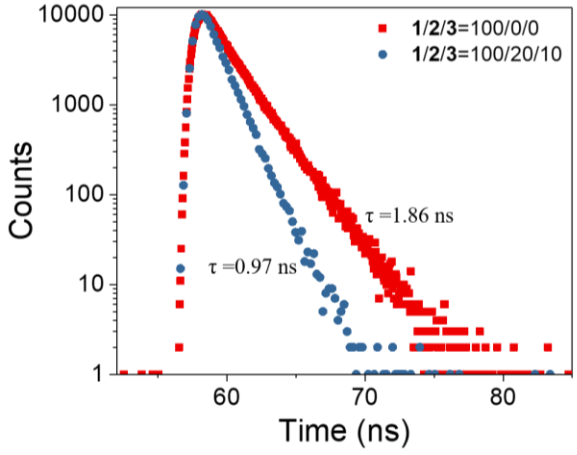

Supplementary Figure 35. Fluorescent lifetime decay curves of **1** at 507 nm (c: 8.0 × 10^{-5} mol L^{-1} for **1**) without and with **2** and **3** (c: 1.6 × 10^{-5} mol L^{-1} for **2**, and 8.0 × 10^{-6} mol/L for **3**). The fluorescent lifetime decay originates from excitation energy transfer from donors **1** to the two acceptors.

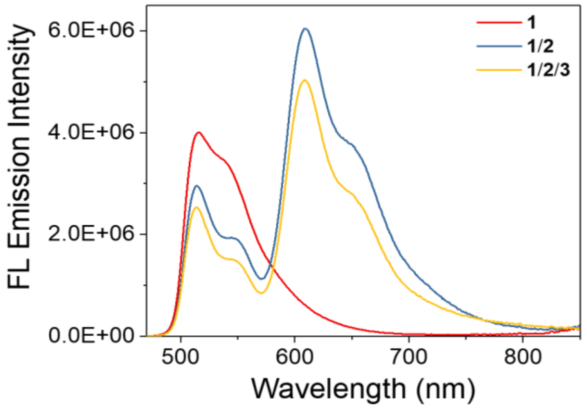

Supplmentary Figure 36. Energy transfer studies for the ternary species **1**–**3** in CH_2Cl_2 (c: 8.0 × 10^{-5} mol L^{-1} for **1**, 1.6 × 10^{-5} mol/L for **2**, and 8.0 × 10^{-6} mol L^{-1} for **3**). When **1**–**3** are mixed in CH_2Cl_2 (dominated by the monomeric state), the energy transfer efficiencies for **1** and **2** are significantly lower than those in MCH (dominated by the supramolecular copolymeric state). Hence, supramolecular copolymerization is a prerequisite for efficient energy transfer.

Determination of sequential energy transfer via transient absorption spectroscopy

Transient absorption spectrum (TAS) is a powerful technique to investigate the sequential energy transfer. To avoid excitation of platinum acetylide units, TAS spectra are recorded upon excitation at 430 nm (The excitation of platinum acetylide units require ultraviolet light)[33]. Notably, the absorption changes (ΔA) of ESA signals exhibit time-dependent spectral evolutions: They reach to the maximal ΔA at 840 ps, followed by continuous attenuation. Special attention is focused on the transient processes occur from 840 ps to 7200 ps. Briefly, two features exist for **1**, **1**/**2** and **1**/**2**/**3** on the nanosecond timescales: a narrow negative band of the ground state bleaching (GSB) centered at 480 nm, and a broad positive band of the excited state absorption (ESA) ranging from 502 nm to 600 nm (Supplementary Figures 37a–c). In terms of supramolecular copolymers **1**/**2**, monomer **2** serves as the relay acceptor, since the

ESA signal centered at 546 nm shows a faster decay on the same timescale (changes for the normalized Δ*A*: 0.074 for **1** and 0.110 for **1**/**2**, Supplementary Figures 37a, b). Likewise, for supramolecular copolymers **1**/**2**/**3** the normalized intensity changes of Δ*A* at 542 nm is 0.129 (Supplementary Figure 37c), which is larger than that of the supramolecular homopolymers derived from **1**. The results support faster deactivation for the ESA band of **1** due to excitation energy transfer.

Additionally, the ESA band ranging in 600−700 nm for supramolecular copolymers **1**/**2** (Supplementary Figure 37b) is assigned to the transient absorption of **2**. The band disappears upon adding **3** as the final acceptor (Supplementary Figure 37c), indicating the decativation for the transient species of **2**. Lifetime changes of the transient species provide the additional evidences. For the binary supramolecular copolymers **1**/**2**, the lifetime for the transient species at 600 nm is 1080 ps. It increases to 1500 ps at 650 nm, and reduces dramatically to 718 ps at 700 nm (Supplementary Figure 37d). By contrast, the ternary supramolecular copolymers **1**/**2**/**3** exhibit direct decay from 1120 ps at 600 nm to 338 ps at 700 nm (Supplementary Figure 37d). The shorter lifetimes of the transient species in **1**/**2**/**3** indicate that the transient species of **2** undergo faster decay, because of the excitation energy transfer from **2** to **3**.

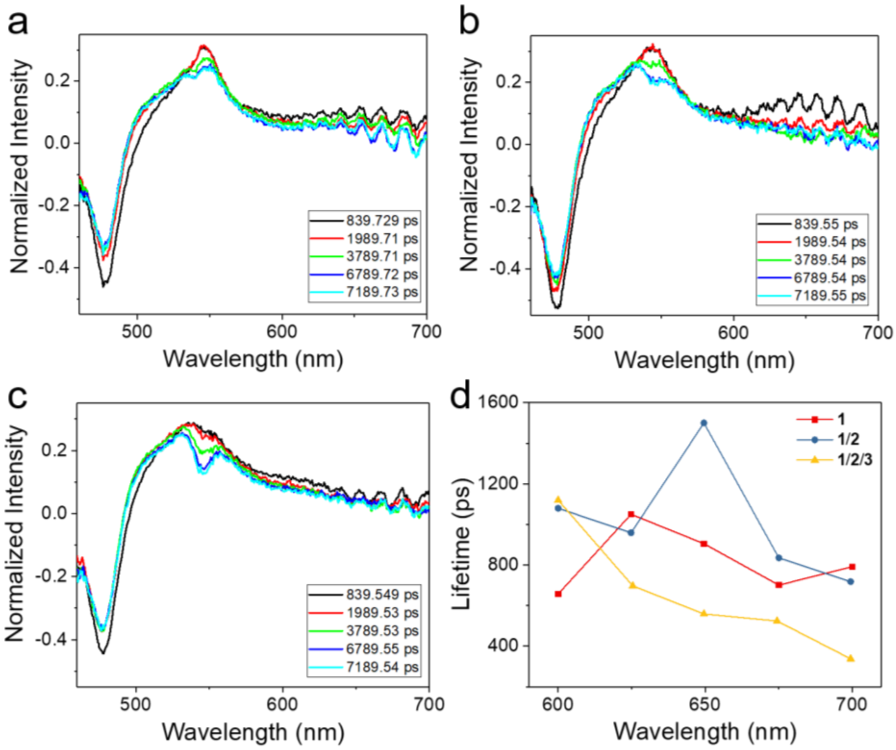

Supplementary Figure 37. Nanosecond TAS measurements for a) supramolecular homopolymers **1** (*c*: 8.0 × 10^{-5} mol L^{-1} for **1** in MCH), b) supramolecular copolymers **1**/**2** (*c*: 8.0 × 10^{-5} mol L^{-1} for **1** and 1.6 × 10^{-5} mol L^{-1} for **2** in MCH), and c) supramolecular copolymers **1**/**2**/**3** (*c*: 8.0 × 10^{-5} mol L^{-1} for **1**, 1.6 × 10^{-5} mol L^{-1} for **2**, 8.0 × 10^{-6} mol L^{-1} for **3** in MCH). d) Comparison for the excited state lifetimes ranging from 600 nm to 700 nm. The lifetimes are obtained by using Surface Xplorer software developed for ultrafast spectroscopy analysis. Two lifetimes are obtained via the software. An extremely fast transient process at picosecond exists for all of the samples, which can be assigned

to solvent reorganizations of the singlet excited states[34]. Lifetimes of the longer transient processes are utilized to illustrate the lifetimes of the transient species.

Determination for energy transfer rates of supramolecular copolymers ***1/2/3***

Förster mechanism: To elucidate the sequential energy transfer behaviors, we calculate the k_{ET} rates for each energy transfer step. According to the supplementary Eq. 6, k_{ET} rate is related to donor lifetime (τ), the Fröster radius (R_0) and the average distance (r) of D/A pair. Accordingly, the ratio of k_{ET} rates can be calculated via the supplementary Eq. 18:

$$(k_{ET,\,\mathbf{1/2}})/(k_{ET,\,\mathbf{1/3}}) = [J(\lambda,\,\mathbf{1/2})/J(\lambda,\,\mathbf{1/3})] \times [(r_{\mathbf{1/3}})/(r_{\mathbf{1/2}})]^6 = 2.53 \approx J(\lambda,\,\mathbf{1/2})/J(\lambda,\,\mathbf{1/3}) = 2.65$$

(**Supplementary Eq. 18**)

In the equation, $J(\lambda,\,\mathbf{1/2})$ and $J(\lambda,\,\mathbf{1/3})$ refer to the spectral overlapping integrals between **1**/**2** and **1**/**3**, while $r_{\mathbf{1/2}}$ and $r_{\mathbf{1/3}}$ stand for the average distances of D/A pairs. The k_{ET} ratio of competitive D/A pairs is almost equal to the ratio of $J(\lambda)$[35]. Since the fluorescence quenching results from energy transfer between **1** and the corresponding acceptors, the energy transfer efficiency of donor can be calculated via the supplementary Eq. 19:

$$\Phi_{ET,\,\mathbf{1/2+1/3}} = (k_{ET,\,\mathbf{1/2}} + k_{ET,\,\mathbf{1/3}})/(k_{r,\,\mathbf{1}} + k_{nr,\,\mathbf{1}} + k_{ET,\,\mathbf{1/2}} + k_{ET,\,\mathbf{1/3}}) = 0.924$$

(**Supplementary Eq. 19**)

In this equation, $k_{r,\mathbf{1}} + k_{nr,\mathbf{1}}$ is the sum of radiative and non-radiative rates in the absence of acceptor, while $k_{ET,\mathbf{1/2}}$ and $k_{ET,\mathbf{1/3}}$ refer to the energy transfer rates of the corresponding D/A pairs in the ternary supramolecular copolymers **1**/**2**/**3**. On this account, by iterating the value of $k_{r,\,\mathbf{1}} + k_{nr,\,\mathbf{1}}$ (τ_0: 1.86 ns) into the above equation, k_{ET} rates of competitive D/A pairs **1**/**2** and **1**/**3** are 4.69×10^9 s^{-1} and 1.85×10^9 s^{-1}, respectively. Similarly, the energy transfer efficiency between **2** and **3** is expressed via the supplementary Eq. 20:

$$\Phi_{ET,\,\mathbf{2/3}} = (k_{ET,\,\mathbf{2/3}})/(k_{r,\,\mathbf{1/2}} + k_{nr,\,\mathbf{1/2}} + k_{ET,\,\mathbf{2/3}}) = 0.925$$

(**Supplementary Eq. 20**)

By iterating the sum of $k_{r,\,\mathbf{1/2}} + k_{nr,\,\mathbf{1/2}}$ of **1**/**2** (τ_0: 18.41 ns for **2**) into the above equation, the k_{ET} rates between **2** and **3** is 6.69×10^8 s^{-1}. It is apparent that the two-step sequential energy transfer plays a major role to the energy transfer from **1** to **3**.

6. *Direct energy transfer from **1** to **3** in the supramolecular copolymeric state*

We also investigated energy transfer from **1** to **3** in supramolecular copolymers **1**/**3**. However, the spectral overlap of **1**/**3** is lower [$J(\lambda)$: 1.90 × 10^{14} M^{-1} cm^{-1} nm^4, Supplementary Figure 38a] than those of **1**/**2** and **2**/**3** [$J(\lambda)$: 5.04 × 10^{14} M^{-1} cm^{-1} nm^4 for **1**/**2** and 2.03 × 10^{15} M^{-1} cm^{-1} nm^4 for **2**/**3**]. As shown in Supplementary Figure 38b, **1**/**3** displays the emission quenching of **1** and concomitant enhancement of NIR emission band upon gradual addition of **3**. At the acceptor ratio of 10 mol%, the fluorescent lifetimes reduce from 1.86 ns to 1.05 ns (Supplementary Figure 38d). Φ_{ET} is determined to be 90.0% (Supplementary Figure 38c), corresponding to a k_{ET} rate of 4.84 × 10^9 s^{-1}.

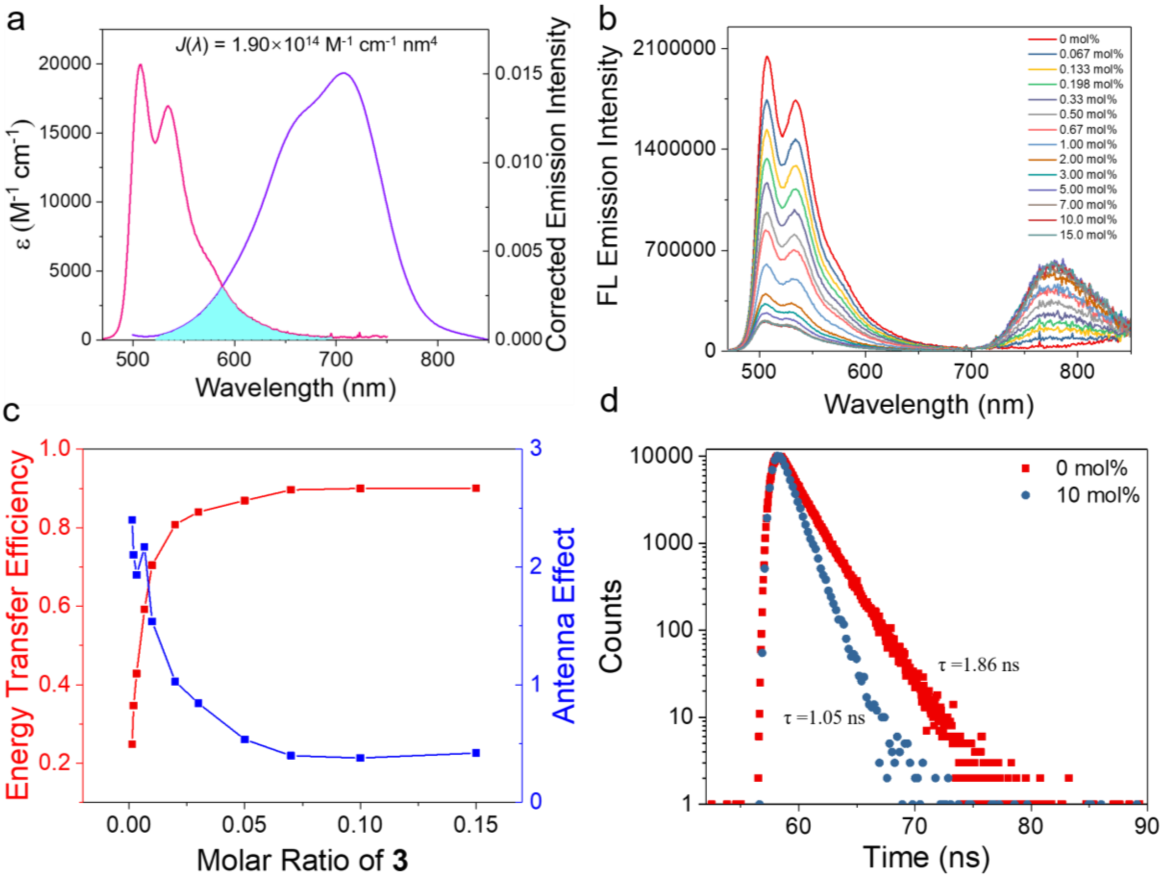

Supplementary Figure 38. a) Spectral overlap between emission of **1** and absorption of **3**. b) Steady-state fluorescence emission changes upon varying the ratios of **3** (c: 8.0 × 10^{-5} mol L^{-1} for **1**). c) Φ_{ET} and the antenna effect of **1**/**3** (c: 8.0 × 10^{-5} mol L^{-1} for **1**) upon increasing the molar ratios of **3**. d) Fluorescence lifetime decay curves for **1** and **1**/**3** at 507 nm.

7. Photo-triggered modulation of sequential energy transfer

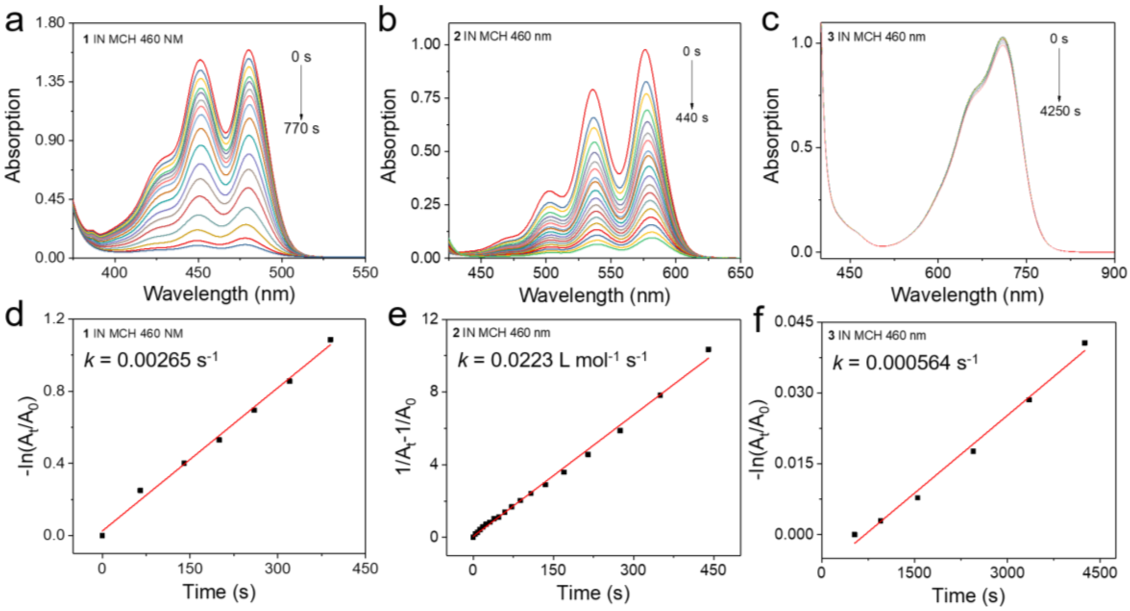

Supplementary Figure 39. Absorption changes of a) **1**, b) **2**, and c) **3** upon 460 nm LED light irradiation (c: 5.0×10^{-5} mol L^{-1} in MCH). Photo-reaction kinetics of d) **1**, e) **2**, and f) **3**. All samples are saturated with air prior to the irradiation experiments. Both compounds **1** and **2** respond sensitively to 460 nm light irradiation and thereby undergoes fast [4+2] endoperoxidation reaction. In comparison, the individual compound **3** shows negligible self-sensitized reactions under 460 nm light irradiation.

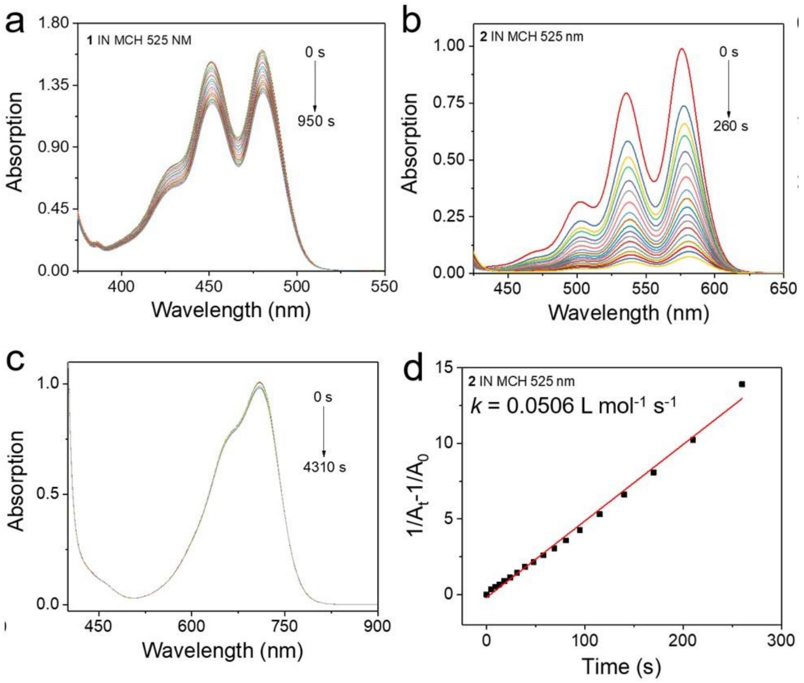

Supplementary Figure 40. Absorption changes of a) **1**, b) **2**, and c) **3** upon 525 nm light irradiation (c: 5.0×10^{-5} mol L^{-1} in MCH). d) Photo-reaction kinetics of **2**. All samples are saturated with air prior to the irradiation experiments. Compound **1** undergoes rather slow endoperoxidation under 525 nm light irradiation. Moreover, the individual compound **3** shows negligible self-sensitized reactions under 525 nm light irradiation. In comparison, compound **2** responds sensitively to 525 nm light irradiation and thereby undergoes fast [4+2] endoperoxidation reaction.

After examining time-dependent absorption changes of supramolecular homopolymers derived from the individual compounds **1**–**3** upon exposure to 460 nm and 525 nm LED lamps, we further studied photo-responsiveness of supramolecular copolymers **1**/**2**/**3**. Under 460 nm LED light irradiation, the photo-chemical kinetics can be divided into two-stages. For the first stage, **2** shows the highest endoperoxidation rate of 0.00225 s^{-1} (t < 1175 s), while the rates for **1** (k_{obs} = 0.00024 s^{-1}, t < 1300 s) and **3** (k_{obs} = 0.00056 s^{-1}, t < 750 s) are significantly lower (Supplementary Figures 41b–d). Accordingly, 93.4% of the relay acceptor **2** are consumed in the first stage, while 25.5% of **1** and 31.9% of **3** participate in the photo-chemical reactions during this timescale. By contrast, in the second stage (t > 1175 s) the photo-chemical reaction rate of **2** decreases to 0.00047 s^{-1}. Meanwhile, the photo-oxygention rates of **1** and **3** increase to 0.00089 s^{-1} (t > 1300 s) and 0.00108 s^{-1} (t > 750 s) (Supplementary Figures 41b–d). With 2130 s light irradiation experiment, the vibronic absorption bands of **2** and **3** almost disappeared, with the presence of the absorption signal of **1** in the final spectrum (Supplementary Figure 41a). It consequently leads to the disruption of sequential energy transfer in supramolecular copolymers **1**/**2**/**3**.

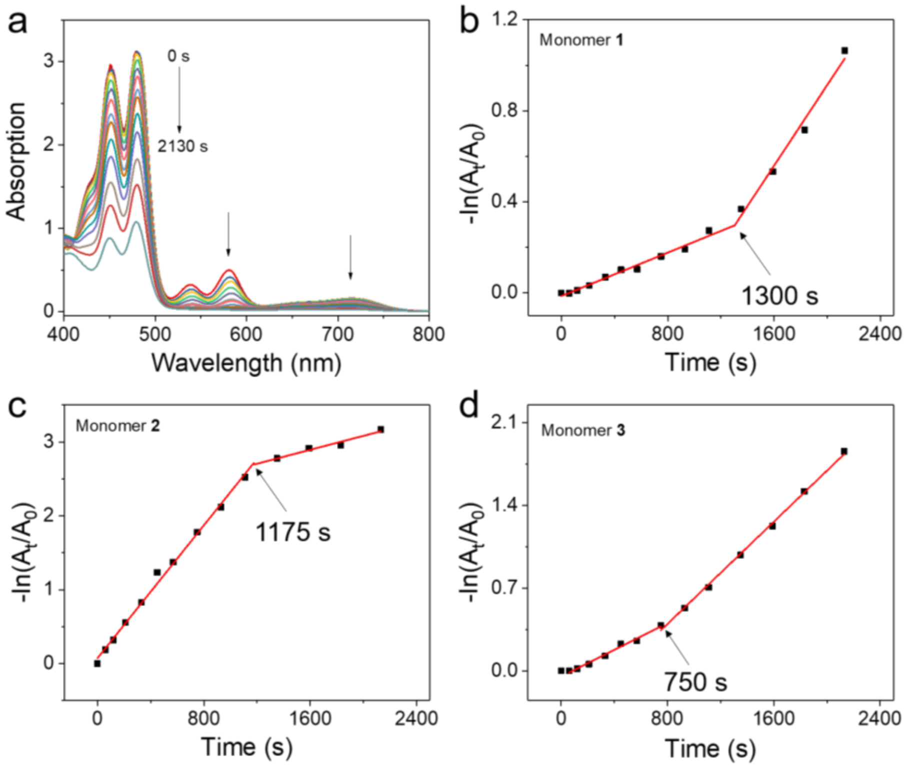

Supplementary Figure 41. a) Time-dependent absorption changes of **1**/**2**/**3** (c: 8 × 10^{-5} mol L^{-1} for **1**, 1.6 × 10^{-5} mol L^{-1} for **2**, and 8 × 10^{-6} mol L^{-1} for **3**) under 460 nm light. The kinetic curves of b) **1**, c) **2**, and d) **3.** Irradiation sample is saturated with air prior to the irradiation experiment. Intriguingly, **3** in the supramolecular copolymeric state displays photo-reactivity enhancement with respect to that of the homopolymeric state (Supplementary Figure 39c).

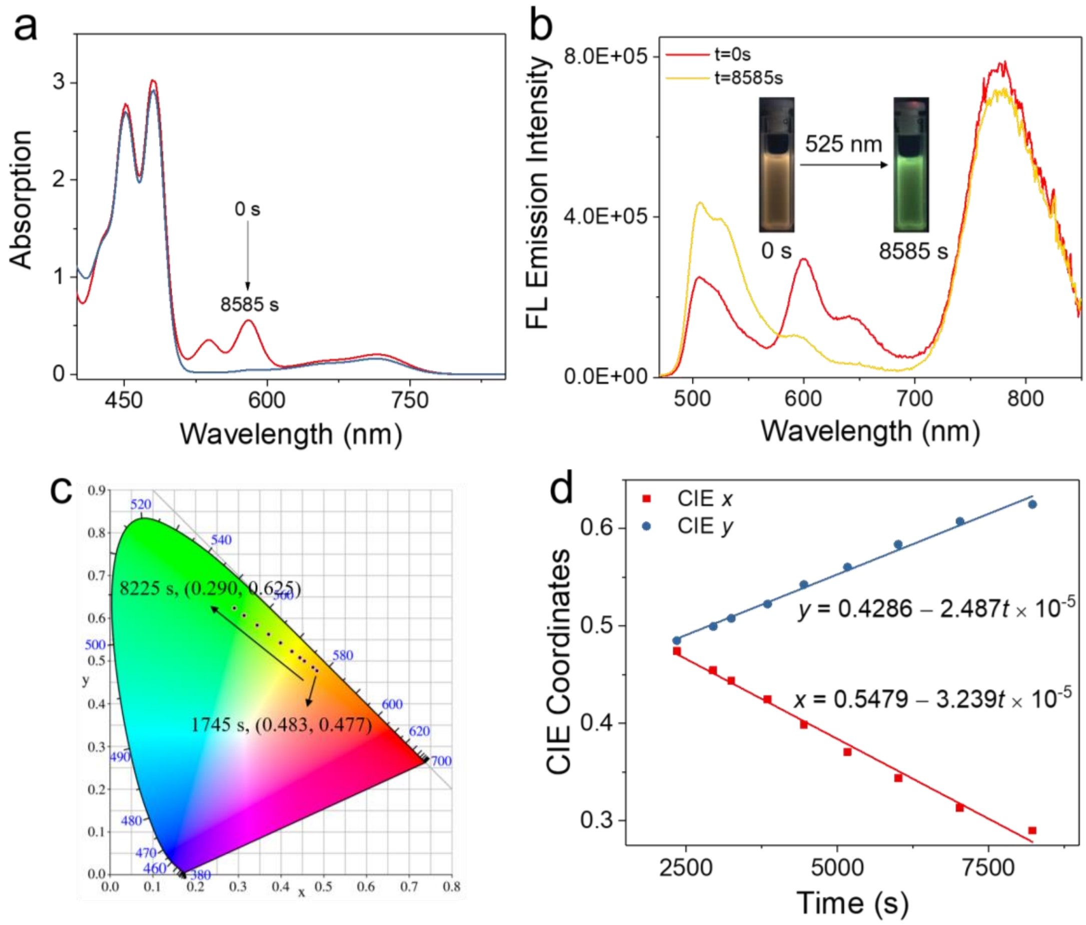

Supplementary Figure 42. a) Absorption signal changes of supramolecular copolymers **1**/**2**/**3** before and after 525 nm LED light irradiation. As can be seen, it selectively endoperoxidizes **2** (reaction ratio after 8585 s: 3.86% for **1**, 91.2% for **2**, and 20.5% for **3**). b) Fluorescent emission spectral changes, and c) CIE coordinate changes upon 525 nm irradiation. d) Linear changes of the CIE (x, y) coordinates versus the irradiation time under 525 nm light. The concentrations are 8.0×10^{-5} mol L^{-1} for **1**, 1.6×10^{-5} mol L^{-1} for **2**, and 8.0×10^{-6} mol L^{-1} for **3** in MCH.

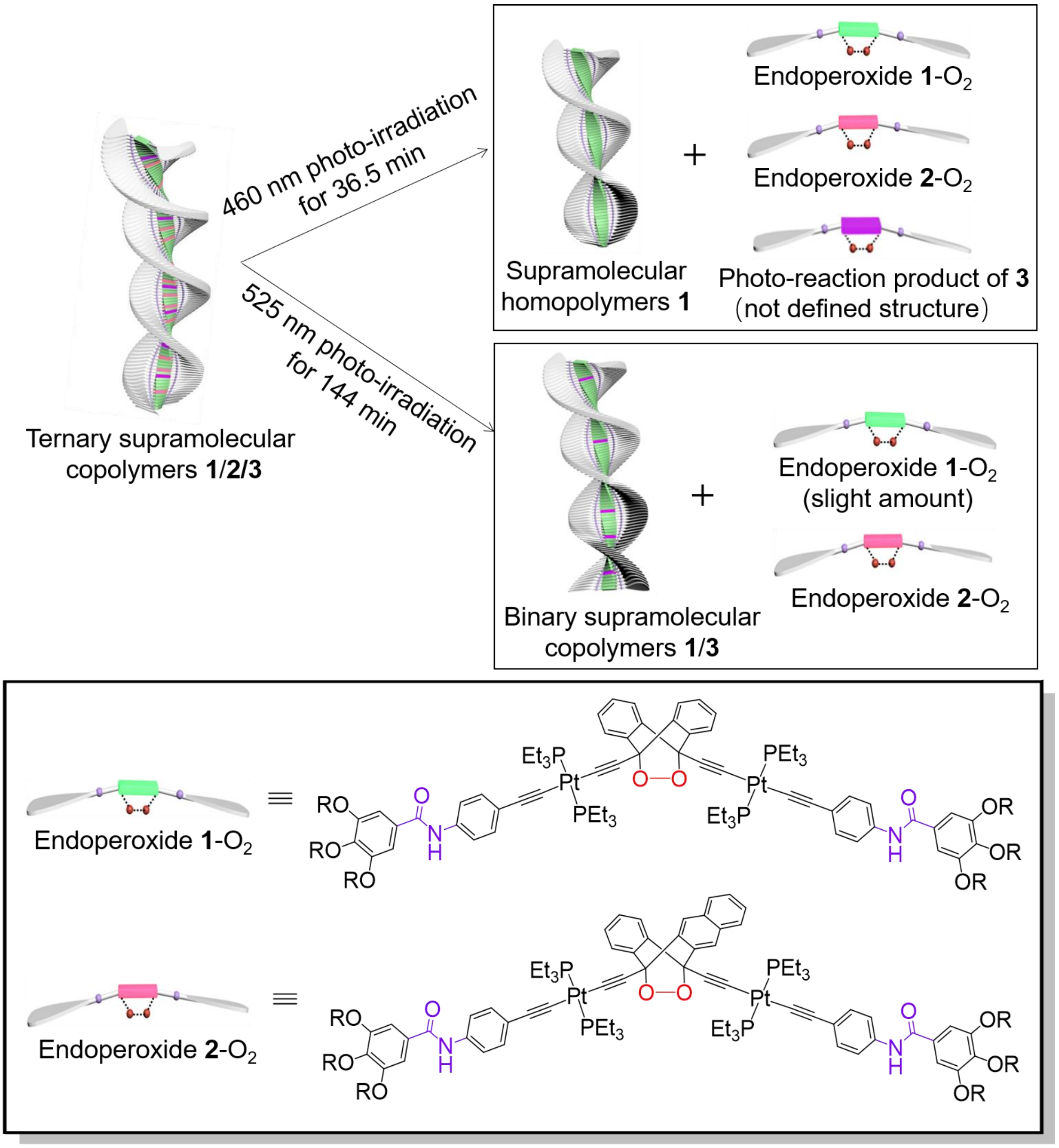

Supplementary Figure 43. Graphic representation for photo-induced structural conversion of supramolecular copolymers **1**/**2**/**3** under 460 nm and 525 nm light irradiation. Since the mechanism for [4+2] photo-oxygenation of naphtho[2,3-*c*][1,2,5]selenadiazole unit is complicated, The exact structure for the product can not be accurately defined. Hence, herein the photo-reaction product of **3** is briefly expressed in the endoperoxidized form in the cartoon picture.

*8. Structural characterization of monomers **2**–**3** and the intermediates*

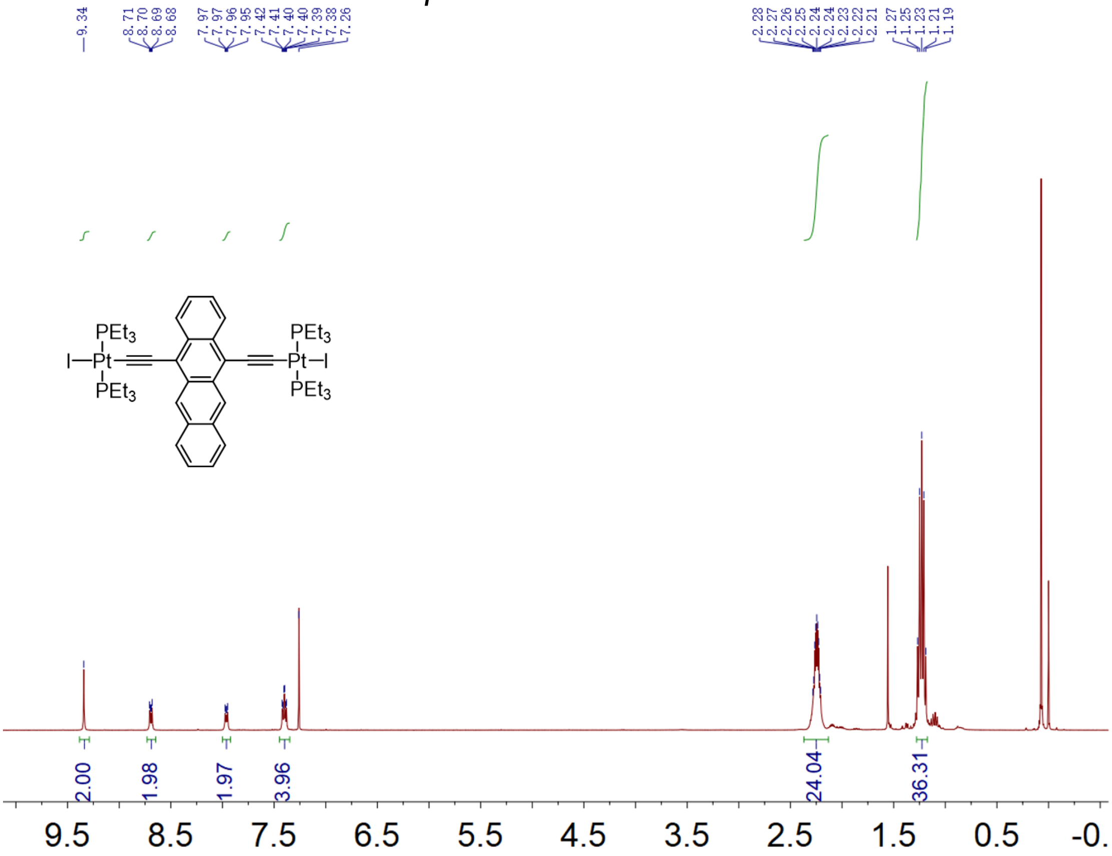

Supplementary Figure 44. ^{1}H NMR spectrum (400 MHz, $CDCl_3$, 298 K) of **5**.

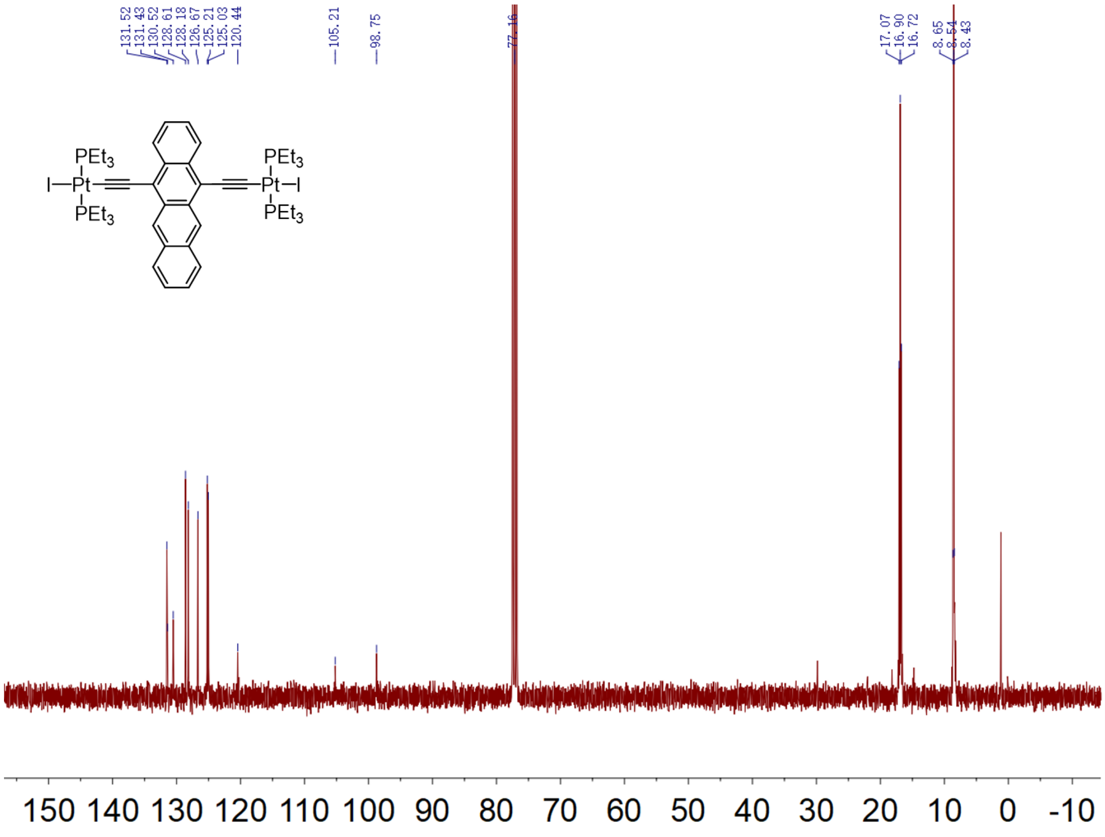

Supplementary Figure 45. ^{13}C NMR spectrum (101 MHz, $CDCl_3$, 298 K) of **5**.

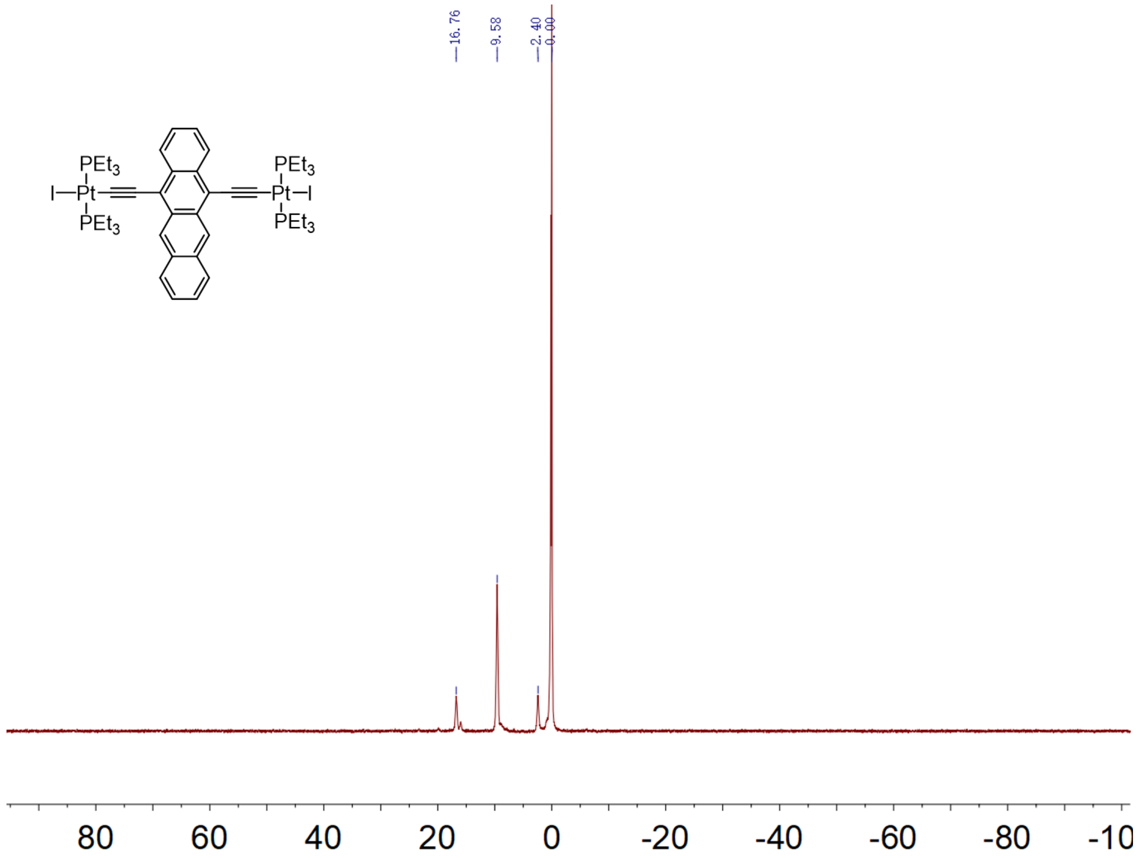

Supplementary Figure 46. ^{31}P NMR spectrum (162 MHz, $CDCl_3$, 298 K) of **5** (85% H_3PO_4 as the internal standard).

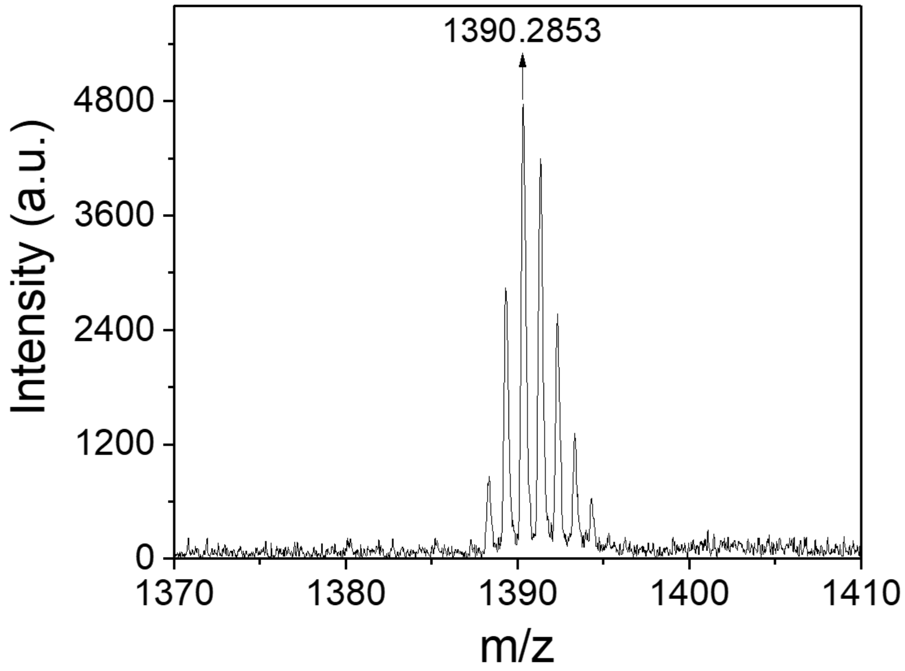

Supplementary Figure 47. MALDI-TOF spectrum of **5**.

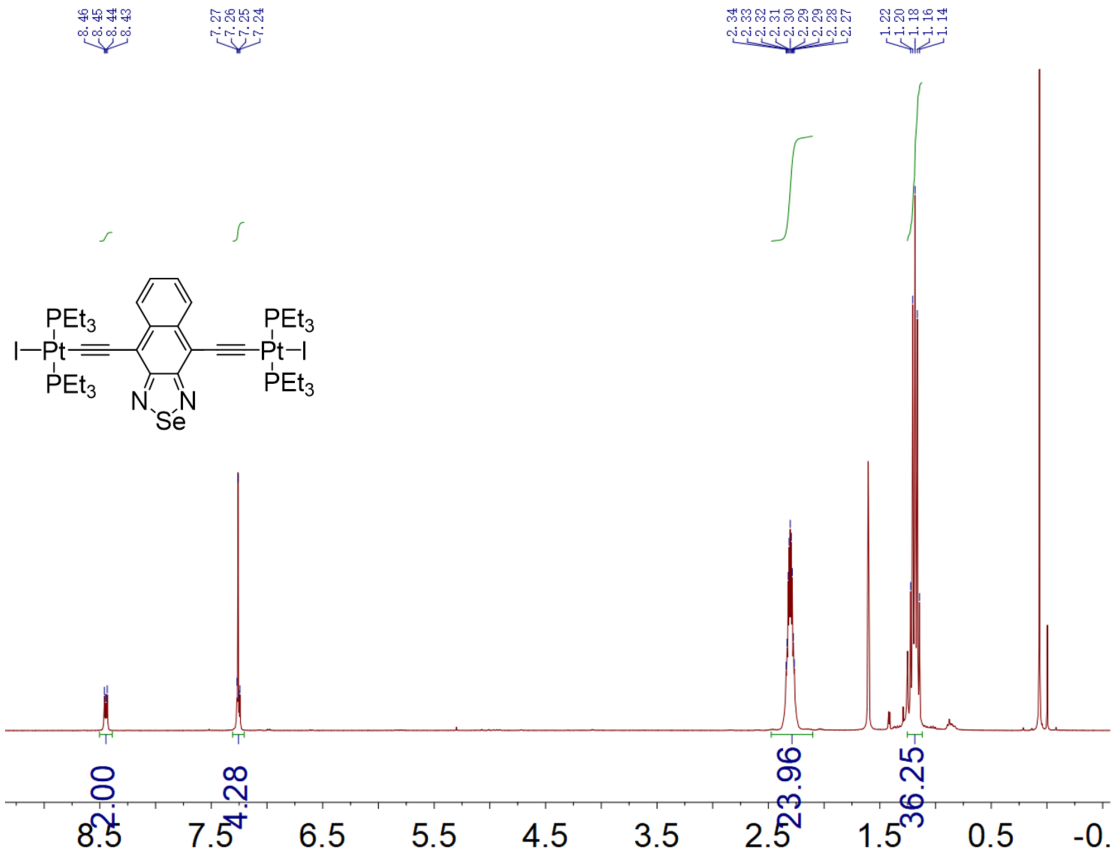

Supplementary Figure 48. ^{1}H NMR spectrum (400 MHz, $CDCl_3$, 298 K) of **6**.

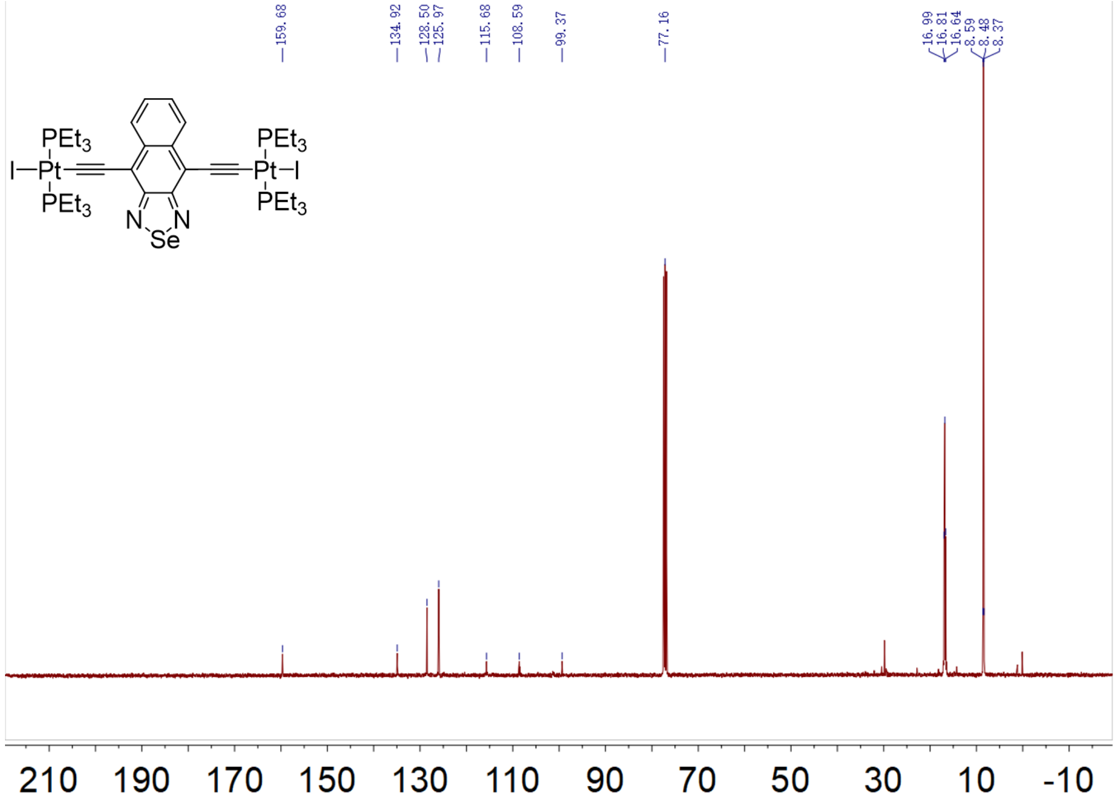

Supplementary Figure 49. ^{13}C NMR spectrum (101 MHz, $CDCl_3$, 298 K) of **6**.

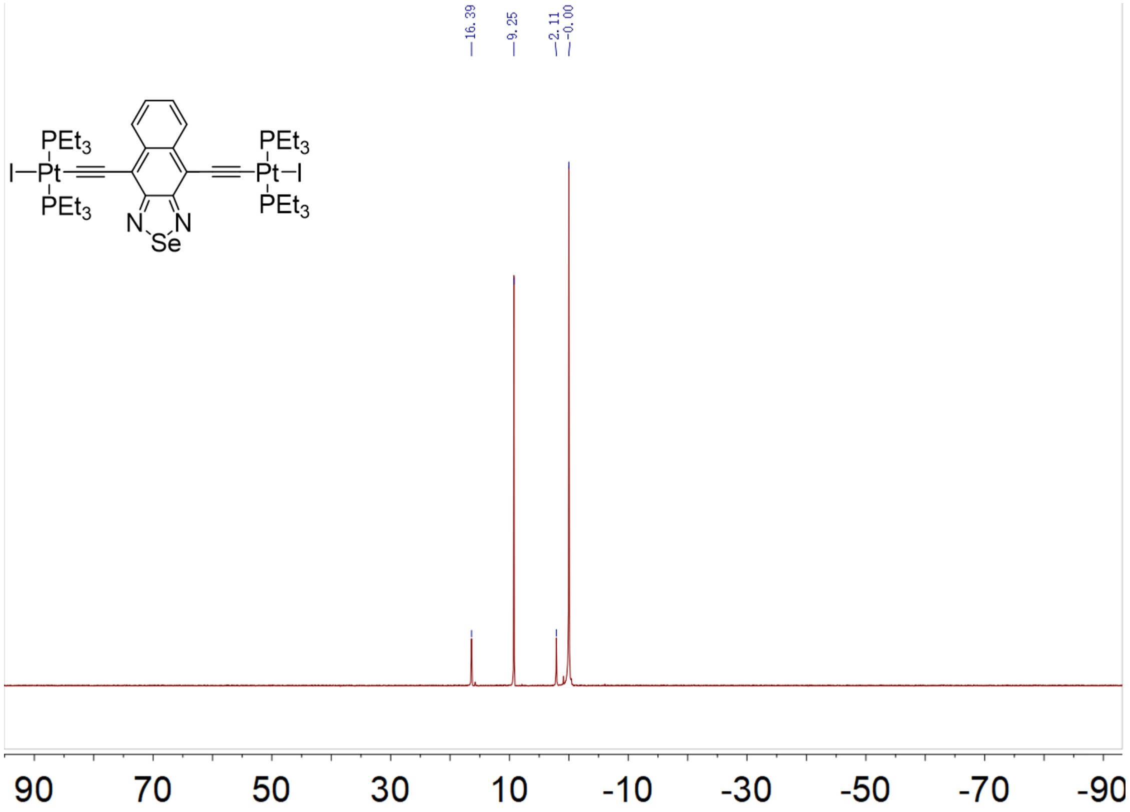

Supplementary Figure 50. ^{31}P NMR spectrum (162 MHz, $CDCl_3$, 298 K) of **6** (85% H_3PO_4 as the internal standard).

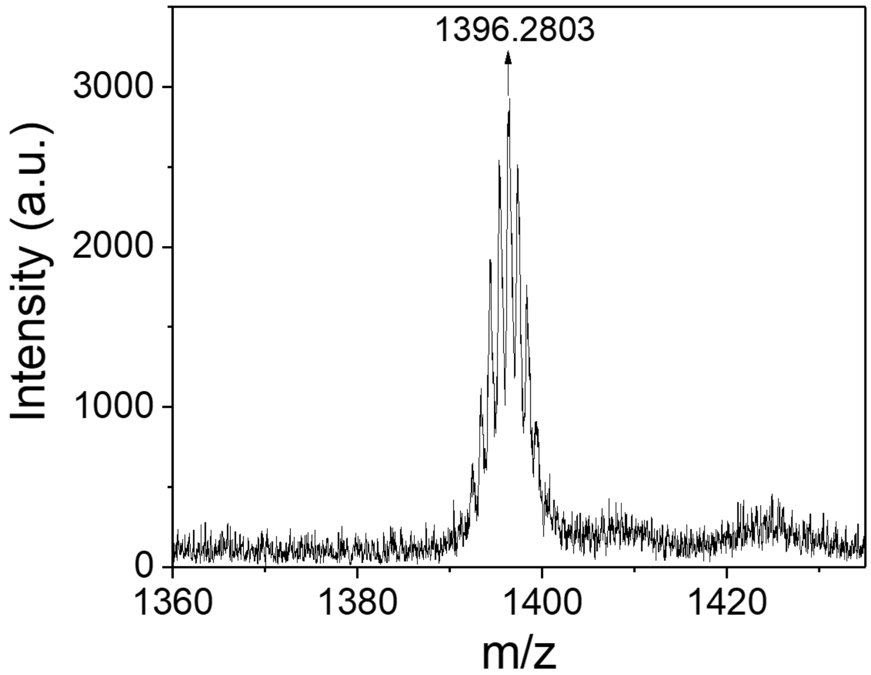

Supplementary Figure 51. MALDI-TOF spectrum of **6**.

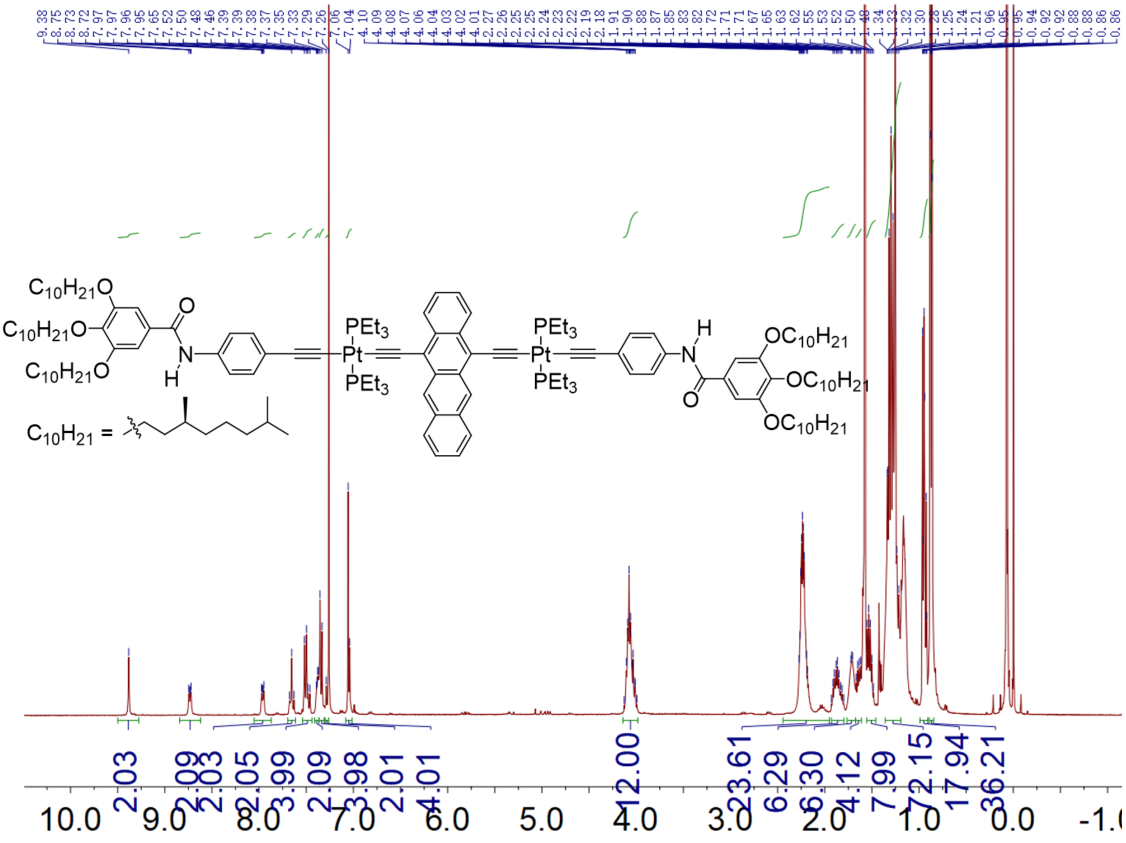

Supplementary Figure 52. ^{1}H NMR spectrum (400 MHz, $CDCl_3$, 298 K) of **2**.

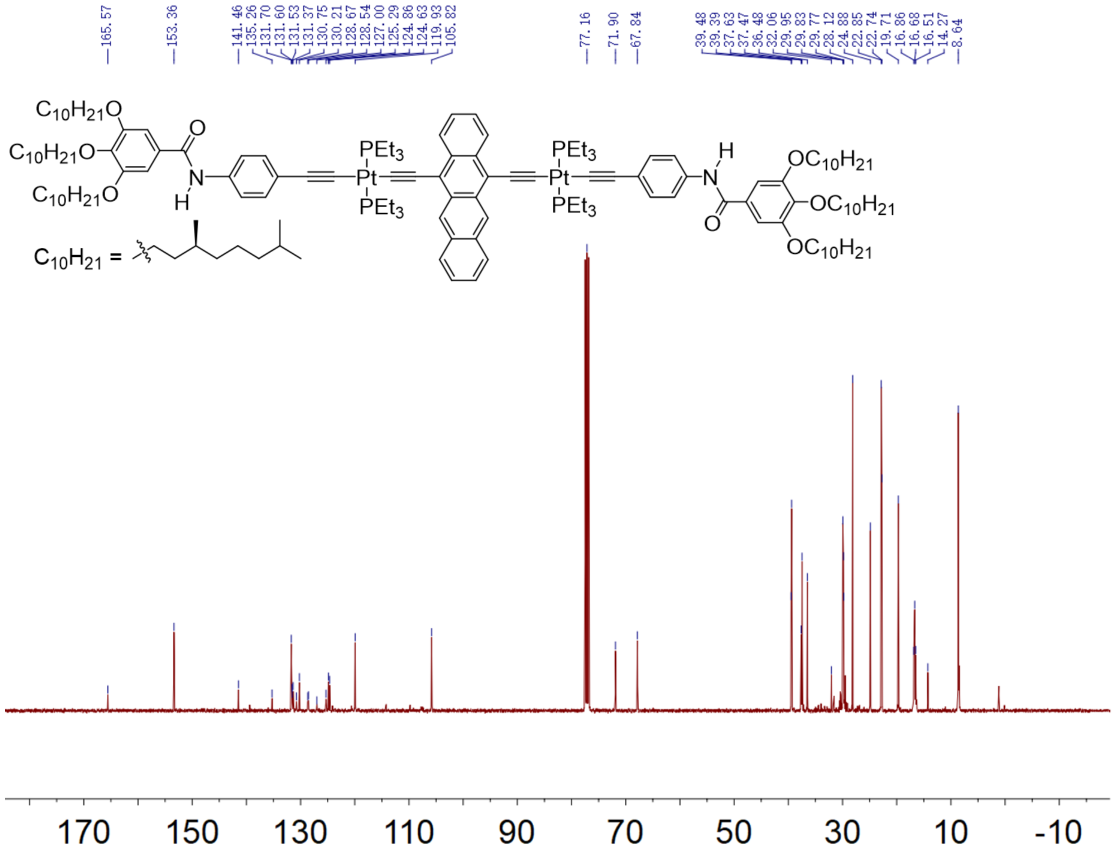

Supplementary Figure 53. ^{13}C NMR spectrum (101 MHz, $CDCl_3$, 298 K) of **2**.

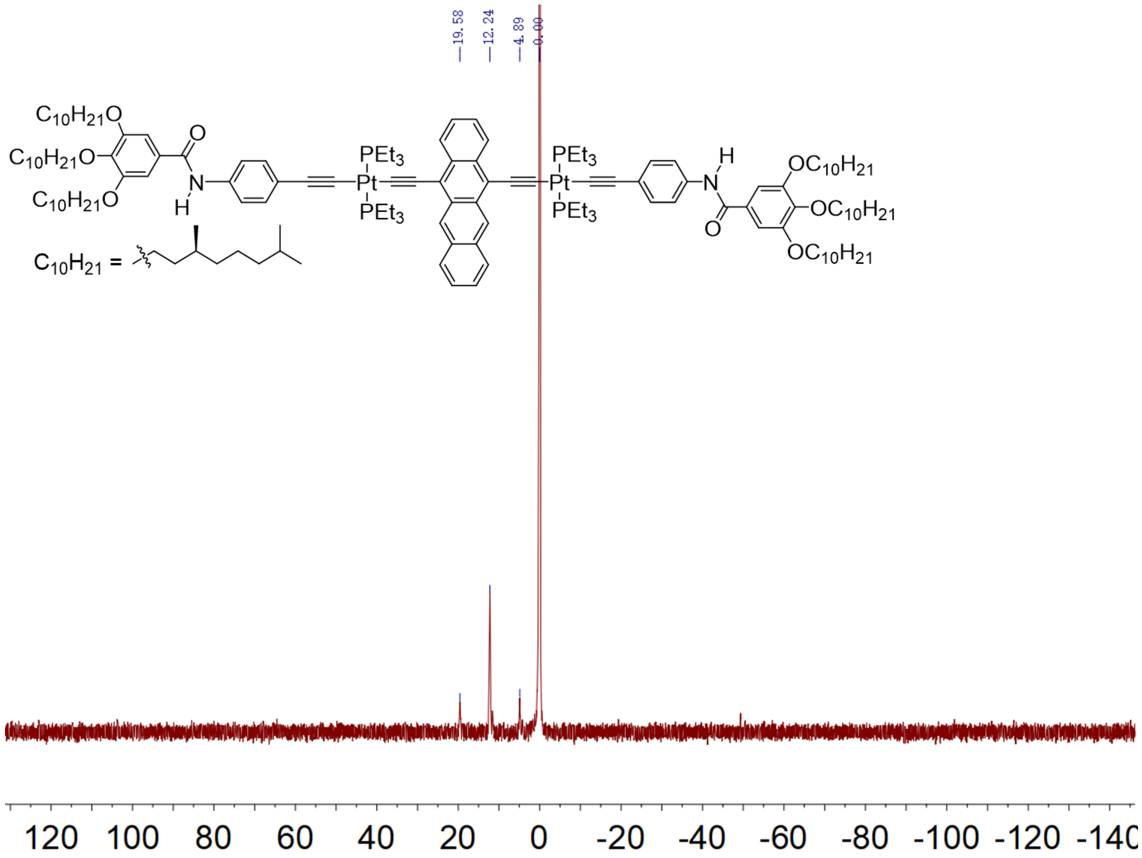

Supplementary Figure 54. ^{31}P NMR spectrum (162 MHz, $CDCl_3$, 298 K) of **2** (85% H_3PO_4 as the internal standard).

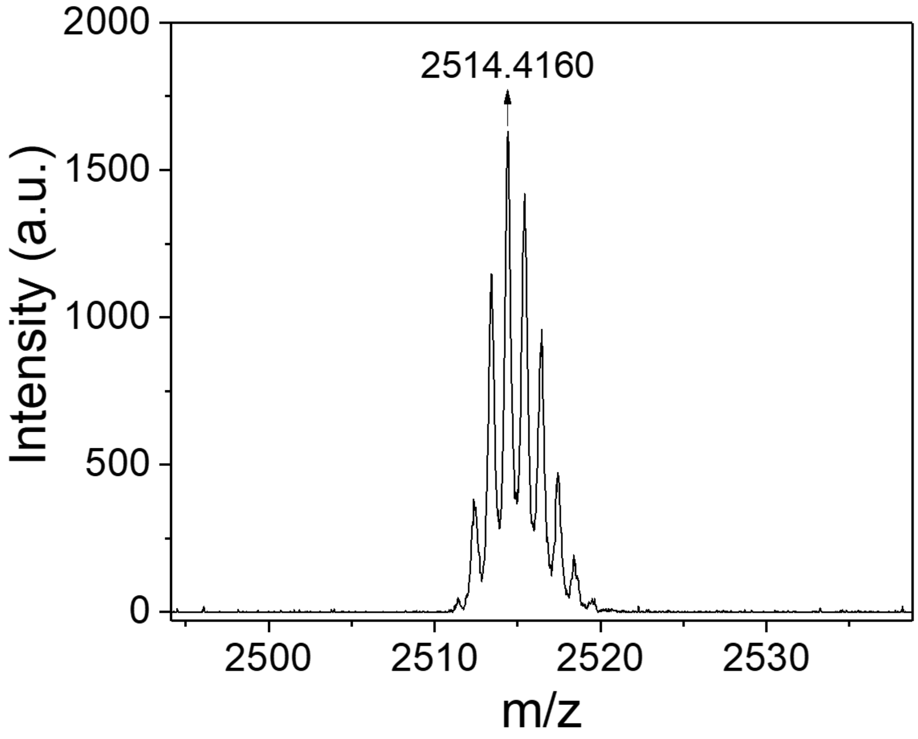

Supplementary Figure 55. MALDI-TOF spectrum of **2**.

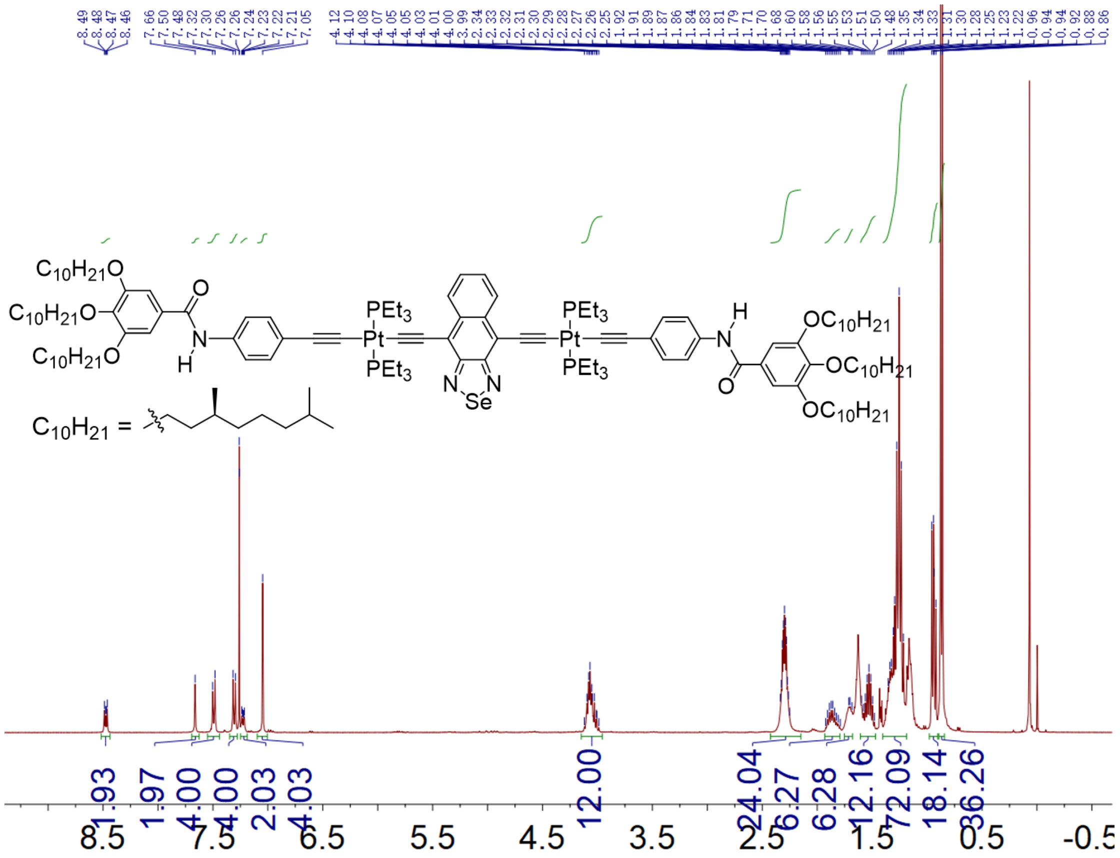

Supplementary Figure 56. ^{1}H NMR spectrum (400 MHz, $CDCl_3$, 298 K) of **3**.

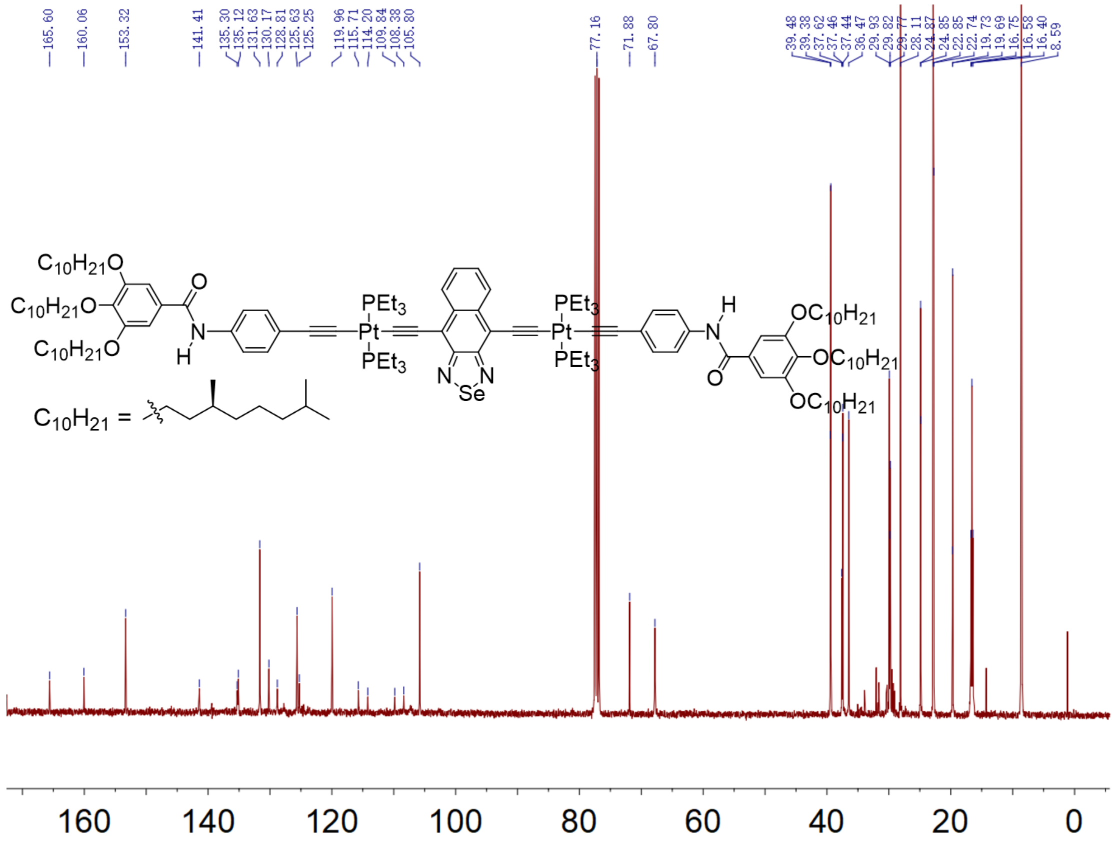

Supplementary Figure 57. ^{13}C NMR spectrum (101 MHz, $CDCl_3$, 298 K) of **3**.

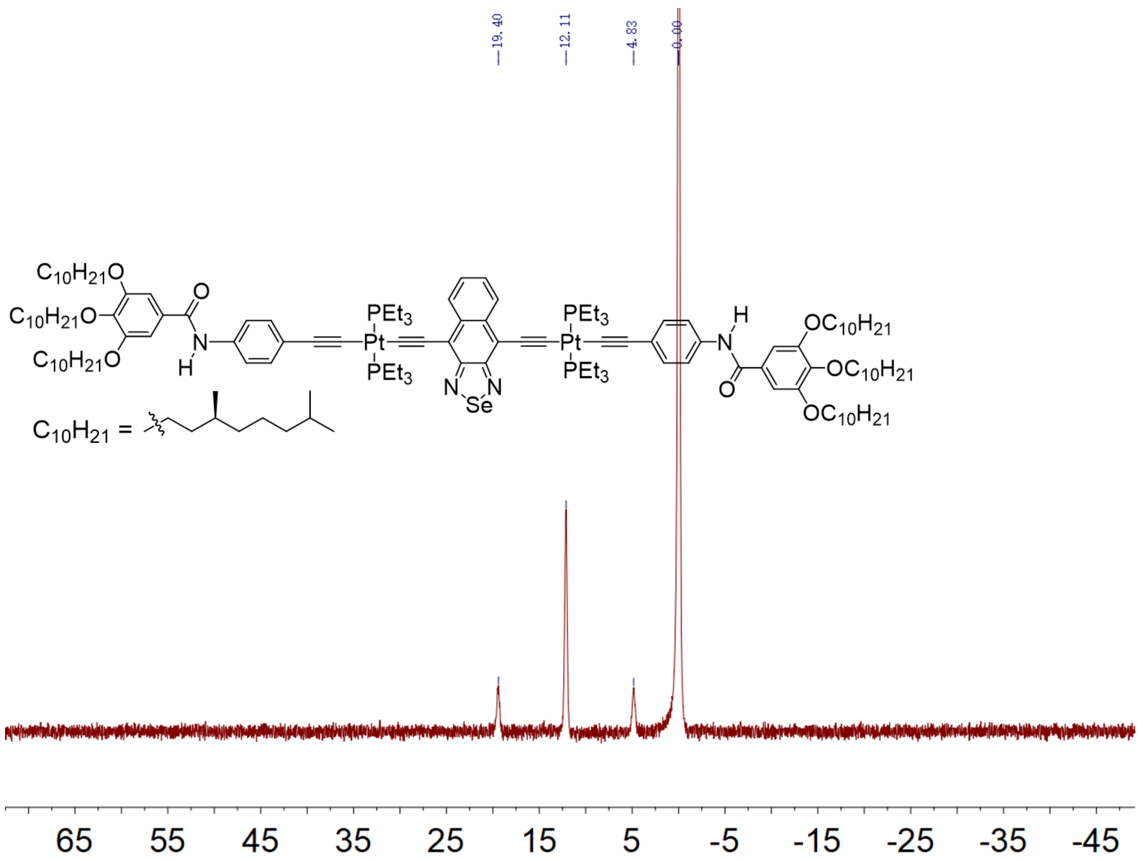

Supplementary Figure 58. ^{31}P NMR spectrum (162 MHz, $CDCl_3$, 298 K) of **3** (85% H_3PO_4 as the internal standard).

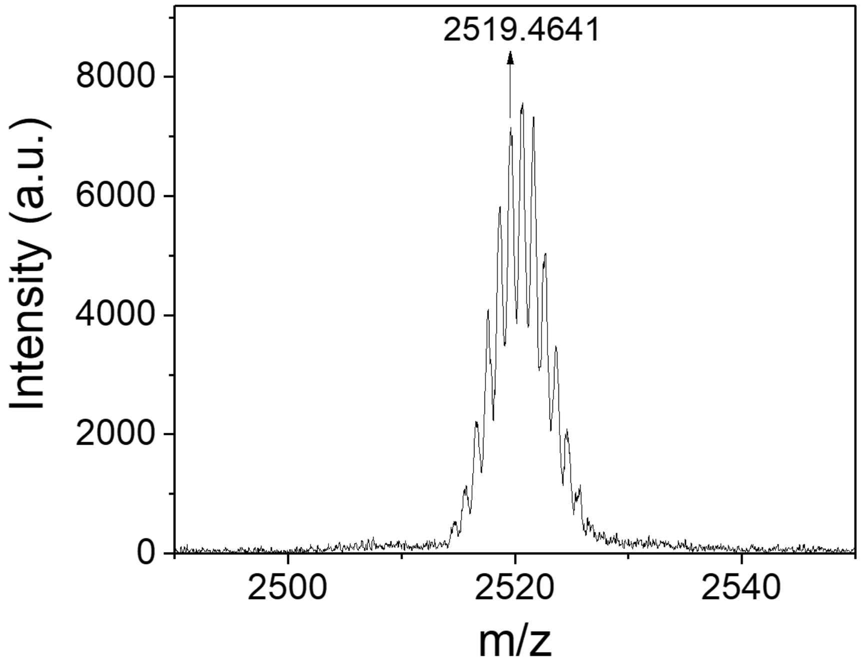

Supplementary Figure 59. MALDI-TOF spectrum of **3**.

Supplementary References:

1. Šembera, F. *et al.* Metal complexes with very large dipole moments: the anionic carborane nitriles 12-NC–$CB_{11}X_{11}$– (X = H, F, CH_3) as ligands on Pt(II) and Pd(II). *Inorg. Chem.* **55**, 3797–3806 (2016).
2. Khan, M. S. *et al*. Synthesis, characterization and optical spectroscopy of platinum(ii) di-ynes and poly-ynes incorporating condensed aromatic spacers in the backbone. *Dalton Trans*. 2377–2385 (2004).
3. Petty II, A. J. *et al*. Ethynylated acene synthesis and photophysics for an organic chemistry laboratory course. *J. Chem. Educ.* **98**, 1741–1749 (2021).
4. Odomm, S. A. *et al.* Tetracene derivatives as potential red emitters for organic LEDs. *Org. Lett.* **5**, 4245–4248 (2003).
5. Lindner, B. D. *et al*. From thia- to selenadiazoles: changing interaction priority. *Org. Lett.* **15**, 666–669 (2013).
6. Gao, Z. *et al*. Cooperative supramolecular polymers with anthracene–endoperoxide photo-switching for fluorescent anti-counterfeiting. *Nat. Commun.* **9**, 3977 (2018).
7. Frisch, M. J. *et al*. Gaussian 09, Revision D.01 (Gaussian, Inc., Wallingford CT, 2013).
8. Rurack, K. *et al*. Fluorescence quantum yields of a series of red and near-infrared dyes emitting at 600–1000 nm. *Anal. Chem.* **83**, 1232–1242 (2011).
9. Karstens, T. *et al*. Rhodamine B and rhodamine 101 as reference substances for fluorescence quantum yield measurements. *J. Phys. Chem.* **84**, 1871–1872 (1980).
10. ten Eikelder, H. M. M. *et al.* An equilibrium model for chiral amplification in supramolecular polymers. *J. Phys. Chem. B* 116, 5291−5301 (2012).
11. ten Eikelder, H. M. M. *et al*. Mass-balance models for scrutinizing supramolecular (co)polymerizations in thermodynamic equilibrium. *Acc. Chem. Res.* **52**, 3465–3474 (2019).
12. Watrob, H. M. *et al*. Two-step FRET as a structural tool. *J. Am. Chem. Soc.* **125**, 7336–7343 (2003).
13. Liu, J. *et al*. FRET study of a trifluorophore-labeled DNAzyme. *J. Am. Chem. Soc.* 124, 15208–15216 (2002).
14. Grynyov, R. S. *et al*. Squaraine dye as an exciton trap for cyanine J-aggregates in a solution. *J. Phys. Chem. C* **112**, 20458–20462 (2008).
15. Chen, P.-Z. *et al*. Light-harvesting systems based on organic nanocrystals to mimic chlorosomes. *Angew. Chem. Int. Ed.* **55**, 2759–2763 (2016).
16. Peng, H.-Q. *et al*. Artificial light-harvesting system based on multifunctional surface-cross-linked micelles. *Angew. Chem. Int. Ed.* **51**, 2088–2092 (2012).
17. Nguyen, M.-H. *et al*. Ligand perturbations on fluorescence of dinuclear platinum complexes of 5,12-diethynyltetracene: a spectroscopic and computational study. *Organometallics* **32**, 1620−1629 (2013).
18. Jiang, B. *et al.* A series of new star-shaped or branched platinum–acetylide derivatives: synthesis, characterization, and their aggregation behavior. *Chem. Commun.* **49**, 6977−6979 (2013).
19. Li, Z. *et al*. Novel platinum–acetylide metallocycles constructed via a stepwise fragment coupling approach and their aggregation behaviour. *Chem. Commun.* **49**, 6194−6196 (2013).
20. Harvey, P. D. Organometallic and coordination polymers, and linear and star oligomers using the *trans*-$Pt(PR_3)_2(C{\equiv}C)_2$ linker. *J. Inorg. Organomet. Polym.* **27**, 3−38 (2017).
21. Bonnot, A. *et al*. Pentacene- and BODIPY-containing *trans*-bis(ethynyl)bis(phosphine)platinum(II) organometallic polymers: a DFT point of view. *J. Inorg. Organomet. Polym.* **26**, 1328–1337 (2016).
22. Nguyen, M.-H. *et al*. Platinum-conjugated homo- and heterobichromophoric complexes of tetracene and pentacene. *Organometallics* **31**, 7522−7531 (2012).

23. Nguyen, M.-H. *et al*. Sequence-specific synthesis of platinum-conjugated trichromophoric energy cascades of anthracene, tetracene, and pentacene and fluorescent “black chromophores”. *Organometallics* **32**, 7283−7291 (2013).
24. Nguyen, M.-H. *et al*. Pushing pentacene-based fluorescence to the near-infrared region by platination. *Organometallics* **30**, 6383−6392 (2011).
25. Han, Y. *et al*. Photoresponsiveness of anthracene-based supramolecular polymers regulated via a σ-platinated 4,4-difluoro-4-bora-3a,4a-diaza-*s*-indacene photosensitizer. *Inorg. Chem.* **58**, 12407−12414 (2019).
26. Tanaka, M. *et al*. Self-assembly and gelation behavior of tris(phenylisoxazolyl)benzenes. *J. Org. Chem.* **76**, 5082–5091 (2011).
27. Ikeda, T. *et al*. Cooperative self-assembly of carbazole derivatives driven by multiple dipole−dipole interactions. *J. Org. Chem.* **81**, 6832–6837 (2016).
28. Zhang, X. *et al*. Single-crystal organic microtubes with a rectangular cross section. *Angew. Chem. Int. Ed.* **46**, 1525–1528 (2007).
29. Jordan, B. J. *et al*. Controlled self-assembly of organic nanowires and platelets using dipolar and hydrogen-bonding interactions. *Small* **4**, 2074–2078 (2008).
30. Sarkar, A. *et al*. Self-sorted, random, and block supramolecular copolymers via sequence controlled, multicomponent self-assembly. *J. Am. Chem. Soc.* **142**, 7606−7617 (2020).
31. Giansante, C. *et al*. Exploiting direct and cascade energy transfer for color-tunable and white-light emission in three-component self-assembled nanofibers. *J. Phys. Chem. C* **116**, 21706−21716 (2012).
32. Sorokin, A. V. *et al*. Control of exciton migration efficiency in disordered J-aggregates. *J. Phys. Chem. C* **114**, 1299–1305 (2010).
33. Li, Y. *et al*. Intramolecular triplet energy transfer in anthracene-based platinum acetylide oligomers. *J. Phys. Chem. B* **117**, 9025−9033 (2013).
34. Carlotti, B. *et al*. Comprehensive photophysical behaviour of ethynyl fluorenes and ethynyl anthracenes investigated by fast and ultrafast time-resolved spectroscopy. *ChemPhysChem* **13**, 724–735 (2012).
35. Song, Q. *et al*. Efficient artificial light-harvesting system based on supramolecular peptide nanotubes in water. *J. Am. Chem. Soc.* **143**, 382–389 (2021).
